# Paneth Cells Protect against Acute Pancreatitis via Modulating Gut Microbiota Dysbiosis

**DOI:** 10.1128/msystems.01507-21

**Published:** 2022-05-02

**Authors:** Yang Fu, Qixiang Mei, Nuoming Yin, Zehua Huang, Baiwen Li, Shengzheng Luo, Binqiang Xu, Junjie Fan, Chunlan Huang, Yue Zeng

**Affiliations:** a Shanghai Key Laboratory of Pancreatic Disease, Shanghai JiaoTong University School of Medicine, Shanghai, China; b Department of Gastroenterology, Shanghai General Hospital, Shanghai JiaoTong University School of Medicine, Shanghai, China; Pacific Northwest National Laboratory; Nanyang Technological University; Duke-Nus Medical School

**Keywords:** acute pancreatitis, Paneth cell, gut microbiota, lysozyme, intestinal enteroid

## Abstract

Acute pancreatitis (AP) is usually accompanied by intestinal failure, but its mechanism is still unclear. In AP patients, the functions of Paneth cells (lysozyme, HD5, Reg3γ, and Wnt3a) decreased. Compared with AP mice, injuries and inflammation of the pancreas and ileum were aggravated in AP mice treated with dithizone (Dith) (Dith+AP mice). Intestinal permeability and bacterial translocation were also increased. 16S rRNA sequencing showed that the gut microbiota of Dith mice and Dith+AP mice exhibited a marked increase in the pathogenic bacterium *Helicobacter* and a significant decrease in the probiotic bacterium *Blautia*. Lysozyme gavage in Dith+AP mice effectively alleviated injuries of the pancreas and small intestine. The beneficial effect of lysozyme was associated with a significant increase in the probiotic bacterium *Blautia* and a virtual absence of the pathogenic bacterium *Helicobacter*. The severity of AP in antibiotic-treated mice (ABX mice) was significantly aggravated when receiving feces from Dith mice and was markedly alleviated when receiving feces from lysozyme-gavaged mice. *In vitro*, lysozyme increased the proliferation of enteroids by promoting the activation of the Wnt pathway and Lgr5 expression in intestinal stem cells.

**IMPORTANCE** We demonstrate that AP patients and experimental AP mice exhibited a dysfunction of Paneth cells. Our *in vivo* research showed that the severity of AP was exacerbated by the long-term dysfunction of Paneth cells, which was associated with gut microbiota disorder. Restoring part of Paneth cell functions through lysozyme supplementation alleviated the severity of AP and gut microbiota dysbiosis. This study provides novel insight into the link of pancreas-gut interactions in the pathogenesis of AP, providing a new direction for the clinical treatment of intestinal complications during AP.

## INTRODUCTION

Acute pancreatitis (AP) is one of the most common gastrointestinal diseases requiring urgent hospitalization (1). Approximately 20 to 30% of patients develop severe acute pancreatitis (SAP) with a substantial mortality rate of 20 to 40% (2). The translocation of intestinal bacteria and endotoxin after intestinal barrier injury is a key event leading to SAP (3). A growing number of studies revealed that intestinal microecology alteration is related to the development of AP, which includes microbiota dysbiosis, intestinal barrier damage, and immunological dysfunction (3–6). But the mechanisms have not yet been well understood and require further elucidation.

Paneth cells are highly differentiated secretory cells in the intestinal epithelium (7). They are distributed in the intestinal crypts and play an important role in the intestinal barrier. These cells secrete antimicrobial peptides (AMPs) such as lysozyme (Lyz) and α-defensin to maintain the homeostasis of the intestinal environment (8, 9). Paneth cells also serve as guardians of intestinal stem cells via providing essential cytokines such as Wnt3a and transforming growth factor β (TGFβ) (10). Their abnormality is related to the progression of a variety of diseases (11–13). Our previous study proved that the transient ablation of Paneth cells by dithizone (Dith) aggravated pancreatic and intestinal injuries in rat AP (14). An interaction exists between the gut microbiota and Paneth cells. Mice lacking the intestinal Sox9 protein presented an absence of Paneth cells accompanied by increases in *Bacteroidetes* and *Enterococcus* and a decrease in *Bifidobacterium* (15), while the gut microbiota regulates Paneth cell numbers and functions (16).

In this study, we explored the role of the gut microbiota regulated by Paneth cells in AP and the potential therapeutic effects of lysozyme on AP by *in vivo* and *in vitro* experiments.

## RESULTS

### Dysfunction of Paneth cells in AP patients and experimental AP mice.

**FIG 1:**
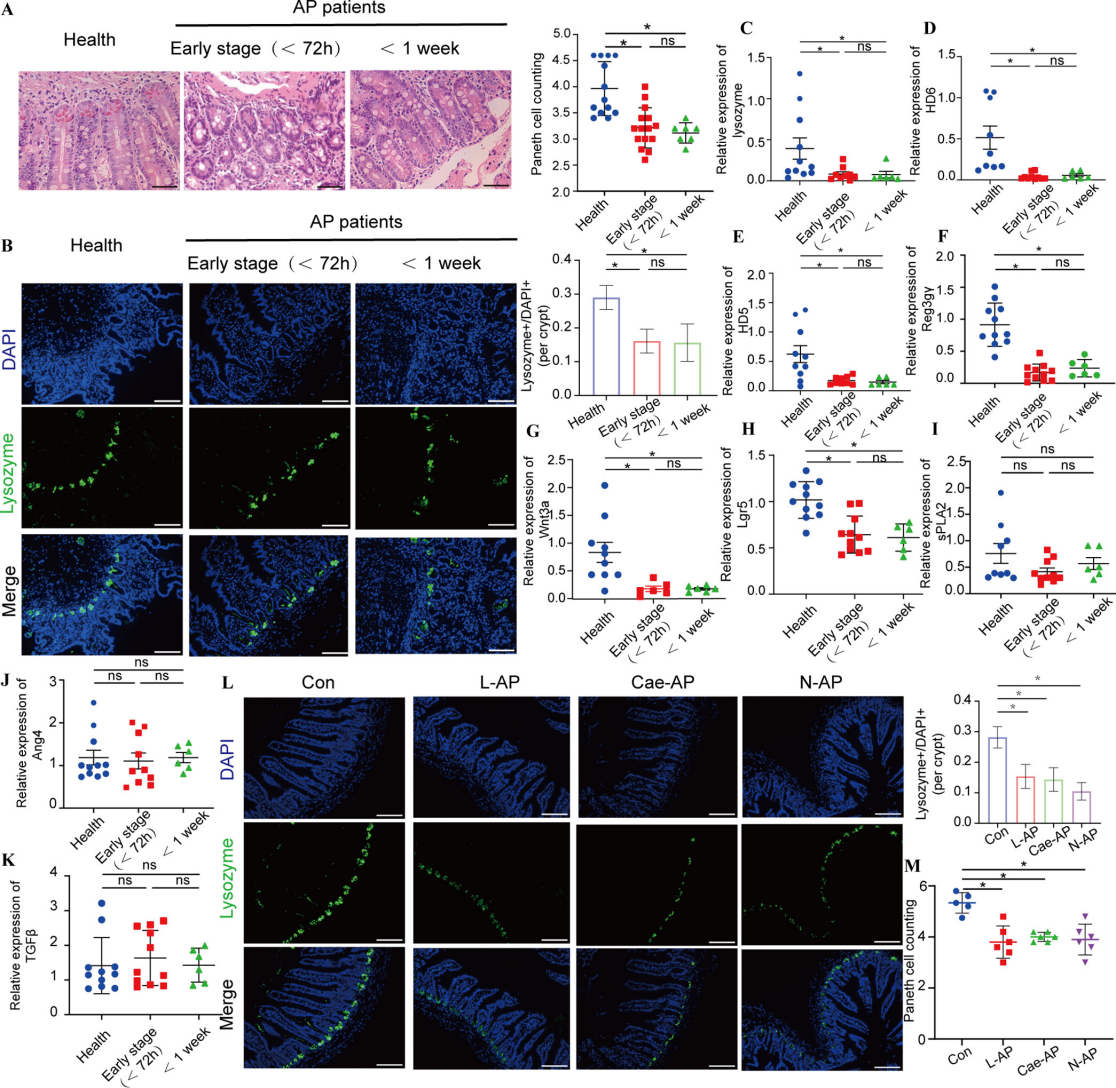
AP patients and experimental AP mice presented Paneth cell defects. (A) Histopathological changes and mean numbers of Paneth cells per crypt of duodenal mucosa specimens were assessed by H&E staining. Original magnification, ×200 (*n* = 7 to 14 individuals per group). (B) Lysozyme expression (green) was assessed in Paneth cells of duodenal mucosa specimens by immunofluorescence (magnification, ×200) and lysozyme-positive/DAPI^+^ quantification. (C to K) The mRNA expression levels of lysozyme (C), HD6 (D), HD5 (E), Reg3γ (F), Ang4 (G), sPLA2 (H), TGFβ (I), Wnt3a (J), and Lgr5 (K) were assessed. (L) Lysozyme expression (green) (magnification, ×200) and lysozyme-positive/DAPI^+^ quantification in three AP models. (M) Mean number of Paneth cells per crypt in AP models. The data are presented as the means ± SD. ns, no significant difference; *, *P ≤ *0.05.

We collected duodenal mucosa specimens by endoscopy from 21 AP patients and 14 healthy controls. AP patients were divided into two groups based on the course of the disease to explore whether the changes in Paneth cells were associated with the course of AP. No demographic differences were found among the three groups (see Table S1 in the supplemental material). Compared with healthy controls, Paneth cell counts and protein expression levels of lysozyme in the duodena were significantly decreased (*P < *0.05) in AP patients in the early stage (<72 h) or with an onset time of <1 week (Fig. 1A and B). AP patients in the early stage had lower (*P < *0.05) mRNA expression levels of lysozyme, human defensin 5 (HD5), HD6, regenerating islet-derived protein 3γ (Reg3γ), Wnt3a, and Lgr5 than did healthy controls (Fig. 1C to H). Significantly reduced mRNA expression levels (*P < *0.05) of lysozyme, HD5, HD6, Reg3γ, and Wnt3a were also found in AP patients with an onset time of <1 week (Fig. 1C to H). However, compared with the healthy controls, the expression levels of angiogenin 4 (Ang4), secretory phospholipase A2 (sPLA2), and TGFβ did not change greatly, no matter how long the disease lasted (Fig. 1I to K). Therefore, dysfunctions of Paneth cells were detected in AP patients regardless of the course of the disease.

10.1128/msystems.01507-21.8 TABLE S1 Clinical and demographic characteristics of AP patients. Download Table S1, DOCX file, 0.01 MB. Copyright © 2022 Fu et al. 2022 Fu et al. https://creativecommons.org/licenses/by/4.0/ This content is distributed under the terms of the Creative Commons Attribution 4.0 International license.

We applied three classical mouse models of AP, which were the L-arginine model (L-AP), the cerulein-plus-lipopolysaccharide (LPS) model (Cae-AP), and the Na-taurocholate model (N-AP), to further validate these findings. Compared with the control (Con) mice, the number of Paneth cells in crypts and the expression of lysozyme decreased significantly (*P < *0.05) in the three AP models (Fig. 1L and M). Lysozyme, α-defensin 5 (Defa5), Reg3γ, Wnt3a, Lgr5, and TGFβ were reduced at the mRNA level (Fig. S1A and B).

10.1128/msystems.01507-21.1 FIG S1 Dysfunction of Paneth cells was revealed by the mRNA expression levels of Defa5, lysozyme, sPLA2, and Reg3γ (A) and Lgr5, Wnt3a, and TGFβ (B) in three different models of AP. The data are presented as the means ± SD. ns, no significant difference; *, *P ≤ *0.05. Download FIG S1, TIF file, 0.7 MB. Copyright © 2022 Fu et al. 2022 Fu et al. https://creativecommons.org/licenses/by/4.0/ This content is distributed under the terms of the Creative Commons Attribution 4.0 International license.

### Long-term reduction of Paneth cells aggravated AP-induced injuries, inflammation, and bacterial translocation.

**FIG 2:**
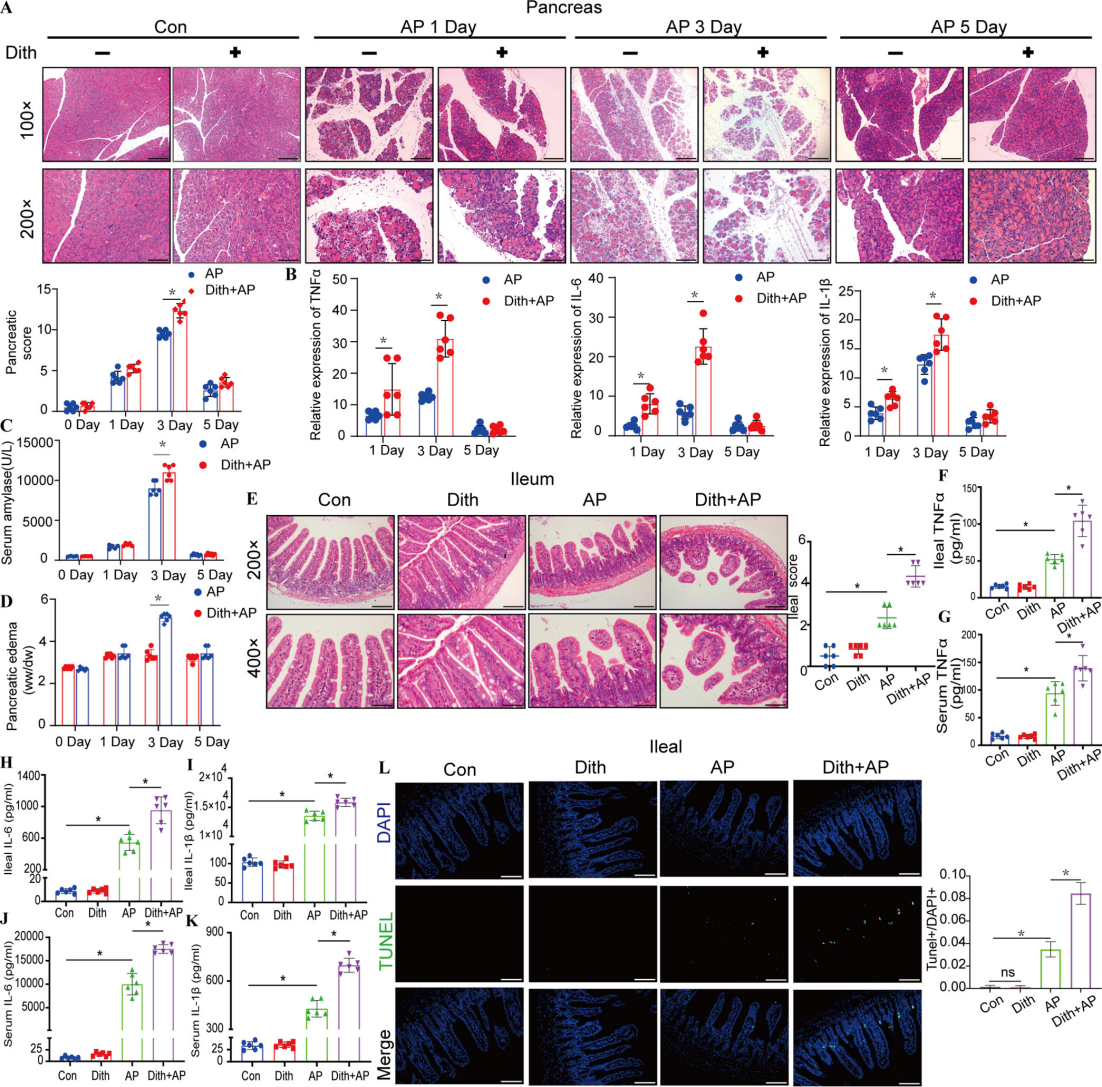
Long-term reduction of Paneth cells aggravated AP-induced pancreatic and ileal injuries and inflammation. (A) Pancreatic histopathological changes in AP mice with or without dithizone treatment. Original magnification, ×100 (top panels) or ×200 (bottom panels) (*n* = 6 mice per group). (B) mRNA expression levels of TNF-α, IL-6, and IL-1β. (C) Level of serum amylase. (D) Level of pancreatic edema. ww, wet weight; dw, dry weight. (E) Ileal histopathological changes. Original magnification, ×200 (top panels) or ×400 (bottom panels) (*n* = 6 mice per group). (F to K) Ileal and serum levels of TNF-α, IL-6, and IL-1β. (L) TUNEL staining of small intestines (magnification, ×200) and TUNEL^+^/DAPI^+^ quantification. The data are presented as the means ± SD. ns, no significant difference; *, *P ≤ *0.05.

In our previous study, rats were treated intraperitoneally with 100 mg/kg of body weight of dithizone (Dith) to ablate Paneth cells for 48 h (14). In this study, by increasing the frequency of injection and adjusting the dose of dithizone, the number of Paneth cells in mouse intestinal crypts and the expression of lysozyme and Defa5 decreased to approximately one-half of the original levels for 2 weeks (Fig. S2A to E). AP was induced by l-arginine on the basis of the long-term reduction of Paneth cells (Fig. S2A). Pancreatic pathological injuries and inflammation, amylase levels, and the pancreatic wet weight-to-dry weight (W/D) ratios of AP mice treated with Dith (Dith+AP mice) reached the highest levels 3 days following AP induction and were more severe (*P < *0.05) than those of AP mice (Fig. 2A to D). Therefore, we chose 3 days as the optimal time for subsequent experiments.

10.1128/msystems.01507-21.2 FIG S2 Establishment of long-term reduction of Paneth cells. (A) Experimental design. Black arrows represent dithizone treatments, and red arrows represent the induction of AP. (B) Representative ileal immunofluorescence photographs of lysozyme (green) at different time points (magnification, ×200). Lysozyme-positive/DAPI^+^ cells were also measured at different time points. (C) Mean number of Paneth cells per crypt at different time points. (D and E) mRNA expression levels of Defa5 (D) and lysozyme (E) at different time points. The data are presented as the means ± SD. *, *P ≤ *0.05. Download FIG S2, TIF file, 2.5 MB. Copyright © 2022 Fu et al. 2022 Fu et al. https://creativecommons.org/licenses/by/4.0/ This content is distributed under the terms of the Creative Commons Attribution 4.0 International license.

**FIG 3:**
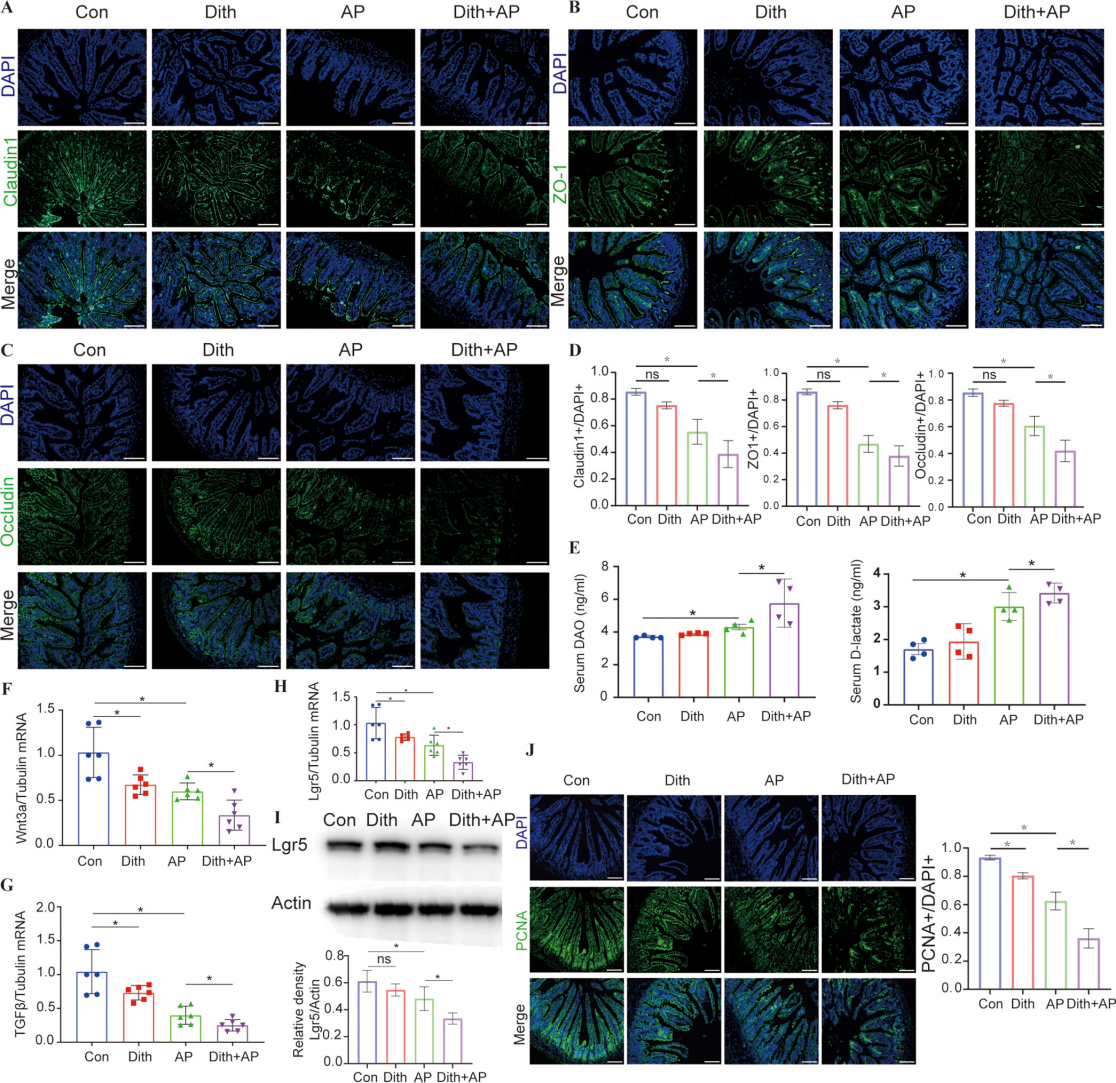
Long-term reduction of Paneth cells increased intestinal permeability. (A to D) Images of ileal claudin1 (A), ZO-1 (B), and occludin (C) immunofluorescence (magnification, ×200) and corresponding cellular quantification (D) (*n* = 6 mice per group). (E) Levels of serum DAO and d-lactate. (F to H) mRNA expression levels of Wnt3a (F), TGFβ (G), and Lgr5 (H). (I) Expression and quantification of intestinal Lgr5. (J) Immunofluorescence staining and quantification of PCNA (magnification, ×200). The data are presented as the means ± SD. ns, no significant difference; *, *P ≤ *0.05.

Dith+AP mice also exhibited more severe ileal pathological injuries (*P < *0.05) and more robust increases (*P < *0.05) in serum and ileal proinflammatory cytokines (tumor necrosis factor alpha [TNF-α], interleukin-6 [IL-6], and IL-1β) than did AP mice (Fig. 2E to K). Terminal deoxynucleotidyltransferase-mediated dUTP-biotin nick end labeling (TUNEL) staining showed that the long-term reduction of Paneth cells caused more ileal apoptosis (*P < *0.05) in Dith+AP mice (Fig. 2L). Compared with AP mice, the expression of tight junction proteins (TJPs) (claudin1, occludin, and ZO-1) decreased in Dith+AP mice (Fig. 3A to D). Serum diamine oxidase (DAO) and d-lactate levels were also higher in Dith+AP mice than in AP mice (*P < *0.05), suggesting increased intestinal permeability (Fig. 3E).

It has been proven that Paneth cells constituted the niche for Lgr5-positive (Lgr5^+^) stem cells in intestinal crypts by secreting Wnt3a and TGFβ, etc. (10). Compared with AP mice, the expression levels of Wnt3a, TGFβ, and Lgr5 were significantly downregulated (*P < *0.05) in Dith+AP mice (Fig. 3F to I). Proliferating cell nuclear antigen (PCNA) staining showed that the reduction of Paneth cells inhibited the proliferation of intestinal epithelial cells (IECs) in Dith+AP mice (Fig. 3J).

**FIG 4:**
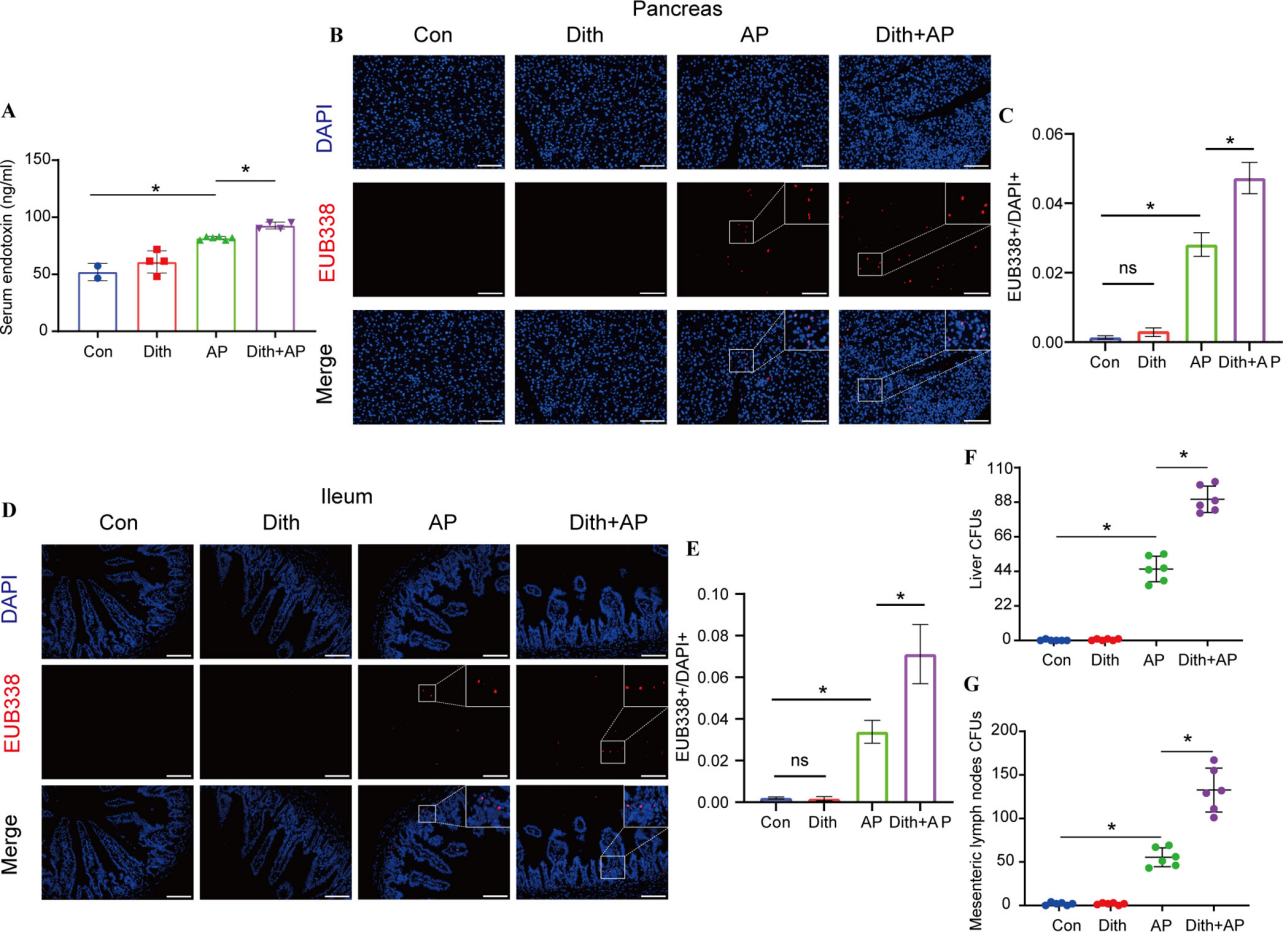
Long-term reduction of Paneth cells aggravated AP-induced bacterial translocation. (A) Levels of serum endotoxin. (B to E) Representative fluorescence photographs of the pancreas (magnification, ×100) and ileum (magnification, ×200) and EUB338^+^/DAPI^+^ quantification (*n* = 6 mice per group). (F and G) CFU were counted on anaerobic culture plates of liver (F) and mesenteric lymph nodes (MLN) (G). The data are presented as the means ± SD. ns, no significant difference; *, *P ≤ *0.05.

The progression of AP involves an increase in bacterial translocation caused by the disrupted intestinal barriers. Dith+AP mice harbored a higher level of endotoxin (*P < *0.05) than did AP mice (Fig. 4A). Compared with AP mice, no bacterial translocation was found in Dith mice, while Dith+AP mice presented increased bacterial translocation to the intestinal mucosa and pancreas using fluorescence *in situ* hybridization (FISH) analysis (Fig. 4B to E). The numbers of anaerobic bacteria in the liver and mesenteric lymph nodes (MLN) were counted on a brain heart infusion agar (BHIA) plate. Compared with AP mice, Dith+AP mice had more CFU (*P < *0.05), indicating that the liver and mesenteric lymph nodes of Dith+AP mice had more severe bacterial translocation (Fig. 4F and G).

### The aggravation of AP in Dith mice was related to the disturbance of the intestinal microbiota.

**FIG 5:**
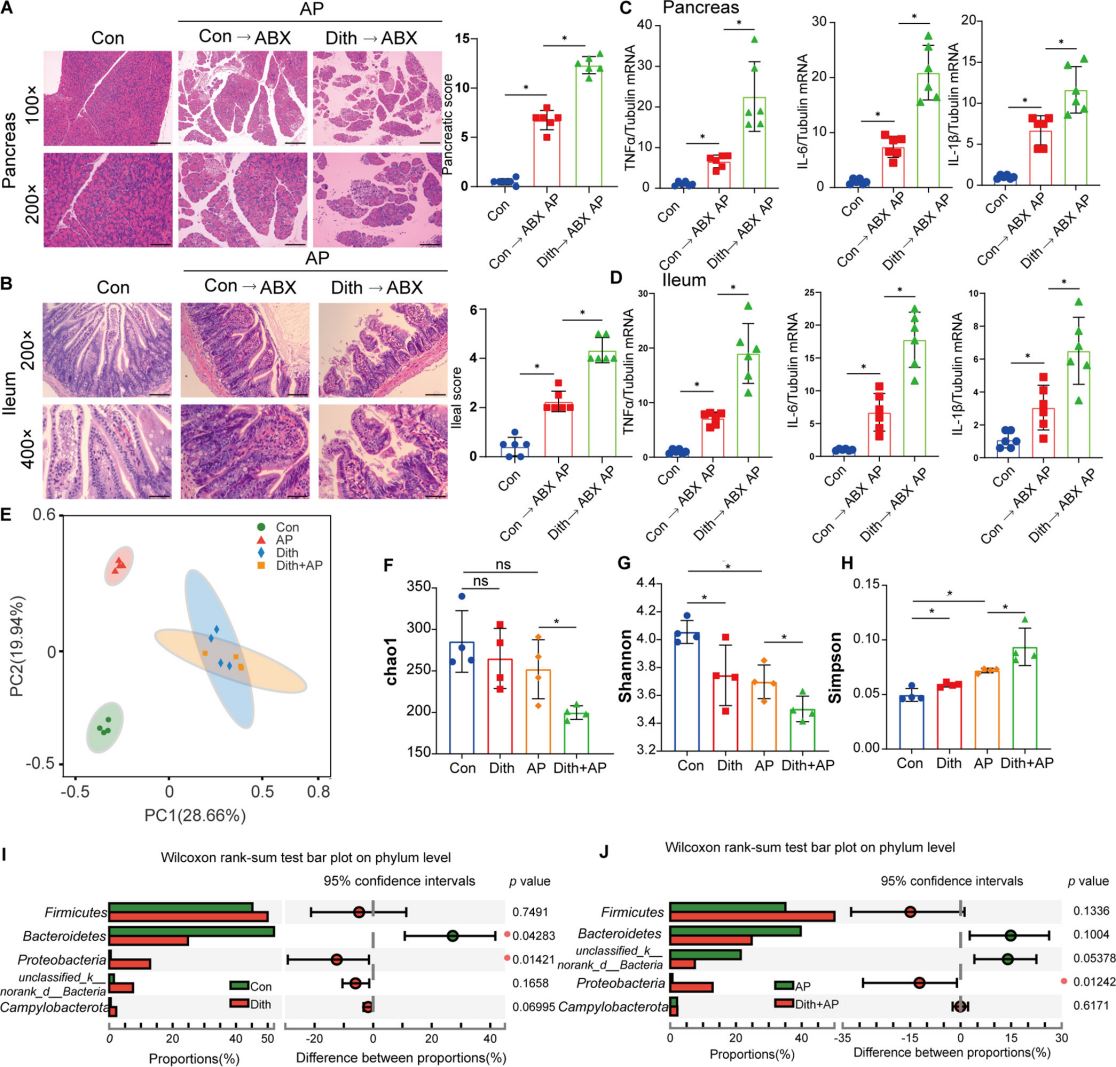
The disturbance of the intestinal microbiota was related to the exacerbation of AP. (A and B) Representative images of histopathological changes in the pancreas (magnification, ×100 [top panels] or ×200 [bottom panels]) (A) and ileum (magnification, ×200 [top panels] or ×400 [bottom panels]) (B) of ABX mice receiving FMT (*n* = 6 mice per group). (C and D) mRNA expression levels of TNF-α, IL-6, and IL-1β in the pancreas (C) and ileum (D). (E) Principal-coordinate analysis (PCoA) (*n* = 4 to 6 mice per group) of the microbial communities. (F to H) Alpha diversity, as revealed by the Chao1 index (F), the Shannon index (G), and the Simpson index (H), was analyzed in Con and Dith mice with or without AP. (I and J) Relative abundances of the top five phyla in Con and Dith mice with or without AP. The data are presented as the means ± SD. ns, no significant difference; *, *P ≤ *0.05.

To explore the role of the gut microbiota in exacerbated injuries and inflammation in Dith+AP mice, we transplanted the fecal microbiota of Dith mice and Con mice to antibiotic-treated mice (ABX mice), followed by the induction of AP. Compared with those in ABX mice receiving feces from Con mice, pathological damage and the mRNA expression levels of TNF-α, IL-6, and IL-1β were increased significantly (*P < *0.05) in both the pancreatic and ileal tissues of ABX mice receiving feces from Dith mice (Fig. 5A to D).

We then analyzed the cecal contents by 16S rRNA sequencing. Principal-coordinate analysis (PCoA) showed that the intestinal microbiota of AP mice was largely separated from that of Con mice. Simultaneously, different bacterial communities were revealed between Con mice and Dith mice. There was a partial overlap between bacterial communities in Dith and Dith+AP mice (Fig. 5E). Compared with Con mice, the alpha diversity of bacterial communities was greatly decreased in Dith mice, as reflected by the decreased Shannon index (*P < *0.05) and the increased Simpson index (*P < *0.05) (Fig. 5F to H). Compared with AP mice, the alpha diversity was also markedly decreased in Dith+AP mice, as evidenced by the decreased Shannon index and Chao1 index and the increased Simpson index (Fig. 5F to H).

*Firmicutes* and *Bacteroidetes* are two dominant bacteria at the phylum level (17). An increase in the relative abundance of *Firmicutes* and a decrease in the relative abundance of *Bacteroidetes* resulted in an increase in the *Firmicutes/Bacteroidetes* (F/B) ratio (*P* < 0.05) in Dith mice and Dith+AP mice (Fig. 5I and J; Fig. S3A). The relative abundance of *Proteobacteria*, which include Escherichia*-Shigella*, *Helicobacter*, and other pathogenic bacteria (18), also tended to increase in Dith mice and Dith+AP mice (*P < *0.05) (Fig. 5I and J).

10.1128/msystems.01507-21.3 FIG S3 (A) *Firmicutes/Bacteroidetes* (F/B) ratios in AP mice and Dith mice with or without AP. (B) F/B ratios in Dith+AP mice and Lyz+Dith+AP mice. (C) Levels of common species of *Helicobacter* and *Blautia*. The data are presented as the means ± SD. ns, no significant difference; *, *P ≤ *0.05. Download FIG S3, TIF file, 0.7 MB. Copyright © 2022 Fu et al. 2022 Fu et al. https://creativecommons.org/licenses/by/4.0/ This content is distributed under the terms of the Creative Commons Attribution 4.0 International license.

**FIG 6:**
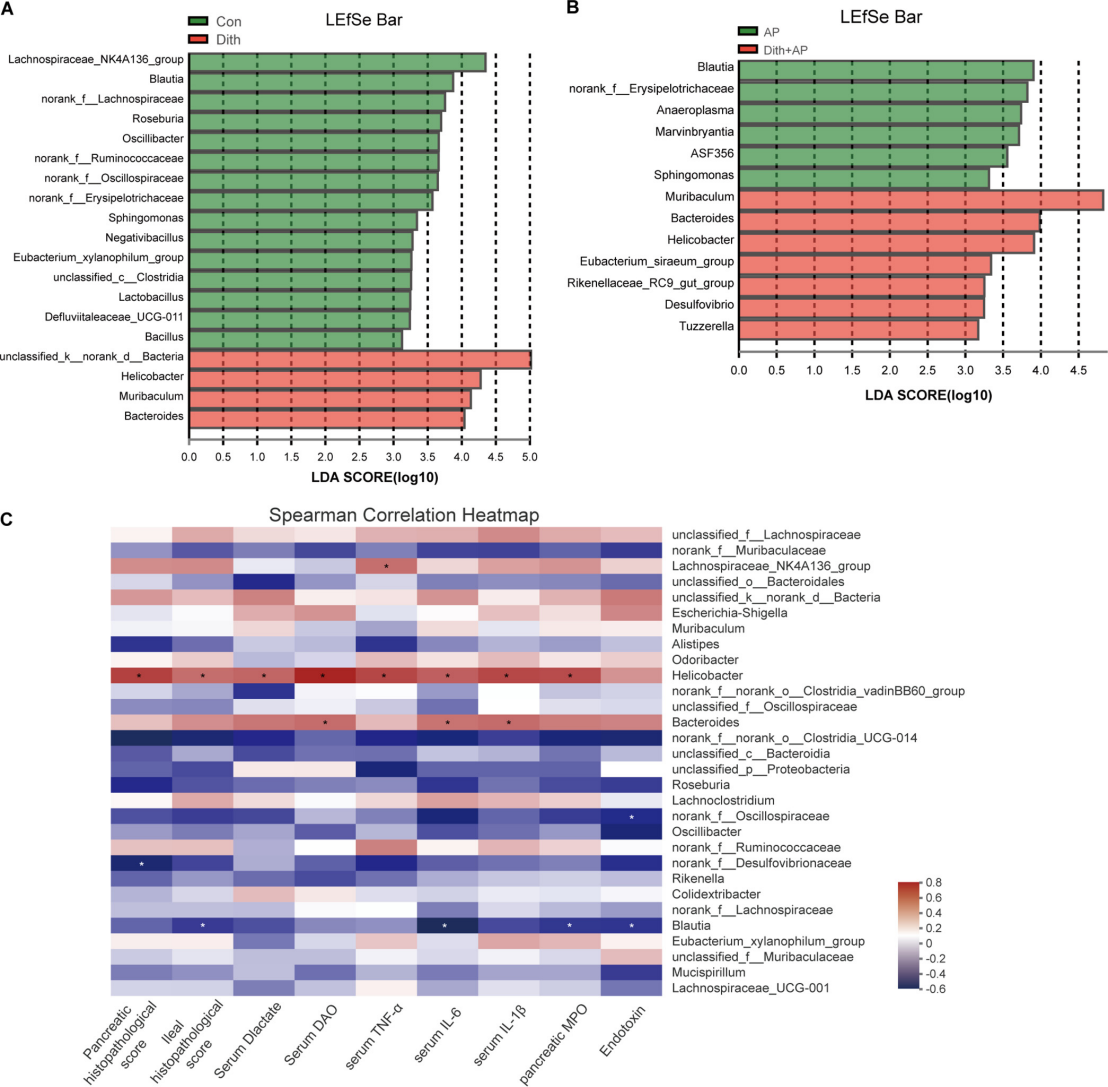
Changes in the gut microbiota at the genus level. (A) Linear discriminant analysis (LDA) scores at the genus level between Con and Dith mice. (B) LDA scores at the genus level between AP and Dith+AP mice. (C) Heatmap showing a correlation among intestinal barrier dysfunction, pathological changes, inflammation cytokines, and the gut microbiota. Blue, negative correlation; red, positive correlation. The data are presented as the means ± SD. ns, no significant difference; *, *P ≤ *0.05.

Next, we performed linear discriminant analysis (LDA) coupled with effect size (LEfSe) on the gut microbiota between Con mice and Dith mice with or without AP. At the genus level, the relative abundances of *Bacteroides* and *Helicobacter* increased significantly (*P < *0.05) while the relative abundance of *Blautia* decreased markedly (*P < *0.05) in Dith mice and Dith+AP mice (Fig. 6A and B). Spearman’s correlation analysis showed that the relative abundance of *Helicobacter* is positively correlated with the pancreatic and ileal histopathological scores and the levels of serum DAO and d-lactate, serum proinflammatory cytokines, and pancreatic myeloperoxidase (MPO). In contrast, the relative abundance of *Blautia* was negatively associated with the ileal histopathological score, serum IL-6, pancreatic MPO, and endotoxin (Fig. 6C).

### Lysozyme ameliorated AP-induced injuries and inflammation in Dith mice.

The functional recovery of Paneth cells has been reported to effectively correct intestinal dysbiosis (19). We next evaluated the therapeutic potential of AMPs of Paneth cells in AP mice. Compared with AP mice, the mRNA expression levels of lysozyme (Lyz), Defa5, Reg3γ, Reg3β, Ang4, and cryptdin1 in Dith+AP mice decreased significantly (*P < *0.05), while the mRNA expression levels of matrix metalloproteinase 7 (MMP7) and sPLA2 were not significantly altered (Fig. S4A). The protein expression levels of lysozyme, Defa5, and Ang4 were downregulated in Dith+AP mice (Fig. S4B and Fig. S6A). Spearman’s correlation analysis revealed that the severity of AP was inversely associated with the levels of Paneth cell AMPs, where lysozyme harbored the highest correlation coefficient (Fig. S4C). Therefore, we chose supplementation with lysozyme for the functional recovery of Paneth cells to restore intestinal homeostasis.

10.1128/msystems.01507-21.4 FIG S4 (A and B) In Dith mice with or without AP, the mRNA expression levels of ileal lysozyme, Defa5, Reg3γ, MMP7, Reg3β, Ang4, sPLA2, and cryptdin1 were assessed by real-time PCR (A), and the protein expression levels of ileal lysozyme, sPLA2, Reg3γ, Ang4, and Defa5 were assessed by Western blotting (B). (C) Heatmap showing a correlation between the relative expression of AMPs and the levels of proinflammatory factors. Blue, negative correlation; red, positive correlation. (D to G) Immunofluorescence staining of claudin1 (D), ZO-1 (E), and occludin (F) (green) (magnification, ×200) and quantification of fluorescence in Dith+AP mice with or without AP (G). The data are presented as the means ± SD. ns, no significant difference; *, *P ≤ *0.05. Download FIG S4, TIF file, 2.5 MB. Copyright © 2022 Fu et al. 2022 Fu et al. https://creativecommons.org/licenses/by/4.0/ This content is distributed under the terms of the Creative Commons Attribution 4.0 International license.

10.1128/msystems.01507-21.6 FIG S6 The protein expression levels of lysozyme, Reg3γ, sPLA2, Ang4, and Defa5 (A) and Lgr5, Wnt3a, β-catenin, c-myc, occludin, and claudin1 (B) were evaluated. The data are presented as the means ± SD. ns, no significant difference; *, *P ≤ *0.05. Download FIG S6, TIF file, 1.2 MB. Copyright © 2022 Fu et al. 2022 Fu et al. https://creativecommons.org/licenses/by/4.0/ This content is distributed under the terms of the Creative Commons Attribution 4.0 International license.

**FIG 7:**
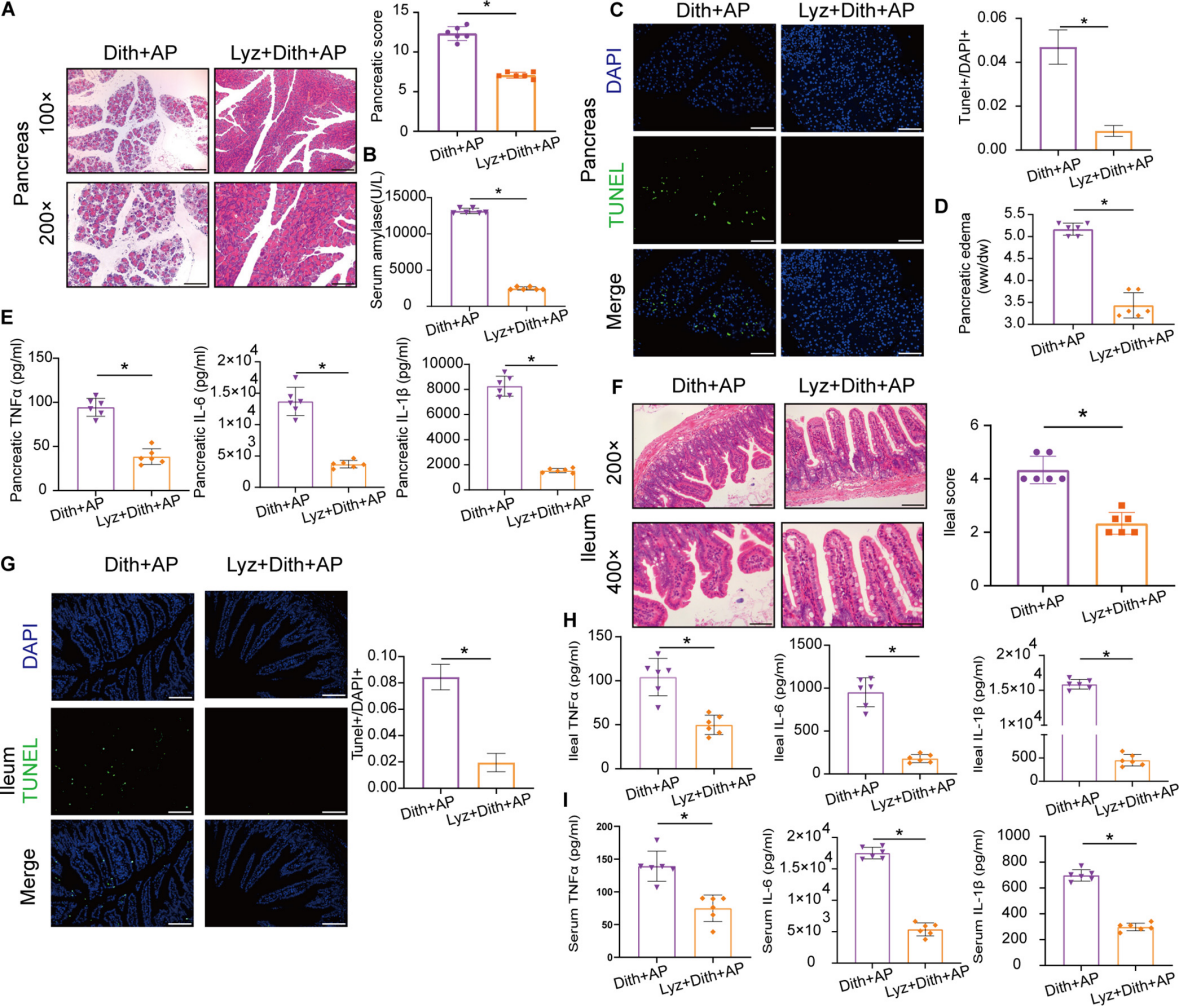
Lysozyme ameliorated the severity of AP and prevented bacterial translocation. (A) Representative pancreatic sections after H&E staining. Original magnification, ×100 (top panels) or ×200 (bottom panels) (*n* = 6 mice per group). (B) Level of serum amylase. (C) TUNEL staining and TUNEL^+^/DAPI^+^ quantification of apoptosis in the pancreas (magnification, ×100). (D) Level of pancreatic edema. (E) Levels of TNF-α, IL-6, and IL-1β in the pancreas. (F) Representative ileal sections after H&E staining. Original magnification, ×200 (top panels) or ×400 (bottom panels). (G) TUNEL staining and TUNEL^+^/DAPI^+^ quantification of apoptosis in small intestines (magnification, ×200). (H) Ileal and serum levels of TNF-α, IL-6, and IL-1β. The data are presented as the means ± SD. ns, no significant difference; *, *P ≤ *0.05.

We observed less severe pancreatic injuries and apoptosis as well as much lower amylase levels and pancreatic W/D ratios in Lyz+Dith+AP mice than in Dith+AP mice (*P < *0.05) (Fig. 7A to D). Pancreatic inflammation was attenuated in Lyz+Dith+AP mice (*P < *0.05), as evidenced by the decline in proinflammatory cytokines (Fig. 7E). Compared with Dith+AP mice, Lyz+Dith+AP mice also showed mild intestinal epithelial injuries and apoptosis along with decreased ileal and systematic inflammation (*P < *0.05) (Fig. 7F to H).

### Lysozyme restored intestinal barrier integrity and protected against bacterial translocation.

**FIG 8:**
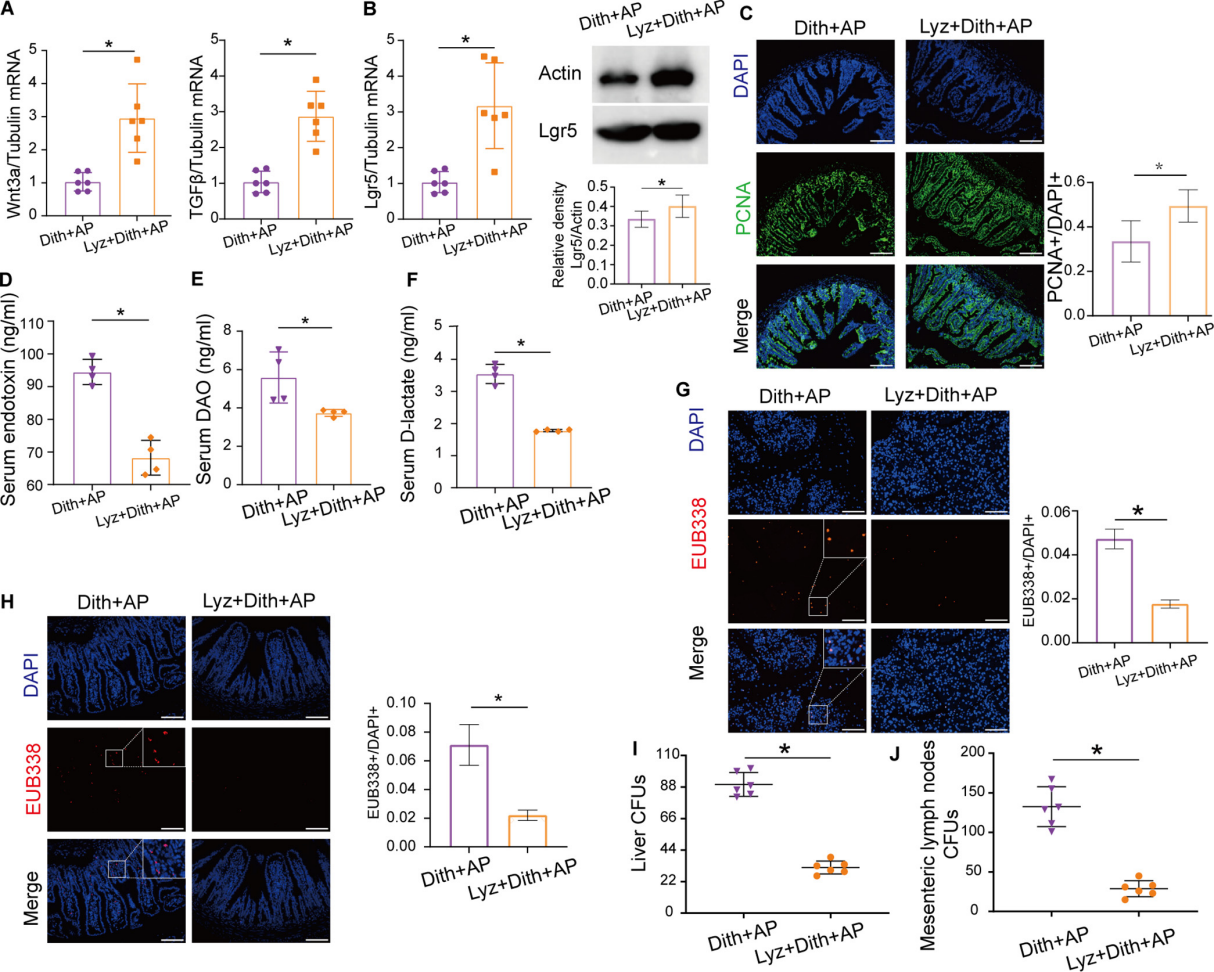
Lysozyme prevented bacterial translocation. (A) mRNA expression of TGFβ and Wnt3a. (B) mRNA and protein expression of Lgr5. (C) Immunofluorescence staining and quantification of PCNA (magnification, ×200). (D to F) Levels of serum endotoxin (D), DAO (E), and d-lactate (F). (G and H) FISH tests of the pancreas (magnification, ×100) (G) and intestinal epithelium (magnification, ×200) (H) using the EUB338 probe. EUB338^+^/DAPI^+^ cells were quantified. (I and J) CFU were counted on anaerobic culture plates of liver (I) and MLN (J). The data are presented as the means ± SD. ns, no significant difference; *, *P ≤ *0.05.

The expression of the TJPs (claudin1, occludin, and ZO-1) was increased following lysozyme administration in Dith+AP mice (Fig. S4D to G). The mRNA expression of Wnt3a, TGFβ, and Lgr5 and the protein expression of Lgr5 were restored in Lyz+Dith+AP mice (Fig. 8A and B). Moreover, lysozyme supplementation restored the proliferation of IECs, which was suppressed in Dith+AP mice, as determined by PCNA staining (Fig. 8C).

Lyz+Dith+AP mice presented lower intestinal permeability than Dith+AP mice, based on measurements of serum levels of d-lactate, DAO, and endotoxin (*P < *0.05) (Fig. 8D to F). The FISH analysis confirmed decreased numbers of bacteria within the intestinal mucosa and the pancreas of Lyz+Dith+AP mice versus Dith+AP mice (Fig. 8G and H). The numbers of anaerobes translocated to the liver and mesenteric lymph nodes were also reduced significantly in Lyz+Dith+AP mice (*P < *0.05) (Fig. 8I and J). These results suggested that pretreatment with lysozyme attenuated AP by reducing bacterial translocation and promoted mucosal repair by stimulating the proliferation of IECs.

### Lysozyme regulated microbiota disorders induced by dysfunction of Paneth cells.

**FIG 9:**
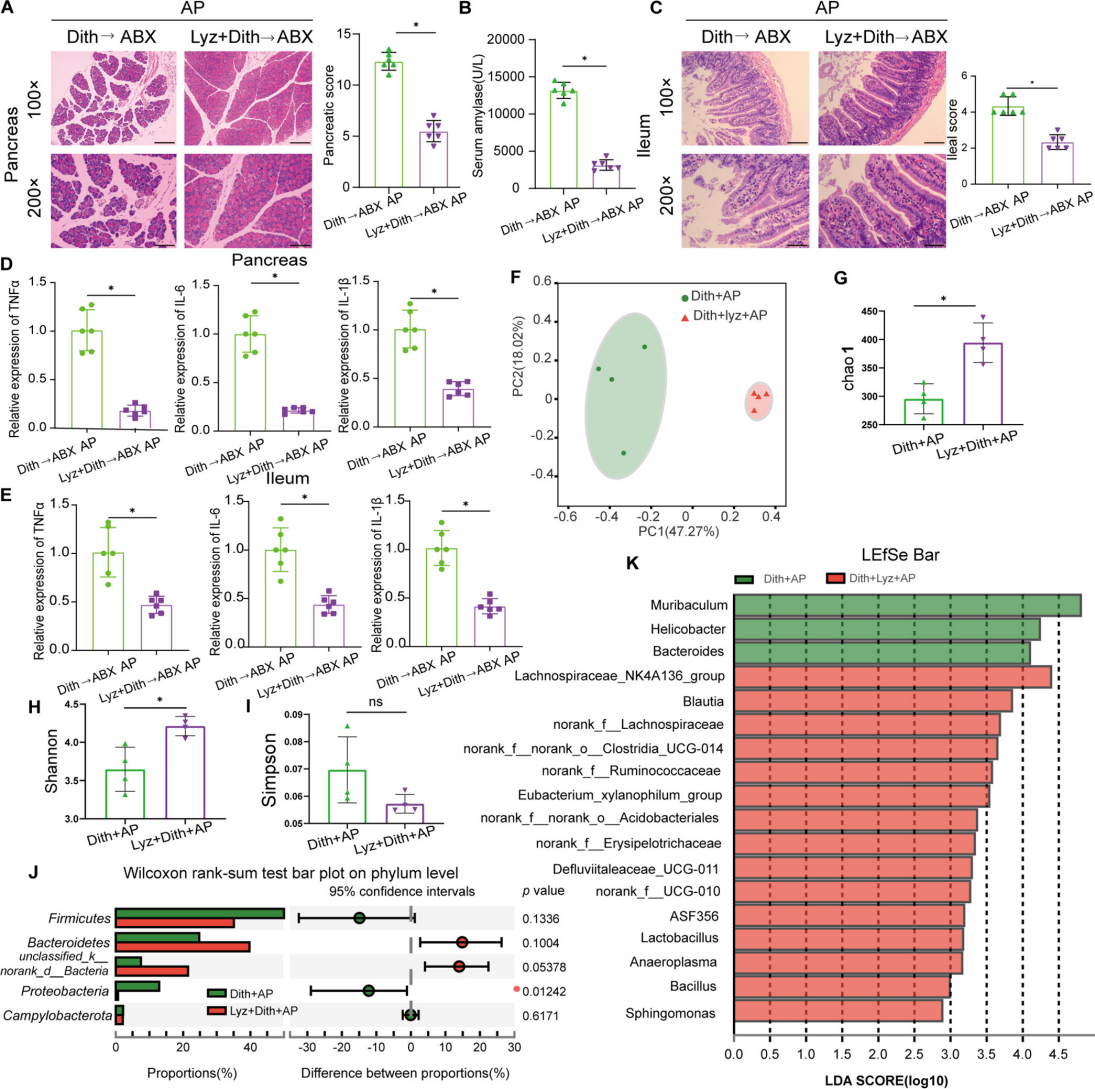
Lysozyme restored microbiota disorders induced by dysfunction of Paneth cells. (A) Representative photographs of H&E staining of the pancreas of ABX mice receiving FMT (*n* = 6 mice per group). (B) Level of amylase. (C) Representative photographs of H&E staining of the ileum of ABX mice receiving FMT (*n* = 6 mice per group). (D and E) mRNA expression levels of TNF-α, IL-6, and IL-1β in the pancreas (D) and ileum (E). (F) Principal-coordinate analysis (PCoA) of bacterial beta diversity (*n* = 4 to 6 mice per group). (G to I) Alpha diversity analysis using the Chao1 index, the Shannon index, and the Simpson index. (J) Relative abundances of the top five phyla in Dith+AP mice and Lyz+Dith+AP mice. (K) LDA scores at the genus level between Dith+AP mice and Lyz+Dith+AP mice. The data are presented as the means ± SD. ns, no significant difference; *, *P ≤ *0.05.

We then evaluated the contribution of the lysozyme-modulated microbiota in Dith+AP mice by fecal microbiota transplantation (FMT). The fecal microbiota of Lyz+Dith mice and Dith mice was transplanted to ABX mice, followed by the induction of AP. ABX mice receiving feces from Lyz+Dith mice developed less severe pancreatic and ileal injuries (*P < *0.05) than those receiving feces from Dith mice (Fig. 9A to C). Alleviation of pancreatic and ileal inflammation was evidenced by reduced proinflammatory cytokines (*P < *0.05) by real-time PCR in ABX mice receiving FMT from Lyz+Dith mice compared with those receiving FMT from Dith mice (Fig. 9D and E). Therefore, lysozyme markedly reduced the severity of AP exacerbated in Dith+AP mice via regulating the gut microbiota.

PCoA showed that lysozyme gavage greatly shifted the microbiota structure in Dith+AP mice (Fig. 9F). Compared with Dith+AP mice, the increased Chao1 index and Shannon index (*P < *0.05) and decreased Simpson index (*P < *0.05) showed increased the alpha diversity of bacterial communities in Lyz+Dith+AP mice (Fig. 9G to I). At the phylum level, compared with Dith+AP mice, Lyz+Dith+AP mice presented an increased relative abundance of *Firmicutes* (*P < *0.05), decreased relative abundances of *Bacteroidetes* and *Proteobacteria* (*P < *0.05), and a normalized F/B ratio (*P < *0.05) (Fig. 9J; Fig. S3B). At the genus level, supplementation with lysozyme restored the relative abundances of *Helicobacter* and *Blautia* in Dith+AP mice (*P < *0.05) (Fig. 9K). Therefore, lysozyme could restructure the microbiota composition disrupted in Dith+AP mice.

We then carried out real-time PCR for four prevalent species of *Helicobacter* and three prevalent species of *Blautia* (20, 21) (Fig. S3C). Compared with the Con group, the levels of Helicobacter felis, Helicobacter hepaticus, and Blautia coccoides increased significantly and the level of Blautia obeum decreased greatly in the Dith group, and the levels of Helicobacter bilis, H. hepaticus, and Blautia wexlerae increased significantly and the level of *B. obeum* decreased greatly in the AP group. Compared with AP group, the levels of H. bilis, H. felis, B. coccoides, B. obeum, and B. wexlerae decreased greatly and the level of H. hepaticus increased significantly in the Dith+AP group. Compared with the Dith+AP group, the levels of *H. felis*, *B. obeum*, and B. wexlerae increased greatly and the levels of H. hepaticus and *B. coccoides* decreased significantly in the Lyz+Dith+AP group. Although an association of Helicobacter pylori with prolonged hospital stay in AP patients has been reported previously (22), H. pylori cannot be detected by real-time PCR in the contents of the cecum (data not shown) in our research. The possible significance of specific species is discussed in Discussion.

### Lysozyme promoted enteroid proliferation by regulating the functions of Paneth cells.

**FIG 10:**
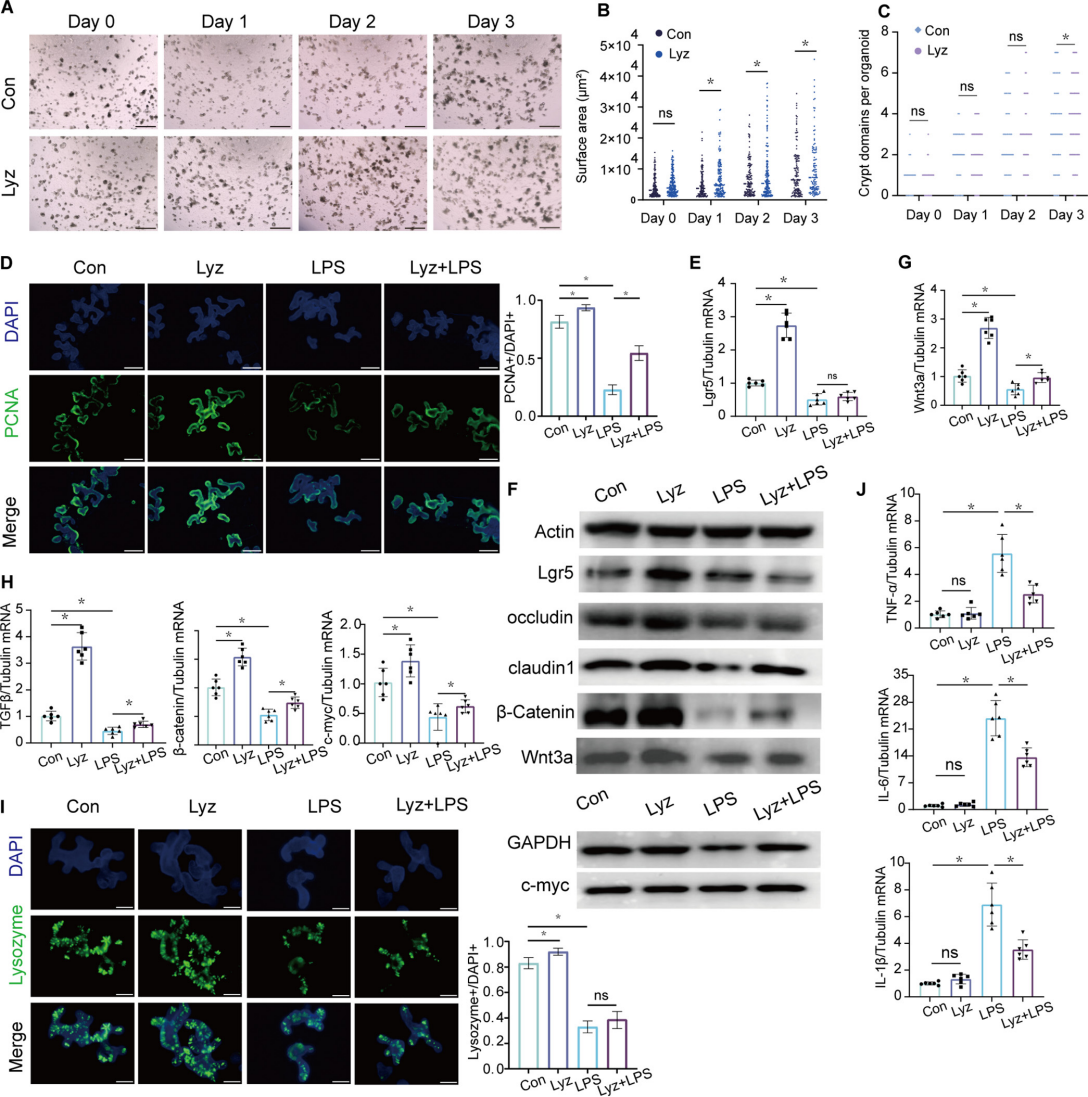
Lysozyme promoted enteroid proliferation. (A) Light microscopy photographs showing normal morphology and sizes of enteroids in the Lyz group compared to the Con group. (B) The mean surface area of enteroids was estimated 1 day, 2 days, and 3 days after incubation with lysozyme. (C) Crypt domains per enteroid were counted. (D) Representative images of fluorescence staining of PCNA (green) of enteroids after incubation with lysozyme and LPS. The expression of PCNA was quantified. (E) mRNA expression of Lgr5. (F) Image of Western blotting and protein quantification of Lgr5, occludin, claudin1, β-catenin, Wnt3a, and c-*myc*. GAPDH, glyceraldehyde-3-phosphate dehydrogenase. (G and H) mRNA expression levels of Wnt3a (G) and TGFβ, β-catenin, and c-*myc* (H). (I) Representative images of fluorescence staining of lysozyme (green) of enteroids. The expression of lysozyme was quantified. (J) mRNA expression levels of TNF-α, IL-6, and IL-1β of enteroids after incubation with lysozyme and LPS. The data are presented as the means ± SD. ns, no significant difference; *, *P ≤ *0.05.

We cultured enteroids to investigate IEC-lysozyme interactions based on the well-established technology of three-dimensional (3D) culture. Compared with the Con group, we observed that after 72 h of intervention with lysozyme, both the surface area and the number of crypt buds per enteroid increased significantly (*P < *0.05) (Fig. 10A to C) (23). Lysozyme administration also promoted the proliferation of enteroids, as reflected by increases in the expression of PCNA, TJPs (occludin and claudin1), and Lgr5 (*P < *0.05) (Fig. 10D to F; Fig. S6B). Compared with the Con group, Wnt3a and TGFβ, along with β-catenin and c-*myc*, two crucial molecules of the Wnt signaling pathway (24, 25), were significantly upregulated (*P < *0.05) in the Lyz group (Fig. 10F to H; Fig. S6B). In addition, the mRNA expression of AMPs (lysozyme, Defa5, Reg3γ, and Ang4) was also increased greatly (*P < *0.05) in the Lyz group (Fig. 10I; Fig. S5A to D).

LPS was utilized to imitate the inflammatory microenvironment in the enteroid system (26). In the Lyz+LPS group, the release of proinflammatory factors was decreased (*P < *0.05) (Fig. 10J), while cell proliferation and the integrity of the intercellular TJPs, which were disrupted in the LPS group, were protected by lysozyme (Fig. 10D to H; Fig. S6B). Note that lysozyme failed to reverse the reduction of AMPs in Paneth cells induced by LPS (Fig. 10I; Fig. S5A to D).

10.1128/msystems.01507-21.5 FIG S5 The mRNA expression levels of lysozyme (A), Defa5 (B), Reg3γ (C), and Ang4 (D) were determined to evaluate intestinal permeability. The data are presented as the means ± SD. ns, no significant difference; *, *P ≤ *0.05. Download FIG S5, TIF file, 0.4 MB. Copyright © 2022 Fu et al. 2022 Fu et al. https://creativecommons.org/licenses/by/4.0/ This content is distributed under the terms of the Creative Commons Attribution 4.0 International license.

## DISCUSSION

In this study, we first confirmed the dysfunction of Paneth cells in AP patients. Our *in vivo* experiments showed that the long-term reduction of Paneth cells exacerbated injuries and inflammation in the pancreas and small intestine in AP mice. Dith+AP mice also presented with increased intestinal permeability, bacterial translocation, and intestinal microbiota disorder compared with AP mice. Such changes were significantly reversed by lysozyme treatment. The functional recovery of Paneth cells might be a novel target for the treatment of intestinal dysfunction during AP.

Paneth cells constitute a part of innate immunity by secreting various antimicrobial peptides. A high concentration of antimicrobial peptides in crypts constructs a relatively sterile environment and prevents pathogen invasion (27). The involvement of Paneth cell dysfunction in the pathogenesis of multiple diseases has been widely reported, such as in Crohn’s disease (CD) (11), alcoholic steatohepatitis (28), graft-versus-host disease (GVHD) (29), and irritable bowel syndrome (IBS) (15), etc. The dysfunction of Paneth cells in AP patients and AP mice, manifested by the marked reduction in the number of Paneth cells and the expression of AMPs, was in line with previous findings in rats (30). Our previous study demonstrated that acute ablation of Paneth cells using dithizone aggravated the severity of rat AP, but the mechanism of the protective role of Paneth cells in AP remains unclear (14). Therefore, we established a model with a long-term reduction of Paneth cells. Inflammation and damage were significantly increased in the pancreas and small intestine of Dith+AP mice compared with those of AP mice.

AP is often accompanied by intestinal barrier dysfunction, and the translocation of bacteria derived from the small intestine exacerbates systemic inflammation (31). Intestinal dysfunction in AP is thought to be associated with ischemia-reperfusion damage, severe oxidative stress, and apoptosis in the intestinal mucosa (32, 33). In recent years, Paneth cells have been proven to regulate the proliferation of intestinal stem cells and maintain the dynamic balance of intestinal epithelial cells by secreting support factors (10). Bifidobacterium longum promotes cell proliferation and the expression of Lgr5 and Wnt3a in intestinal organoids by regulating the functions of Paneth cells (34). VDR^ΔPC^ mice exhibited abnormal Paneth cells and decreased expression of PCNA and β-catenin (35). Our study found that compared with AP mice, Dith+AP mice exhibited markedly decreased TJP expression and increased intestinal permeability and bacterial translocation. Meanwhile, the expression of Lgr5, Wnt3a, and TGFβ and intestinal epithelial proliferation were also significantly reduced. These findings suggested that Paneth cell dysfunction resulting in its diminished support for intestinal stem cells was one of the reasons for increased intestinal permeability during AP.

Intestinal microbiota disorders are common in patients with moderate or severe AP and are significantly related to the severity of inflammation, indicating that the intestinal microbiota is involved in the progression of AP (5). The administration of Escherichia coli MG1655 to AP rats aggravated injuries in the pancreas and small intestine and activated Toll-like receptor 4 (TLR4)/MyD88/mitogen-activated protein kinase (MAPK) and endoplasmic reticulum stress in intestinal epithelial cells, while *Parabacteroides* produces acetate to alleviate heparanase-exacerbated AP by reducing neutrophil infiltration (36, 37). The significantly reduced inflammation in ABX mice with AP further proved the role of the gut microbiota in AP (36, 38). Paneth cells protect the host from intestinal pathogens and shape the composition of the colonized microbiota. FMT proved that gut microbiota disturbance caused by the long-term reduction of Paneth cells played an important role in AP aggravation. 16S rRNA sequencing of cecal contents revealed that the long-term reduction of Paneth cells altered the gut microbiota structure, decreased richness and diversity, increased the relative abundance of the deleterious bacterium *Helicobacter*, and decreased the relative abundance of the beneficial bacterium *Blautia*.

Supplementing products of Paneth cells as functional recovery is a commonly used method in studies related to Paneth cell dysfunction. HD5 supplementation effectively altered the gut microbiota in alcoholic hepatitis and reversed alcohol-induced damage (28). Lysozyme supplementation prevented Escherichia coli expansion and visceral hypersensitivity during maternal isolation (15). ANG1 treatment prevented dysbiosis in mice and alleviated dextran sulfate sodium (DSS)-induced colitis (39). The overexpression of Reg3γ protected mice from alcoholic hepatitis and reduced bacterial translocation (40). Supplementation of lysozyme was most appropriate for the functional recovery of Paneth cells in our study. Pretreatment with lysozyme restored gut microbiota disturbance, reduced the relative abundance of *Helicobacter*, restored the relative abundance of *Blautia*, and reversed aggravated ileal and pancreatic injuries in Dith+AP mice. FMT further confirmed that Paneth cells played a protective role in AP by stabilizing the intestinal microbiota.

Lysozyme is a cornerstone of innate immunity. Previous *in vitro* studies showed that lysozyme is a nonspecific antimicrobial peptide, while different bacteria possessed distinct sensitivities to lysozyme (41–44). Although in our research, H. pylori cannot be detected by real-time PCR, studies have shown that H. pylori-positive patients exhibited a higher relative abundance of *Proteobacteria* (45). The changes in H. hepaticus and *B. obeum* are of concern and warrant further research (see Fig. S3C in the supplemental material). Previous studies demonstrated that cytolethal distending toxin subunit B (CdtB) produced by H. hepaticus exacerbated the severity of colitis via inducing an inflammatory response and activating the Jak-Stat signaling pathway (46, 47). *B. obeum* generating bile salt hydrolases (BSHs) could inhibit the growth and colonization of Vibrio cholerae and Clostridioides difficile (48, 49). These studies suggested that changes of H. hepaticus and *B. obeum* might be involved in the progression of AP. The role of species altered by Paneth cell depletion or lysozyme supplementation in AP requires further investigation.

In addition to the antimicrobial effect, lysozyme modulated innate immunity. The sensing of the lysozyme-mediated production of pathogen-associated molecular patterns (PAMPs) by pattern recognition receptors (PRRs) stimulated downstream proinflammatory signaling and the production of proinflammatory cytokines (50, 51). Lysozyme could also limit intestinal inflammation. Zhang et al. showed that intestinal inflammation was associated with a failure to secrete Paneth cell lysozyme in a mouse model of Crohn’s disease (52, 53). Furthermore, lysozyme supplementation could ameliorate intestinal inflammation in porcine colitis (54). The mechanisms of lysozyme limiting intestinal inflammation were still unclear, with speculation of limited bacterial invasion and activated protective intestinal immune responses.

Organoid techniques have become a powerful tool for studying the intestinal epithelium *in vitro* (55, 56). Lysozyme intervention promoted the growth of organoids, activated the Wnt pathway, and promoted epithelial proliferation. Therefore, lysozyme secreted by Paneth cells not only maintains microbiota homeostasis but also promotes the proliferation of the intestinal organoids. Further studies designed to explore possible mechanisms should be performed.

We first used the method of multiple intraperitoneal injections of dithizone to maintain Paneth cells at a low level, but this method still had limitations. The protective effect of the functional recovery of Paneth cells in AP mice provides new strategies for the clinical treatment of intestinal complications during AP.

## MATERIALS AND METHODS

### Human intestinal biopsy specimens.

After obtaining written informed consent, human intestinal biopsy specimens from the descending part of the duodenum of 21 patients with AP and 14 healthy controls were obtained upon endoscopy from the Department of Gastroenterology of Shanghai General Hospital, excluding individuals with diseases affecting Paneth cells, including irritable bowel syndrome, inflammatory bowel disease, and alcoholic liver disease, etc. There was no statistical difference in baseline demographic and clinical characteristics between AP patients and healthy controls (see Table S1 in the supplemental material). Biopsy specimens were stored in 4% paraformaldehyde or liquid nitrogen.

### Animals.

Male C57BL/6 mice (6 to 8 weeks of age, 20 to 25 g) were obtained from Shanghai SLAC Laboratory Animal Co. Mice were housed under specific-pathogen-free (SPF) conditions with a room temperature of 24°C ± 2°C and a 12-h light/dark cycle.

### Experimental design.

Mice were randomly divided into 5 groups (*n* = 6): a control (Con) group, a dithizone (Dith) group, an AP group, a Dith+AP group, and a lysozyme (Lyz)-treated (Lyz+Dith+AP) group. Mice in the Dith group, the Dith+AP group, and the Lyz+Dith+AP group were intraperitoneally injected with 40 mg/kg dithizone (Sigma-Aldrich, USA) every 3 days for 2 weeks. The mice in the Lyz+Dith+AP group received an oral gavage of 200 U/day lysozyme (Sigma-Aldrich, USA) for 2 weeks. Mice in the Con group and the AP group were intraperitoneally injected with normal saline (NS). After treatment with dithizone or NS, mice in the AP group, the Dith+AP group, and the Lyz+Dith+AP group were injected intraperitoneally twice with 4.5 g/kg l-arginine (Sigma-Aldrich, USA). Mice in the Con group and the Dith group were intraperitoneally injected with NS. Mice in the Cae+AP group were injected intraperitoneally with 100 μg/kg cerulein (MedChemExpress, China) 10 times, with an hour interval between consecutive injections. After the final injection, 5 mg/kg lipopolysaccharide (Sigma-Aldrich, USA) was intraperitoneally injected. AP was induced in mice in the N+AP group as previously described (57). A 2% sodium taurocholate (Sigma-Aldrich, USA) solution at a volume of 50 μL/20 g of body weight was infused into the biliopancreatic duct at a rate of 5 μL/min. Mice were anesthetized with tiletamine and zolazepam (Virbac, France) and then sacrificed 72 h after the first injection of l-arginine. Blood samples, distal ileum, pancreas, liver, and mesenteric lymph nodes were collected and stored at −80°C or in 4% paraformaldehyde. Fresh contents of the ileocecum were also collected for analysis of the gut microbiota.

### Histological analysis.

Fresh pancreas and distal ileum were soaked in 4% paraformaldehyde and dehydrated. Tissues were then embedded in paraffin and cut into sections of 4 μm. Sections were stained with hematoxylin and eosin (H&E; Servicebio, China) as previously described (58). Histopathological injuries were examined by using a light microscope (Leica, Germany). Pancreatic injury was assessed according to the scoring criteria reported previously by Shimizu et al. (59), while distal ileal injury was evaluated as described previously by Chiu et al. (60). Paneth cells were counted as previously reported (61).

### Real-time PCR.

Tissue total RNA was extracted using TRIzol (Invitrogen, USA) and tissue RNA purification kit plus (EZBioscience, USA). cDNA synthesis was performed using HyperScript III RT SuperMix (EnzyArtisan, China) for quantitative PCR (qPCR) with genomic DNA (gDNA) remover. Bacterial DNA was extracted from fecal samples with an E.Z.N.A. stool DNA kit (Omega, USA). The concentration of RNA or DNA was determined by using a NanoDrop2000 instrument (Thermo Scientific, USA). S6 universal SYBR qPCR mix (2×) (EnzyArtisan, China) was used to perform real-time PCR with QuantStudio 6 Flex real-time PCR systems (Thermo Scientific, USA) according to the following protocol: predenaturation (95°C for 30 s), 40 amplification cycles of denaturation (95°C for 10 s), and annealing and extension (60°C for 30 s). Gene expression was measured by the 2^−ΔΔ^*^CT^* method. Primers used for detection are provided in Table S2.

10.1128/msystems.01507-21.9 TABLE S2 Sequences of the primers used in this study. Download Table S2, DOCX file, 0.01 MB. Copyright © 2022 Fu et al. 2022 Fu et al. https://creativecommons.org/licenses/by/4.0/ This content is distributed under the terms of the Creative Commons Attribution 4.0 International license.

### Pancreas W/D ratio and serum amylase assays.

The pancreatic tissue was weighed and then incubated at 80°C for 48 h to obtain a constant weight as the dry weight. The ratio of the wet pancreas weight to the dry pancreas weight (W/D ratio) was calculated to evaluate tissue edema. The level of serum amylase was determined with amylase reagents using the Advia 2400 chemistry system (Siemens, German) according to the technicians’ instructions.

### Immunofluorescence.

Distal ileal sections were heated at 60°C for 1 h. Next, sections were put in Leica Autostainer XL (Leica, USA) (xylene for 40 min, 100% ethanol for 10 min, 95% ethanol for 10 min, 80% ethanol for 5 min, 70% ethanol for 5 min, and doubly distilled water for 3 min) to deparaffinize and rehydrate the samples. Antigens were retrieved using a citrate antigen retrieval solution (Sangon Biotech, China). After repeated washing in phosphate-buffered saline (PBS), a super pap pen (Sangon Biotech, China) was used to draw a circle around the tissue. Slides were blocked with immunostaining blocking buffer (Sangon Biotech, China) at room temperature for 1 h and incubated with primary antibodies against PCNA (catalog number A0264; Abclonal, China), occludin (catalog number A2601; Abclonal, China), claudin1 (catalog number ab211737; Abcam, USA), and ZO-1 (catalog number 13663; Cell Signaling Technology, USA) diluted with primary antibody dilution buffer (Sangon Biotech, China) at 4°C overnight. Slides were washed with PBS and incubated with Alexa Fluor 488 AffiniPure donkey anti-rabbit IgG (Yeason, China) for 1 h at room temperature. Next, the slides were washed with PBS and stained with dihydrochloride (Yeason, China) for 10 min. Images were captured with a fluorescence microscope (Leica, USA).

### Western blotting.

Distal ileum tissues were lysed in radioimmunoprecipitation assay (RIPA) lysis buffer (Epizyme Biotech, China) with 1% protease inhibitor (Epizyme Biotech, China) and fully ground using a high-throughput tissue grinder (Onebio Biotech, China). The suspension was left to settle on ice for 1 h and centrifuged at 10,000 × *g* for 10 min at 4°C. After taking the supernatant and mixing it with SDS loading buffer (Yeason, China), the mixed solution was heated at 100°C for 10 min. Ten microliters of the solution was loaded into a 10% SDS-PAGE gel produced by using a PAGE gel fast preparation kit (Epizyme Biotech, China) for electrophoresis. Next, proteins in the gel were transferred to a 0.2-μm polyvinylidene difluoride (PVDF) membrane (Millipore, USA). The membrane was blocked with 3% bovine serum albumin (BSA) for 1 h and incubated with primary antibodies, diluted in primary antibody dilution buffer (Epizyme Biotech, China), against Lgr5 (catalog number A10545; Abclonal, China), lysozyme (catalog number A0099; Dako, Denmark), Reg3γ (catalog number sc-377038; Santa Cruz Biotechnology, USA), Defa5 (catalog number A18208; Abclonal, China), Ang4 (catalog number sc-377497; Santa Cruz Biotechnology, USA), and sPLA2 (catalog number sc-58363; Santa Cruz Biotechnology, USA) overnight at 4°C. On the second day, the membrane was washed 3 times with Tris-buffered saline with Tween 20 (TBST) buffer and incubated with peroxidase-conjugated goat anti-rabbit IgG(H+L) (Yeason, China) for 60 min at room temperature. The membrane was washed 3 times with TBST again. Bands were visualized with a horseradish peroxidase (HRP) substrate peroxide solution (Millipore, USA) using an Amersham 600 imager (General Electric, USA).

### Enzyme-linked immunosorbent assay.

The levels of IL-1β, TNF-α, and IL-6 in the serum, pancreas, and ileum were determined by using a Luminex mouse discovery assay kit (R&D Systems, USA) according to the manufacturer’s instructions. The levels of pancreatic MPO, serum endotoxin, DAO, and d-lactate were measured using an MPO mouse enzyme-linked immunosorbent assay (ELISA) kit, an endotoxin mouse ELISA kit, a DAO mouse ELISA kit, and a d-lactate mouse ELISA kit (MultiSciences Biotech, China) according to the provided protocols.

### TUNEL and FISH assays.

Apoptosis was evaluated by a TUNEL assay using a fluorescein TUNEL cell apoptosis detection kit (Servicebio, China) according to the manufacturer’s instructions. Pancreatic and ileal TUNEL-positive cell counting was performed at a ×200 magnification. Fluorescence *in situ* hybridization (FISH) was used to detect bacterial translocation. In short, sections of the distal ileum and pancreas were heated for 60 min and dewaxed (twice for 10 min with 100% xylene and 5 min with 100% ethanol). Next, sections were incubated with specific probes (EUB338 [5′-Cy3-GCTGCCTCCCGTAGGAGT-3′]) in a wet box at 52°C for 18 h. The sections were then washed and stained with 4′,6-diamidino-2-phenylindole (DAPI). Images were captured with a fluorescence microscope (Leica, USA).

### Bacterial cultures and plate counting.

Mesenteric lymph nodes and liver tissues were collected in sterile PBS, fully ground using a high-throughput tissue grinder (Onebio Biotech, China), and plated onto brain heart infusion agar plates for a culture of anaerobic bacteria. The plates were incubated for 48 h at 37°C using Oxoid AnaeroGen 2.5 L and an Oxoid resazurin anaerobic indicator (Thermo Scientific, USA). The plates producing 25 to 250 CFU were counted.

### Fecal microbiota transplantation.

Feces were collected from Con mice, Dith mice, and Dith+Lyz mice. Processing of the fecal microbiota transplantation (FMT) suspension was done within 2 h. One hundred milligrams of feces was resuspended in 1 mL of saline and centrifuged for 5 min. The supernatant was used as the FMT suspension. The mice receiving antibiotics were treated with vancomycin (0.5 mg/mL), neomycin (1 mg/mL), ampicillin (1 mg/mL), and metronidazole (1 mg/mL) (Sangon Biotech, China) in their drinking water for 4 weeks. Mice were divided into four groups: the Con group, the ABX+Con group, the ABX+Dith group, and the ABX+Dith+Lyz group. The Con group received no treatment. The ABX+Con group was gavaged with feces from a control mouse for 1 week, the ABX+Dith group was gavaged with 200 μL of an FMT suspension from Dith mice for 1 week, and the ABX+Dith+Lyz group was gavaged with 200 μL of an FMT suspension from Dith+Lyz mice for 1 week. AP was induced in mice from the ABX+Con group, the ABX+Dith group, and the ABX+Dith+Lyz group.

### 16S rRNA sequencing.

Genomic DNA was extracted from the contents of the ileocecum using the E.Z.N.A. stool DNA kit (Omega, USA) according to the manufacturer’s instructions and amplified using forward primer 5′-TACGGRAGGCAGCAG-3′ and reverse primer 5′-AGGGTATCTAATCCT-3′. 16S rRNA high-throughput sequencing was performed on an Illumina HiSeq platform (Illumina, USA) according to standard protocols by Majorbio Bio-Pharm Technology.

### Microbiome analysis.

The sequences were filtered with fastp (0.19.6) and merged with FLASH (v1.2.11). Next, the high-quality sequences were denoised using the DADA2 plug-in in the QIIME2 (version 2020.2) pipeline with recommended parameters, which are called amplicon sequence variants (ASVs). Taxonomic assignment of ASVs was performed using the naive Bayes consensus taxonomy classifier implemented in QIIME2 and the SILVA 16S rRNA database (v138). Analysis of the gut microbiota was carried out using the Majorbio Cloud platform (https://cloud.majorbio.com). Alpha diversity indices, including Chao1 richness, the Shannon index, and the Simpson index, were calculated with Mothur v1.30.1. Principal-coordinate analysis (PCoA) based on Bray-Curtis dissimilarity using the Vegan v2.5-3 package was performed to analyze the microbial communities in different samples. The linear discriminant analysis (LDA) effect size (LEfSe) algorithm was performed to identify the significantly abundant genera of bacteria among the different groups (LDA score of >2; *P* < 0.05). Correlations among histopathological score, serum d-lactate and DAO, serum inflammation factors, endotoxin, and the relative abundances of different genera were calculated using Spearman’s analysis.

### Enteroid establishment and coculture with lysozyme.

Enteroids were obtained from C57BL/6 mice. The distal 10 cm of the small intestine was collected and flushed gently with ice-cold PBS for 5 min. Next, the intestine was cut open along the longitudinal axis and cut into 2-mm segments. Crypts were isolated from the segments by incubation in 2 mM EDTA for 30 min and then in 5 mM EDTA for 30 min. After 5 min of resting, the supernatant was removed. Crypts were resuspended in 15 mL Dulbecco’s modified Eagle’s medium (DMEM)–F-12 medium (Wisent, China) with repeated blowing. Next, the suspension was filtered through a 70-μm filter mesh (BD Biosciences, USA) and centrifuged at 300 × *g* for 5 min. After the supernatant was discarded, the pellet was mixed with Matrigel (Corning, USA) and DMEM–F-12 medium in a 1:1 ratio. Next, 50 μL of the suspension was plated into each well of prewarmed 24-well plates, and 700 μL of IntestiCult organoid growth medium (mouse) (Stemcell Technologies, Canada) was added per well. In the following culture, half of the medium was replaced every 3 days and passaged every 9 days. Lysozyme (200 U) was added to enteroids in each well, and the mixture was incubated at 37°C with 5% CO_2_ for 72 h. A total of 1 mg/mL LPS and 200 U lysozyme were added to enteroids in each well, and the mixture was incubated for 24 h. Total RNA was extracted using an EZ-press RNA purification kit (EZBioscience, USA). The methods for real-time PCR, Western blotting, and immunofluorescence analysis of enteroids are the same as the ones described above.

### Statistical analysis.

Data are exhibited in the form of means ± standard deviations (SD). Comparisons between two groups with a normal distribution were performed by a *t* test. Spearman’s rank correlation coefficient was used to determine correlations between bacterial genus and indicators. One-way analysis of variance (ANOVA) was performed for three or more groups. Differences in the male/female ratio and body mass index (BMI) between groups were tested by the chi-square test. All the statistical analyses were carried out in IBM SPSS Statistics 25. A *P* value of ≤0.05 suggested a statistically significant difference.

### Study approval.

All studies involving human samples were approved by the Ethics Committee of Shanghai General Hospital (2021035) and registered in the Chinese Clinical Trial Registry (identifier ChiCTR1800017214). All the animal experiments were approved by the Institutional Animal Care and Use Committee (IACUC) of Shanghai General Hospital (2020AW095) and conducted according to the instructions of the IACUC.

### Data availability.

The raw data for 16S rRNA sequencing in this study are available in the SRA database with BioProject accession number PRJNA774193.

10.1128/msystems.01507-21.7 FIG S7 Original blot images of all quantifications from Western blotting. (A) Lgr5; (B) Lgr5; (C) Lgr5, occludin, and claudin1; (D) β-catenin and Wnt3a; (E) c-myc; (F) Ang4 and Defa5; (G) lysozyme; (H) Reg3γ; (I) sPLA2. Download FIG S7, TIF file, 2.8 MB. Copyright © 2022 Fu et al. 2022 Fu et al. https://creativecommons.org/licenses/by/4.0/ This content is distributed under the terms of the Creative Commons Attribution 4.0 International license.

## Supplementary Material

Reviewer comments
