# Supplementary material for: Paneth Cells Protect against Acute Pancreatitis via Modulating Gut Microbiota Dysbiosis
Source: mSystems. 2022 May 2;7(3):e01507-21. doi: 10.1128/msystems.01507-21 (PMC9239092; doi:10.1128/msystems.01507-21)
Supplement: TABLE S1 [file msystems.01507-21-s0008.docx]

|  | Healthy | AP patients | | *p* value |
| --- | --- | --- | --- | --- |
|  |  | Early stage（＜72 h） | ＜1week |  |
| Patient (n) | 14 | 14 | 7 |  |
| Male (n[%]) | 7[50] | 6[42.9] | 2[28.6] | 0.31 |
| Age (years) | 50.71±15.53 | 55.03±14.79 | 60.57±10.47 | 0.34 |
| BMI (kg/m^2^) | 23.84±2.53 | 23.74±3.72 | 22.66±2.74 | 0.69 |
| APACHE II |  | 6.52±4.29 | 6.86±2.11 | 0.46 |
