# Supplementary figures and images for: Paneth Cells Protect against Acute Pancreatitis via Modulating Gut Microbiota Dysbiosis
Source: mSystems. 2022 May 2;7(3):e01507-21. doi: 10.1128/msystems.01507-21 (PMC9239092; doi:10.1128/msystems.01507-21)

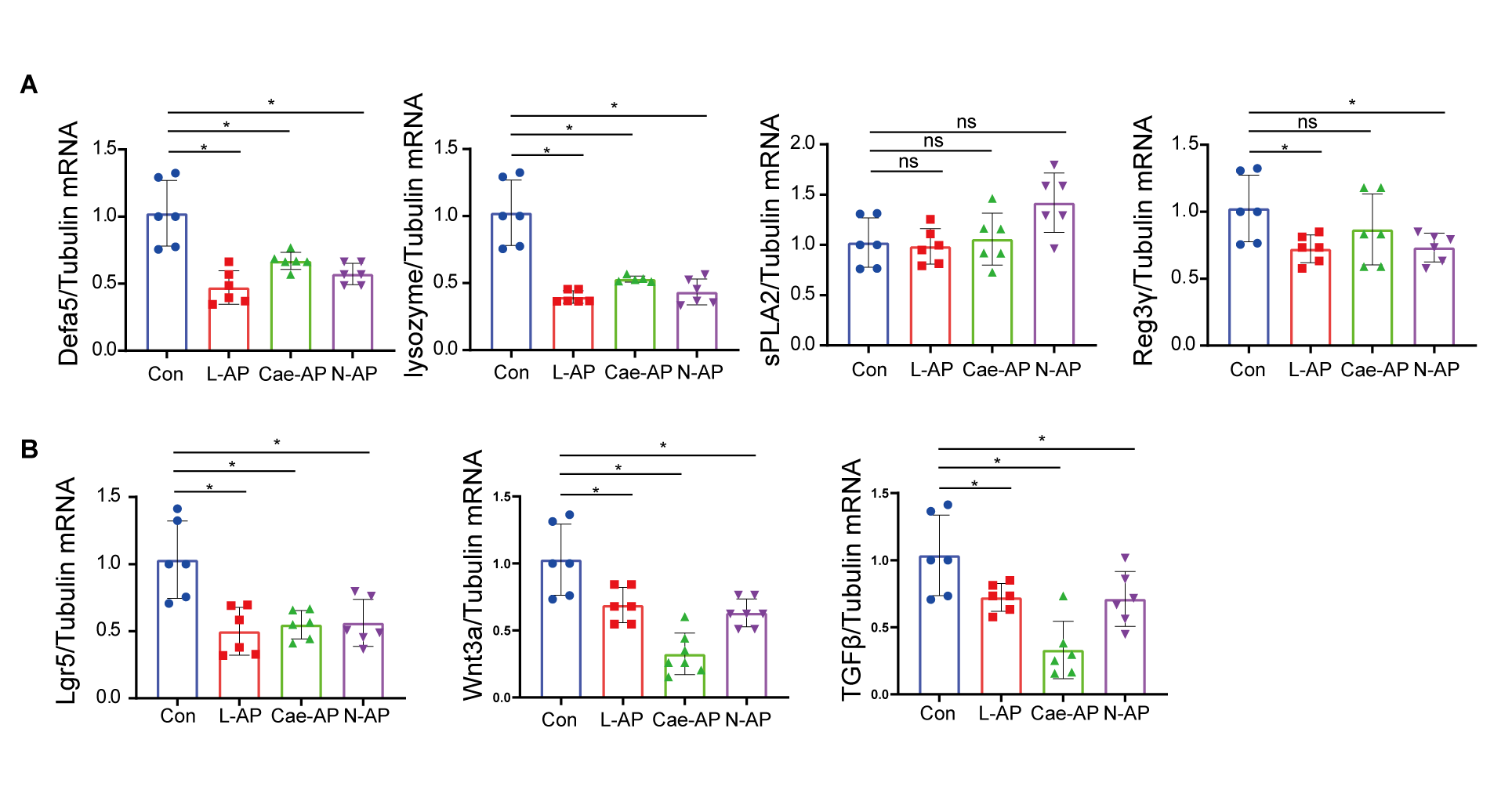

Supplement: FIG S1 [file msystems.01507-21-s0001.tif]

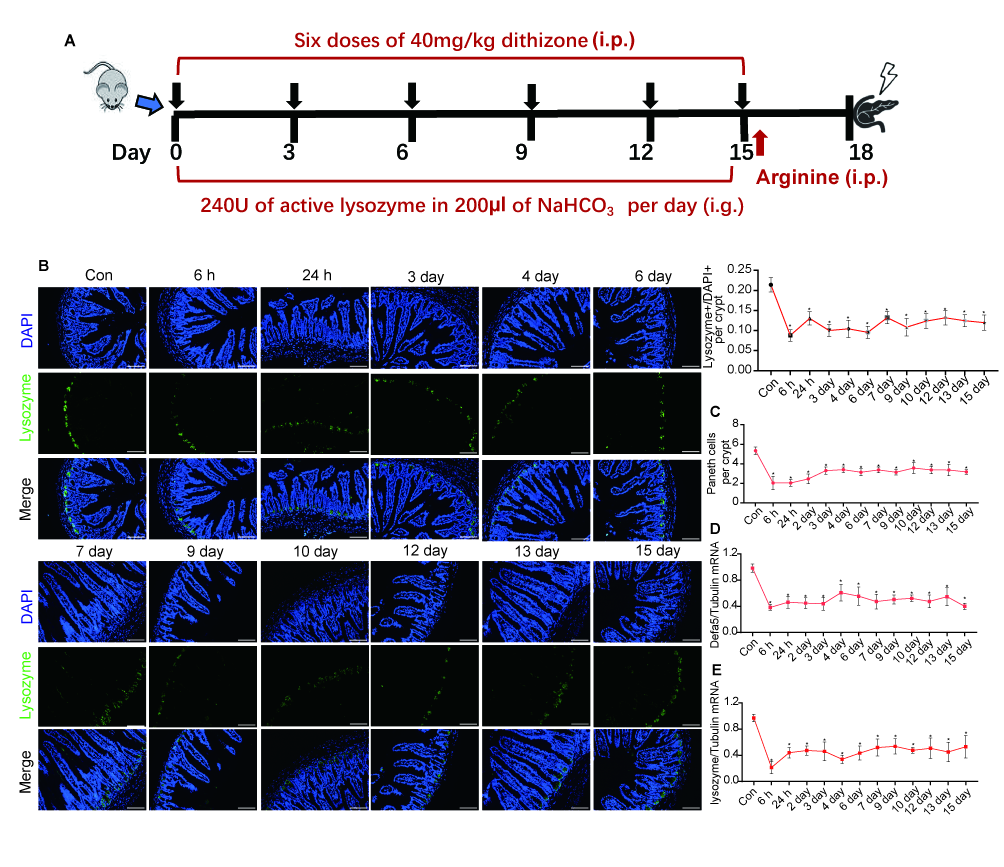

Supplement: FIG S2 [file msystems.01507-21-s0002.tif]

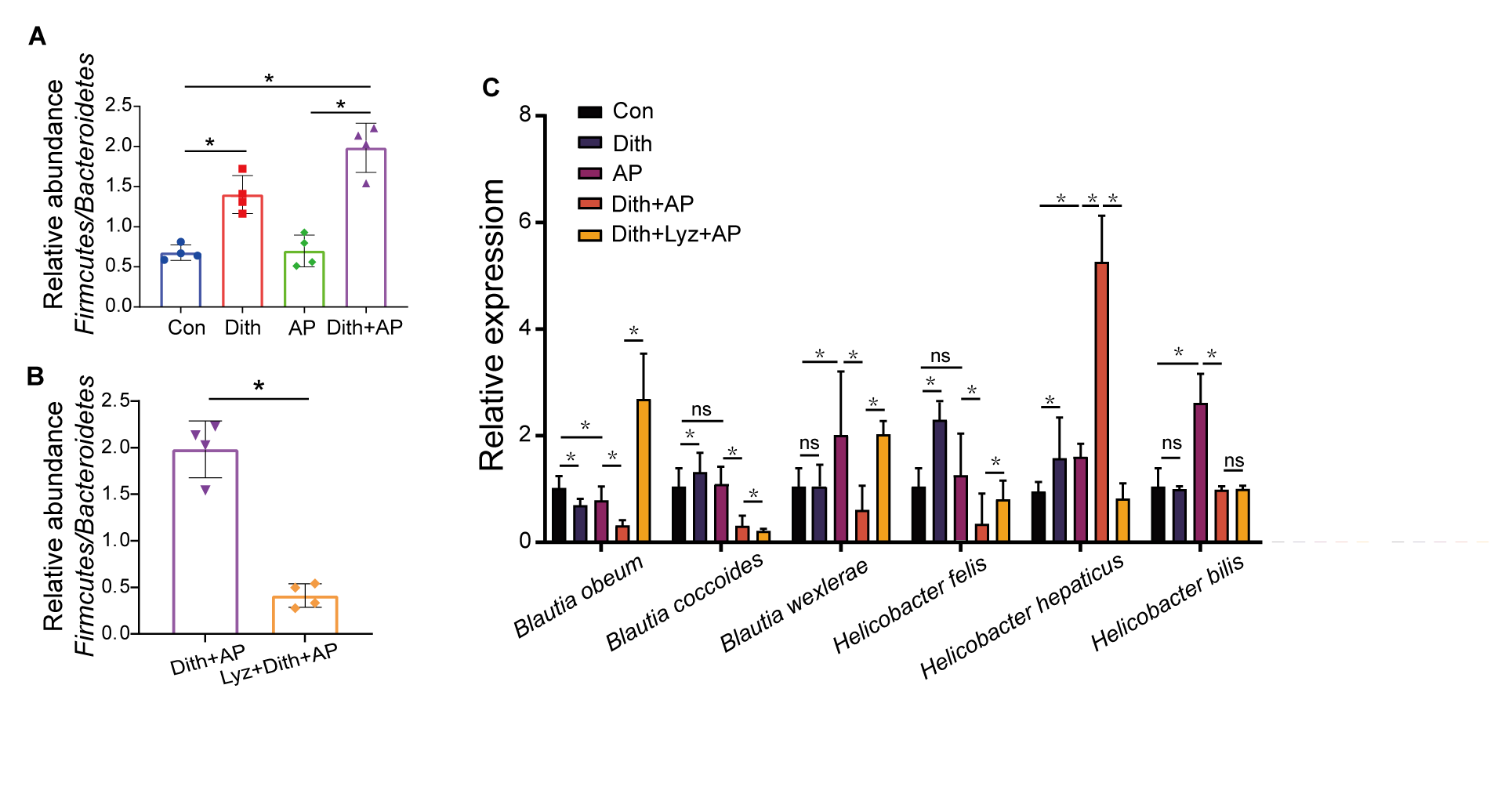

Supplement: FIG S3 [file msystems.01507-21-s0003.tif]

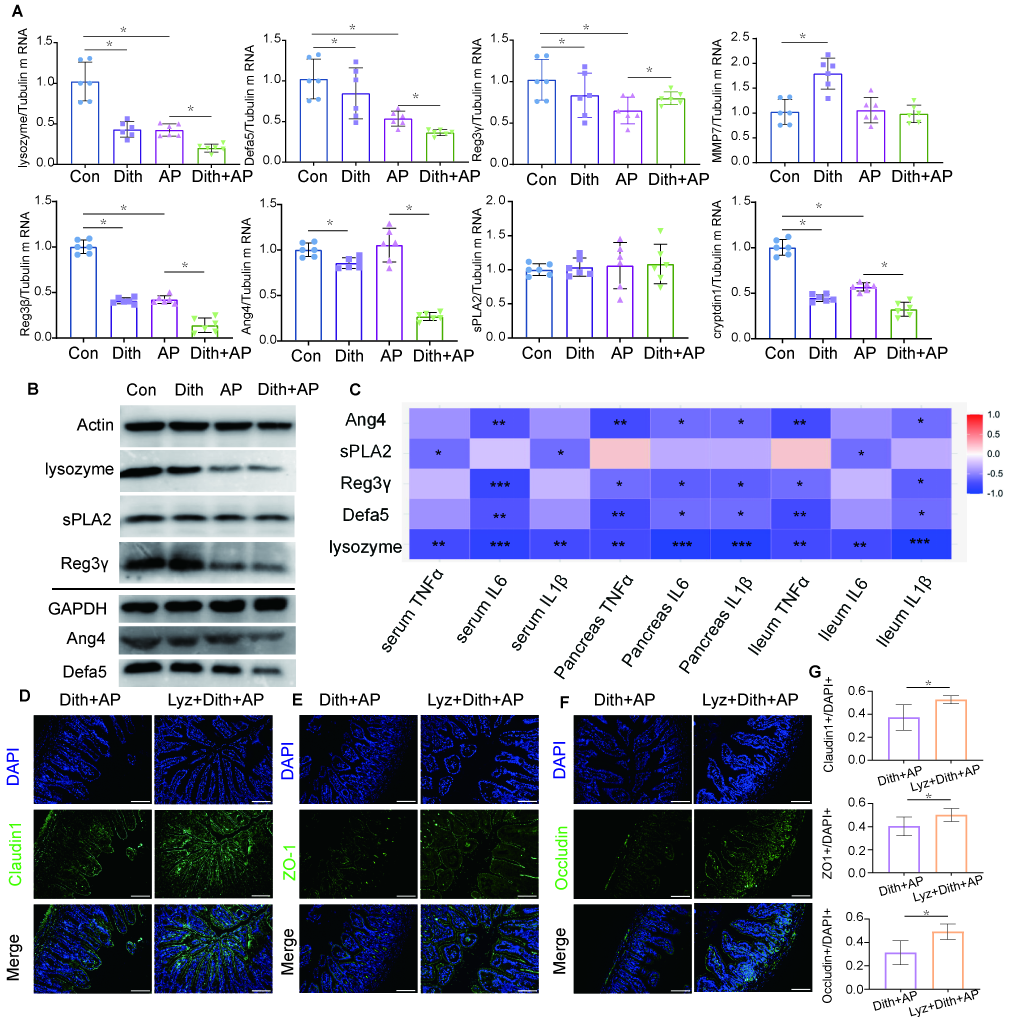

Supplement: FIG S4 [file msystems.01507-21-s0004.tif]

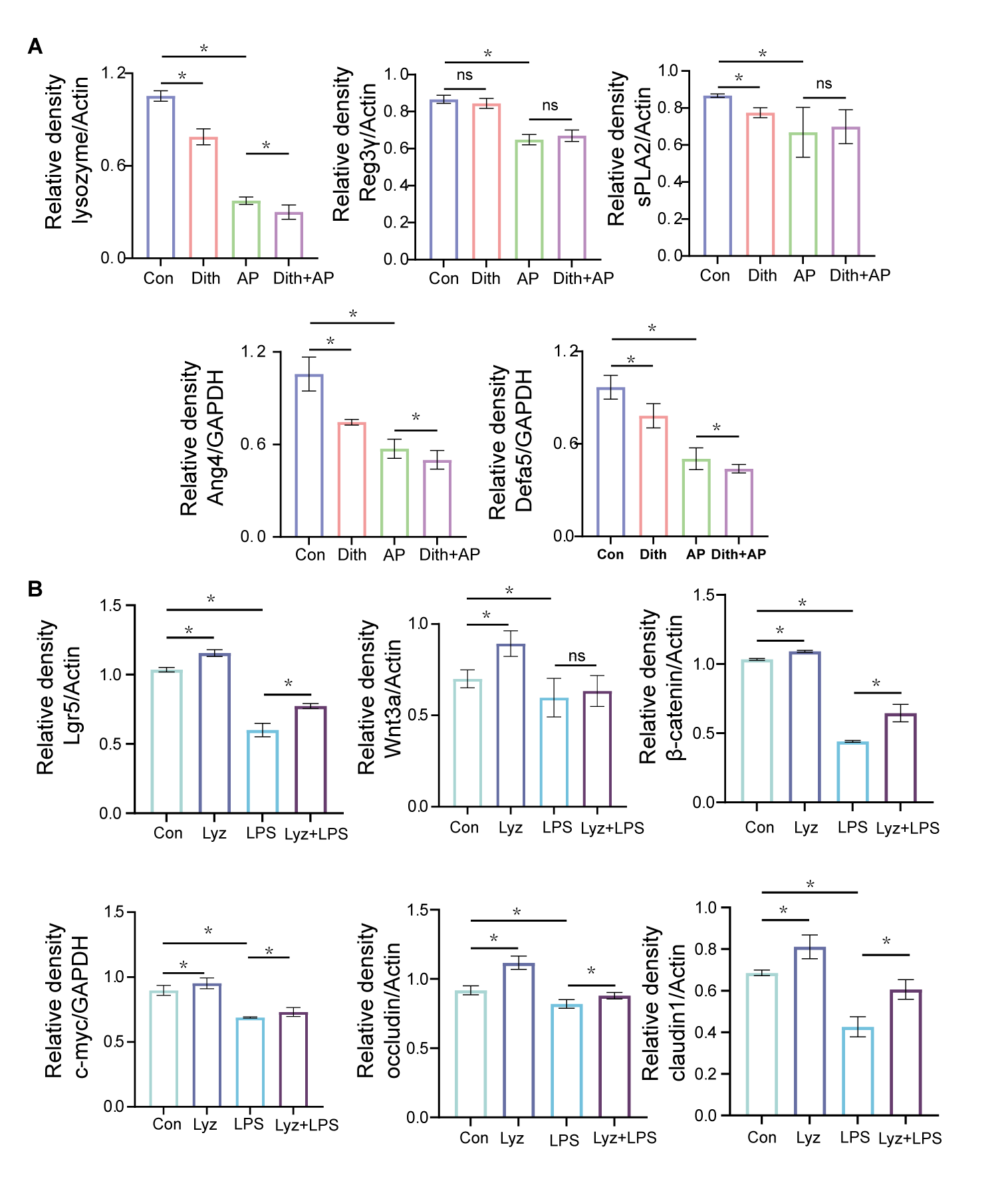

Supplement: FIG S6 [file msystems.01507-21-s0006.tif]

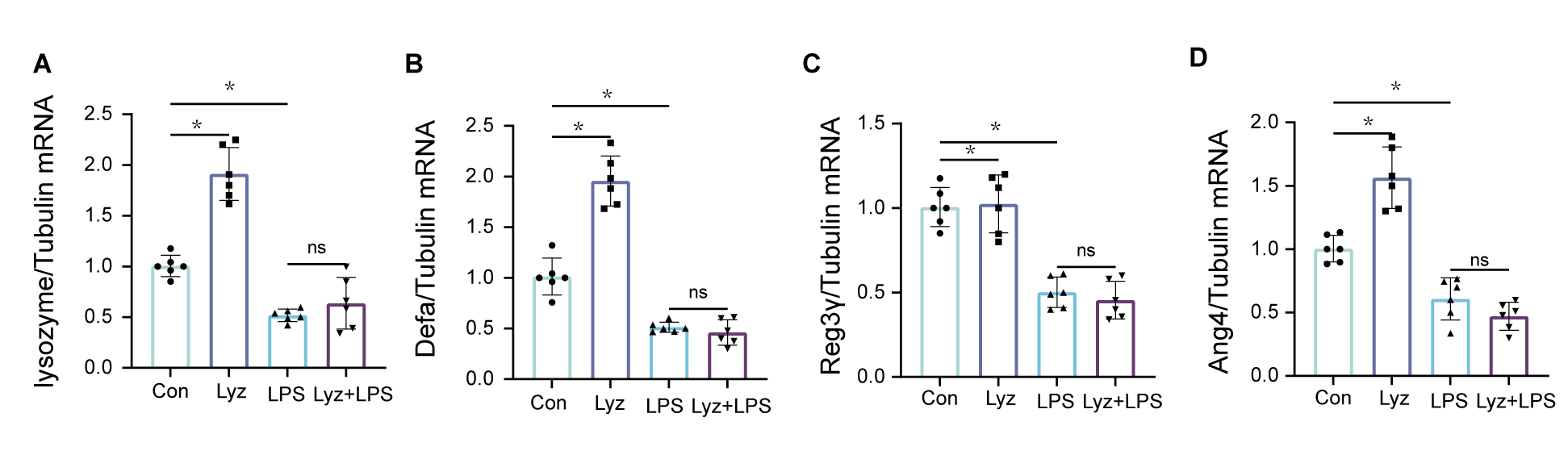

Supplement: FIG S5 [file msystems.01507-21-s0005.tif]

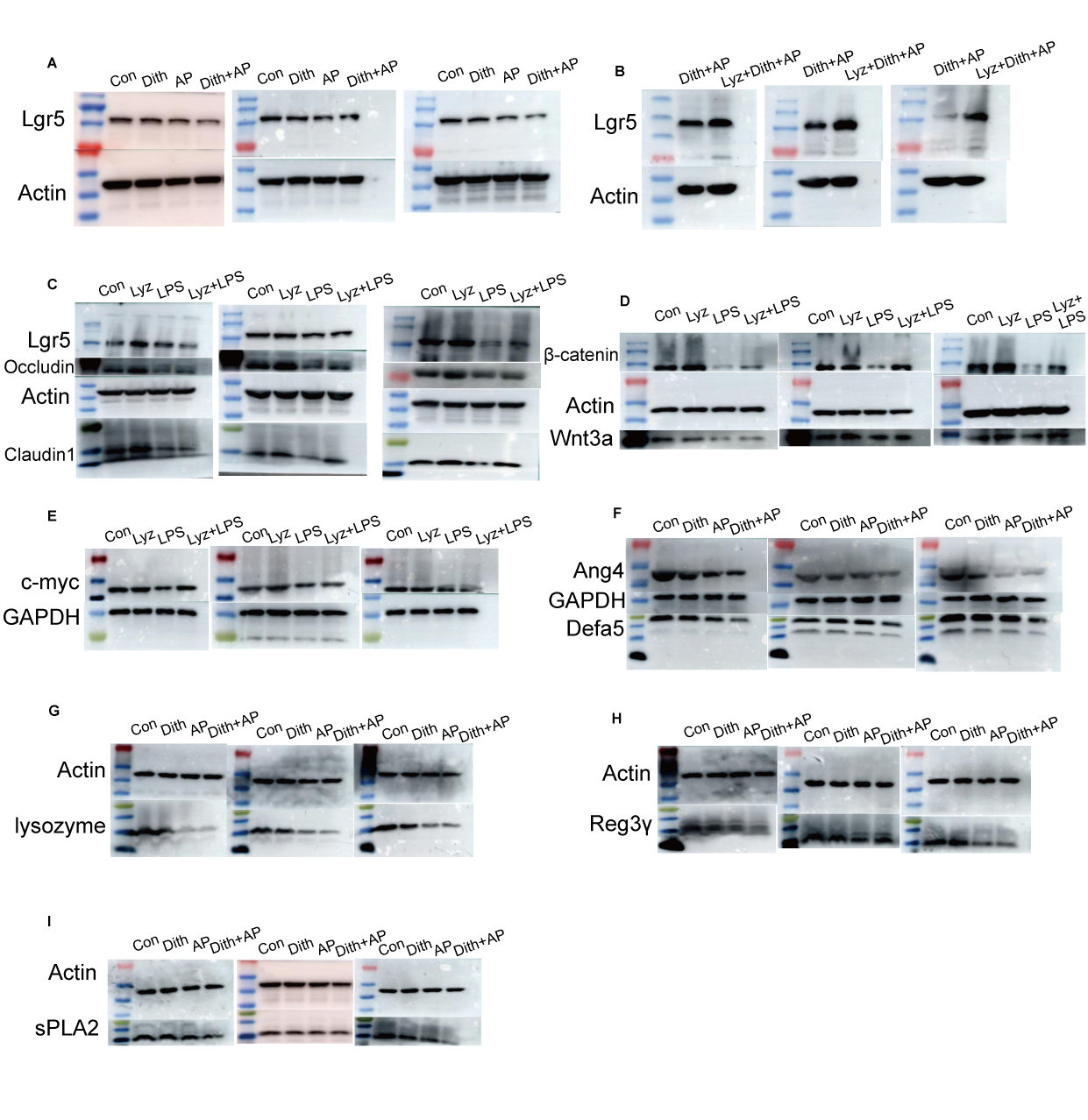

Supplement: FIG S7 [file msystems.01507-21-s0007.tif]
