# Supplementary material for: Paneth Cells Protect against Acute Pancreatitis via Modulating Gut Microbiota Dysbiosis
Source: mSystems. 2022 May 2;7(3):e01507-21. doi: 10.1128/msystems.01507-21 (PMC9239092; doi:10.1128/msystems.01507-21)
Supplement: TABLE S2 [file msystems.01507-21-s0009.docx]

| Name | Sequences (5’-3’) |
| --- | --- |
| Human lysozyme | (F) CTTGTCCTCCTTTCTGTTACGG |
|  | (R) CCCCTGTAGCCATCCATTCC |
| Human HD5 | (F) AGACAACCAGGACCTTGCTAT |
|  | (R) GGAGAGGGACTCACGGGTAG |
| Human HD6 | (F) CTGAGCCACTCCAAGCTGAG |
|  | (R) GTTGAGCCCAAAGCTCTAAGAC |
| Human Reg3γ | (F) GGTGAGGAGCATTAGTAACAGC |
|  | (R) CCAGGGTTTAAGATGGTGGAGG |
| Human Ang4 | (F) CTGGGCGTTTTGTTGTTGGTC |
|  | (R) GGTTTGGCATCATAGTGCTGG |
| Human sPLA2 | (F) ATGAAGACCCTCCTACTGTTGG |
|  | (R) GCTTCCTTTCCTGTCGTCAACT |
| Human Wnt3a | (F) AGCTACCCGATCTGGTGGTC |
|  | (R) CAAACTCGATGTCCTCGCTAC |
| Human Lgr5 | (F) CTCCCAGGTCTGGTGTGTTG |
|  | (R) GAGGTCTAGGTAGGAGGTGAAG |
| Human TGFβ | (F) GGCCAGATCCTGTCCAAGC |
|  | (R) GTGGGTTTCCACCATTAGCAC |
| Human Tubulin | (F) TCTACCTCCCTCACTCAGCT |
|  | (R) CCAGAGTCAGGGGTGTTCAT |
| Mouse lysozyme | (F) ATGGAATGGCTGGCTACTATGG |
|  | (R) ACCAGTATCGGCTATTGATCTGA |
| Mouse Defa5 | (F) CAGGCTGATCCTATCCACAAA |
|  | (R) CTTGGCCTCCAAAGGAGATAG |
| Mouse sPLA2 | (F) CATGGCCTTTGGCTCAATTCAGGT |
|  | (R) ACAGTCATGAGTCACACAGCACCA |
| Mouse Reg3γ | (F) ATGCTTCCCCGTATAACCATCA |
|  | (R) ACTTCACCTTGCACCTGAGAA |
| Mouse Wnt3a | (F) CTCGCTGGCTACCCAATTTG |
|  | (R) CTTCACACCTTCTGCTACGCT |
| Mouse Lgr5 | (F) GGACCAGATGCGATACCGC |
|  | (R) CAGAGGCGATGTAGGAGACTG |
| Mouse TGFβ | (F) CTAATGGTGGACCGCAACAAC |
|  | (R) GCTTCCCGAATGTCTGACGTA |
| Mouse TNFα | (F) CCTGTAGCCCACGTCGTAG |
|  | (R) GGGAGTAGACAAGGTACAACCC |
| Mouse IL-6 | (F) TACCACTTCACAAGTCGGAGGC |
|  | (R) CTGCAAGTGCATCATCGTTGTTC |
| Mouse IL-1β | (F) GAAATGCCACCTTTTGACAGTG |
|  | (R) TGGATGCTCTCATCAGGACAG |
| Mouse β-catenin | (F) ATGGAGCCGGACAGAAAAGC |
|  | (R) TGGGAGGTGTCAACATCTTCTT |
| Mouse c-myc | (F) CCCTATTTCATCTGCGACGAG |
|  | (R) GAGAAGGACGTAGCGACCG |
| Mouse Reg3β | (F) AATGGAGGTGGATGGGAATG |
|  | (R) CCACAGAAAGCACGGTCTAA |
| Mouse MMP7 | (F) CTTACCTCGGATCGTAGTGGA |
|  | (R) CCCCAACTAACCCTCTTGAAGT |
| Mouse cryptdin1 | (F) CTAGTCCTACTCTTTGCCCT |
|  | (R) TTGCAGCCTCTTGATCTACA |
| Mouse cryptdin4 | (F) GTCCAGGCTGATCCTATCCA |
|  | (R) GGGGCAGCAGTACAAAAATC |
| Mouse Tubulin | (F) GGCAGTGTTCGTAGACCTGGAA |
|  | (R) CTCCTTGCCAATGGTGTAGTGG |
| 18s | (F) CTGAGAAACGGCTACCACATC |
|  | (R) GCCTCGAAAGAGTCCTGTATTG |
| *Blautia obeum* | (F) GCGATCAGATTACGATCACTC |
|  | (R) GCCATGCCAACACCTTTTTC |
| *Blautia coccoides* | (F) CGGTACCTGACTAAGAAGC |
|  | (R) AGTTTCATTCTTGCGAACG |
| *Blautia wexlerae* | (F) GCATAAGCGCACAGAGCT |
|  | (R) CACATCAGACTTGCCACA |
| *Helicobacter felis* | (F) GTTTCTGGGGCCAATGTAAG |
|  | (R) GCCTTTGGCAAAACCAATAG |
| *Helicobacter hepaticus* | (F) CCGCAAATTGCAGCAATACTT |
|  | (R) CACCTGTGCATTTTGGACGA |
| *Helicobacter bilis* | (F) CCCGCAATTACCGCATTACTC |
|  | (R) AGTGTGAGATCGCACCTATCATCA |
