# Supplementary material for: Paneth Cells Protect against Acute Pancreatitis via Modulating Gut Microbiota Dysbiosis
Source: mSystems. 2022 May 2;7(3):e01507-21. doi: 10.1128/msystems.01507-21 (PMC9239092; doi:10.1128/msystems.01507-21)
Supplement: Reviewer comments [file reviewer-comments.pdf]

AMERICAN SOCIETY FOR MICROBIOLOGY | mSystems®

Paneth cells protect against acute pancreatitis via modulating gut microbiota dysbiosis

Yang Fu, Qixiang Mei, Nuoming Yin, Zehua Huang, Baiwen Li, Shengzheng Luo, Binqiang Xu, Junjie Fan, Chunlan Huang, and Yue Zeng

Corresponding Author(s): Yue Zeng, Shanghai General Hospital, Shanghai JiaoTong University School of Medicine

Review Timeline:

Submission Date:	December 22, 2021
Editorial Decision:	February 2, 2022
Revision Received:	March 17, 2022
Editorial Decision:	April 5, 2022
Revision Received:	April 6, 2022
Accepted:	April 8, 2022

Editor: Chaysavanh Manichanh

Reviewer(s): Disclosure of reviewer identity is with reference to reviewer comments included in decision letter(s). The following individuals involved in review of your submission have agreed to reveal their identity: Kazuyuki Kasahara (Reviewer #2); PRADEEP BIST (Reviewer #3)

Transaction Report:

(Note: With the exception of the correction of typographical or spelling errors that could be a source of ambiguity, letters and reports are not edited. The original formatting of letters and referee reports may not be reflected in this compilation.)

DOI: https://doi.org/10.1128/msystems.01507-21

February 2, 2022

Dr. Yue Zeng
Shanghai General Hospital, Shanghai JiaoTong University School of Medicine
Shanghai
China

Re: mSystems01507-21 (Paneth cell protect against acute pancreatitis via modulating gut microbiota dysbiosis)

Dear Dr. Yue Zeng:

Thank you for submitting your manuscript to mSystems. We have completed our review and I am pleased to inform you that, in principle, we expect to accept it for publication in mSystems. However, acceptance will not be final until you have adequately addressed the reviewer comments.

Thank you for the privilege of reviewing your work. Below you will find instructions from the mSystems editorial office and comments generated during the review.

Preparing Revision Guidelines
To submit your modified manuscript, log onto the eJP submission site at https://msystems.msubmit.net/cgi-bin/main.plex. Go to Author Tasks and click the appropriate manuscript title to begin the revision process. The information that you entered when you first submitted the paper will be displayed. Please update the information as necessary. Here are a few examples of required updates that authors must address:

• Point-by-point responses to the issues raised by the reviewers in a file named "Response to Reviewers," NOT IN YOUR COVER LETTER.
• Upload a compare copy of the manuscript (without figures) as a "Marked-Up Manuscript" file.
• Each figure must be uploaded as a separate file, and any multipanel figures must be assembled into one file.
• Manuscript: A .DOC version of the revised manuscript
• Figures: Editable, high-resolution, individual figure files are required at revision, TIFF or EPS files are preferred

For complete guidelines on revision requirements, please see the journal Submission and Review Process requirements at https://journals.asm.org/journal/mSystems/submission-review-process. **Submission of a paper that does not conform to mSystems guidelines will delay acceptance of your manuscript.**

Corresponding authors may join or renew ASM membership to obtain discounts on publication fees. Need to upgrade your membership level? Please contact Customer Service at Service@asmusa.org.

Thank you for submitting your paper to mSystems.

The ASM Journals program strives for constant improvement in our submission and publication process. Please tell us how we can improve your experience by taking this quick Author Survey.

Sincerely,

Chaysavanh Manichanh

Editor, mSystems

Journals Department
American Society for Microbiology
1752 N St., NW
Washington, DC 20036
E-mail: peerreview@asmusa.org
Phone: 1-202-942-9338

Reviewer comments:

Reviewer #1 (Comments for the Author):

In their paper Fu et al., use an acute pancreatitis mouse model to elucidate functional pathological changes in the ileum. 16s sequencing showed increases in pathogenic Heliobacter sp. Bacteria accompanied by decreased commensal Blautia sp. The administration of a lysozyme alleviated this shift in the gut microbial population and resulted in decreased clinical characteristics of intestinal pathology.
Overall, the study is well executed, and the findings are supported by the data. Some details remain to be addressed particularly regarding overall quantification of IF images before this work can be considered for publication:
1. It is difficult to see some of the panels perhaps have magnified insets for all focusing on a few cells to demonstrate representative staining. An example is the probe for bacterial endotoxin this is hardly visible.
2. What species of heliobacter are most prevalent? Were the authors able to identify H. pylori? Perhaps I missed this information, but it would add value to discuss the diversity of heliobacter as it could suggest a preferential shift towards one species during pancreatitis.
3. It would be helpful to know the gene expression changes in ileum Paneth cells in AP, AP+Dith, AP+Dith+lyso and Ctrl, using either bulk RNAseq of targeted sorted Paneth cells or sicRNAseq or intestinal epithelium.
4. Please provide cellular quantification in addition to showing representative IF images. For example, in F3 how many cells are Claudin+/DAPI+ or Occludin+/DAPI+ across conditions. This should be performed across all IF images in all figures, for all conditions. It will help strengthen the conclusions.
5. Please provide quantification of all blots, and all blots should be atleast n = 3. Also show whole blot images as a supplementary upload, even if the membrane was cut during imaging.
6. The biggest caveat of this study is the lack of mechanistic insight into how lysozyme is countering the effects of AP-induced ileal microbiome population restructuring. There is a complex interplay between the host ileal epithelium and microbiome that is not explained. However the reviewer understands that this is a work in progress, but outlining this caveat is important in the discussion section.
7. Were there any differences across sex?

Reviewer #2 (Comments for the Author):

The authors recently showed that ablation of Paneth cells exacerbates pancreatic and intestinal injuries and modulates intestinal microbiota in rats with acute pancreatitis (Guo Y et al, Mediators Inflammation 2019). In the current study, they investigated the role of gut microbiota - Paneth cells interactions in a mouse acute pancreatitis (AP) model. Firstly they found that patients with AP had decreased Paneth cells and lower expression of AMP (antimicrobial peptides) genes including lysosome, which was consistent with three mouse models of AP. They established a long-term (i.e., 15 days) reduction of Paneth cells in the L-arginine AP model, which showed increased pancreatic and ileal injuries, intestinal permeability, gut dysbiosis, and bacterial translocation. Moreover, they found that supplementation with lysozyme ameliorated those phenotypes induced by acute pancreatitis in mice and confirmed it in an enteroid model, suggesting that therapeutic interventions targeting Paneth cells provide new strategies for treatment of intestinal complications in AP. There have been enough experiments conducted to provide the conclusion, but there are some critical information missing in the manuscript. The following points should be clarified to prove.

Major concerns/questions:
(1) There are several important information missing in the manuscript. i) experimental methods for the other two AP mouse models (i.e., caerulin+LPS and Na-taurocholate), ii) experimental protocol for antibiotics-treated mice, and iii) clinical trial number. BioProject reference number was provided but it looks like sequencing files are not uploaded in PRJNA774193.
(2) QIIME was used to analyze 16s rRNA sequencing data, but it has not been updated anymore and replaced with QIIME2. Please reanalyze the data with QIIME2. And provide statistical methods used in the microbiome analysis.

(3) Dithizone was used to deplete Paneth cells. What is the specificity of the drug? The reviewer thinks the reagent could directly affect the gut microbiome without changes of Paneth cells.

(4) 200U/day of Lysozyme was supplemented to restore intestinal homeostasis. How did the authors determine the dosage? Any preliminary experiments performed?

Reviewer #3 (Comments for the Author):

"Paneth cell protect against acute pancreatitis via modulating gut microbiota dysbiosis" by Fu et al, claims to describe a mechanism through which the Paneth cells regulate gut microbiota during acute pancreatitis (AP). Using in vivo model, they made an original observation that the reduction in Paneth cells leads to a high risk of AP, and 16S rRNA sequencing revealed an altered gut microbiota landscape with increased abundance of pathogenic bacteria such as Helicobacter with decreased number of beneficial bacteria, Blautia. An imbalance of gut microbiota was found due to poor support for increased intestinal permeability and bacterial translocation, and this phenotype was reversed by supplementing one of the potent antimicrobial peptides

secreted by Paneth cells, lysozyme that played a pivotal role in establishing gut microbiome homeostasis. Mechanistically, they identified signaling molecules such as Wnt, Lgr5, and TGFbeta, important for this reversed phenotype. Furthermore, this study utilizes the organoid in vitro technique to strengthen their research findings. Overall, their transient model system indeed provides an insight into how the Paneth cell's function could be modulated against PA.

What was the source of lysozyme in this study? Would it be good to use the Lysozyme knock-out model to validate the observed phenotype in this study?

Is peritoneal administration of dithizone a more potent route of Paneth cells depletion than an intravenous method of choice?

Corrections-

Title: "Paneth cell protect against acute pancreatitis via modulating gut microbiota dysbiosis" change into "Paneth cells"

Page 2, line 41: remove % from 20 (20-30%)

Page 3, line 61: change "health" into healthy

Page 4, line 79: rephrase "in our study" to this study

Change 16s to 16S in full texts

Figures labeling has to be clearer

Figure S3B: Def to Def5 wherever applicable in the text, and MMP7, Cyrptidin1 panels are missing from the legend.

Page 10, line 189, correct (Figure 10 D-F) to (Figure 10 D)

line 192: mRNA expression of AMPs (lysozyme, Defa5, Spla2, Ang4) were also increased greatly ($p < 0.05$) in Lyz group (Figure 10I, S4A-D): SPLA2 data is missing from the figure.

Figure 10F: Densitometric analysis may help to find the difference between Con to Lyz.

Figure 10J: What is the effect of Lyz on TNF-a, IL-6, or IL-1b expression?

Page 14, line 279: NS, "Mice" to mice

Reviewer #1 (Comments for the Author):

In their paper Fu et al., use an acute pancreatitis mouse model to elucidate functional pathological changes in the ileum. 16s sequencing showed increases in pathogenic Heliobacter sp. Bacteria accompanied by decreased commensal Blautia sp. The administration of a lysozyme alleviated this shift in the gut microbial population and resulted in decreased clinical characteristics of intestinal pathology. Overall, the study is well executed, and the findings are supported by the data. Some details remain to be addressed particularly regarding overall quantification of IF images before this work can be considered for publication:

1. It is difficult to see some of the panels perhaps have magnified insets for all focusing on a few cells to demonstrate representative staining. An example is the probe for bacterial endotoxin this is hardly visible.

Thanks for pointing out the deficiency. We applied universal bacterial probe EUB338 to examine bacterial translocation in pancreas and small intestine. Similar fluorescent intensity was found in our study to those in several other studies (1-3). In our prior manuscript, bacterial translocation was illustrated by representative fluorescent photographs of pancreas (100× magnification) and ileum (200× magnification) and positive cells were counted for quantification. In revised version, we have appended local magnification of the typical positive staining to the upper right corner of the original pictures in Figure 4B, 4D, 8G, 8H to provide more detailed information.

2. What species of heliobacter are most prevalent? Were the authors able to identify H. pylori? Perhaps I missed this information, but it would add value to discuss the diversity of heliobacter as it could suggest a preferential shift towards one species during pancreatitis.

Thanks for the reviewer’s constructive questions. In our study, 16S rRNA sequencing showed that long term reduction of Paneth cells greatly increased the relative abundance of *Helicobacter* and significantly reduced that of *Blautia* in AP. These changes were significantly reversed by lysozyme treatment. Studies have shown that *Helicobacter pylori*, *Helicobacter hepaticus*, *Helicobacter bilis* and *Helicobacter felis* are the most prevalent species associated with gastrointestinal inflammation in *Helicobacter.spp* (4). We carried out real time PCR for these four species to confirm the specific

changes of species in *Helicobacter.spp*.

Compared with Con group, level of *H. felis* and *H. hepaticus* increased significantly in Dith group, while level of *H. bilis* and *H. hepaticus* increased significantly in AP group. Compared with AP group, level of *H. bilis* and *H. felis* decreased and *H. hepaticus* increased significantly in Dith+AP group. Compared with Dith+AP group, level of *H. felis* increased greatly and level of *H. hepaticus* significantly decreased in Lyz+Dith+AP group. Although *H. pylori* has been reported to be associated with prolonged hospital stay in AP patients, it cannot be detected by real time PCR in contents of cecum (data not shown) in our research. These results has been added in **line 188-199** in the revised version **(Figure S3C).**

Based on the analysis of above results, the changes of *H. hepaticus* are of concern and warrants further study. *H. hepaticus* could exacerbated the severity of colitis via inducing inflammatory response, suggesting that it might be involved in intestinal inflammation of AP (5) **(line 272-274).**

We also added results of real time PCR of *Blautia obeum*, *Blautia coccoides* and *Bautia wexlerae* (most prevalent species in *Blautia*) in **line 188-199 (Figure S3C)** and discussion of possible beneficial role of *Blautia obeum* in AP in **line 274-276.**

3. It would be helpful to know the gene expression changes in ileum Paneth cells in AP, AP+Dith, AP+Dith+lyso and Ctrl, using either bulk RNAseq of targeted sorted Paneth cells or sicRNAseq or intestinal epithelium.

Thanks for the reviewer's valuable advice. The method of isolating Paneth cells was described in an article published in *Gastroenterology* in 2021. Briefly, isolated crypts were incubated with TrypLE Express supplement with DNAse I (200 U/ml) and the centrifuged pellet was resuspended and incubated with CD24-PE Ab for 15 min for flow sorting (6). We prepared single cell suspension following the protocol and carried out flow cytometry. Unfortunately, we failed to isolate Paneth cells (data not shown) and bulk RNAseq for Paneth cells was not performed in our study. Due to the limitations on research funds, sicRNAseq of intestinal epithelium is beyond our current capability. Now, we are improving the method of isolation to better explore Paneth cells in AP and other gastrointestinal diseases.

4. Please provide cellular quantification in addition to showing representative IF images. For example, in F3 how many cells are Claudin+/DAPI+ or Occludin+/DAPI+ across conditions. This should be performed across all IF images in all figures, for all conditions. It will help strengthen the conclusions.

Thanks for the reviewer's advice. We have supplemented the quantitative analysis of all fluorescence images including **lysozyme staining** in Figure 1B, 1L, 10I, S2B; **Tunel staining** in 2L 7C, 7G; **EUB338 staining** in Figure 4C, 4E, 8G, 8H; **Claudin1 staining** in 3A, S4G; **ZO-1 staining** in 3B, S4G; **Occludin staining** in 3C, S4G; **PCNA staining** in 3J, 8C, 10D

5. Please provide quantification of all blots, and all blots should be at least n = 3. Also show whole blot images as a supplementary upload, even if the membrane was cut during imaging.

Thanks for the reviewer's valuable suggestion. Your rigorous academic attitudes are worth learning. We have supplemented the quantitative analysis of all blot (Figure S6A-B). All of original images have also been uploaded as part of the supplementary material (Figure S7).

6. The biggest caveat of this study is the lack of mechanistic insight into how lysozyme is countering the effects of AP-induced ileal microbiome population restructuring. There is a complex interplay between the host ileal epithelium and microbiome that is not explained. However the reviewer understands that this is a work in progress, but outlining this caveat is important in the discussion section.

Thanks for the reviewer's constructive suggestion. As you mentioned, there is a complicated interplay between the intestinal epithelium and microbiota. In our study, we focused on Paneth cell and its lysozyme. Lysozyme is a cornerstone of innate immunity, killing bacteria through the hydrolysis of peptidoglycan (PG) and its high cation (7, 8). Previous in vitro studies showed that lysozyme is a non-specific antimicrobial peptide, while different bacteria possessed distinct sensitivity to lysozyme (9-11). For instance, *Lactobacilli* was resistant to lysozyme, while *Dorea* was sensitive (12). In our study, changes of lysozyme are opposite to those of *Helicobacter.spp* and consistent with those of *Blautia.spp*. The relationship between lysozyme and two key genera needed to be verified by further in vitro experiments.

In addition to antimicrobial effect, lysozyme modulated innate immune responses. The sensing

of lysozyme-mediated production of pathogen-associated molecular patterns including PG and lipopolysaccharide by pattern recognition receptor stimulated downstream proinflammatory signaling and the production of proinflammatory cytokines (13, 14).

Lysozyme could also limit intestinal inflammation. Zhang et al showed that intestinal inflammation is associated with the failure of secretion of Paneth cell lysozyme in mouse model of Crohn's disease (15, 16). Furthermore, lysozyme supplement could ameliorate intestinal inflammation of porcine colitis (17). Mechanisms of lysozyme limiting intestinal inflammation was still unclear with speculation of limited bacterial invasion and activated protective intestinal immune response.

In revised manuscript, we outlined this caveat in **line 268-269, 279-286** in Discussion section.

7. Were there any differences across sex?

Thanks for the reviewer's question. In our study, we divided AP patients into two groups based on the course of disease to explore whether the changes of Paneth cells were associated with the course of AP. The Chi-square test on demographic data showed that there was no statistical difference in sex distribution among healthy controls, AP patients in early stage (< 72 h) and AP patients with onset time < 1 week ($p = 0.31$) (in Supplementary Table1). Our research ultimately concluded that AP patients presented dysfunction of Paneth cells regardless of the course of the disease (the detailed results were shown in line 60-72).

We have also regrouped AP patients and healthy controls according to gender to analyze data as you suggested. The figures below illustrate that no significant difference was found in mRNA expression of antimicrobial peptides and stem cell supporting factors across sex in AP patients (data not included in manuscript).

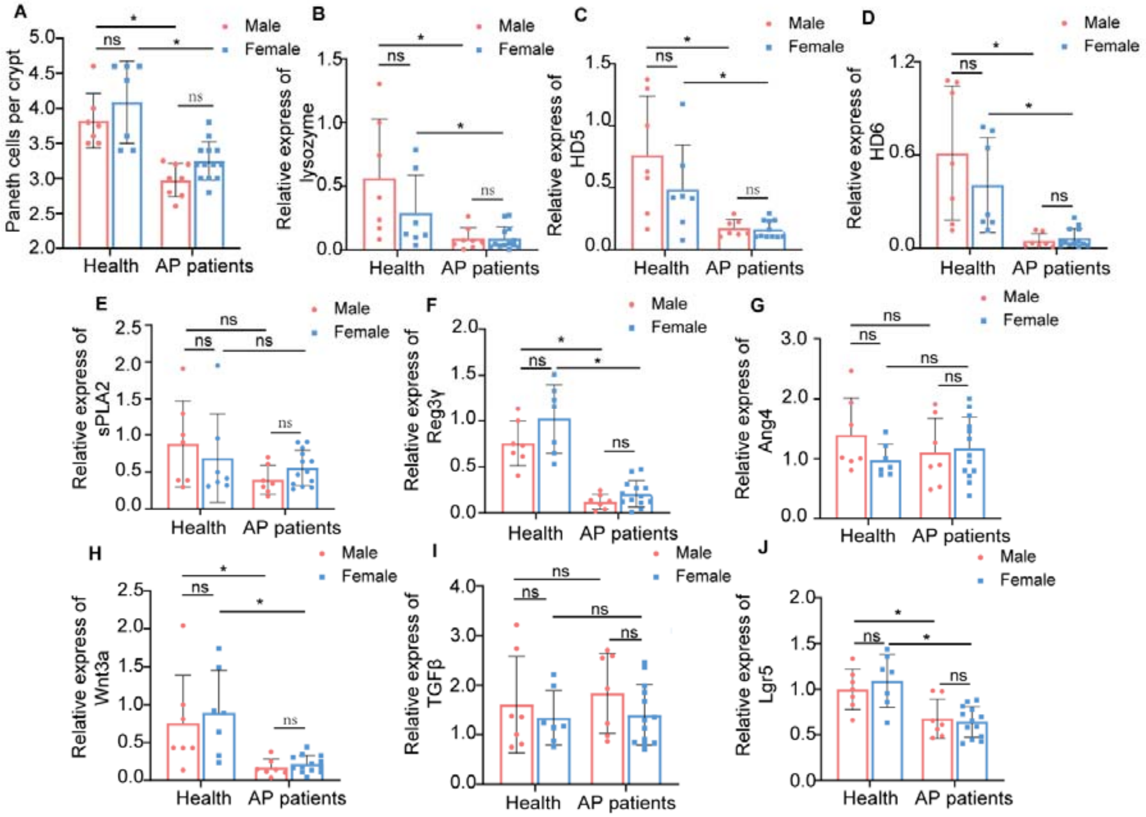

Reviewer #2 (Comments for the Author):

The authors recently showed that ablation of Paneth cells exacerbates pancreatic and intestinal injuries and modulates intestinal microbiota in rats with acute pancreatitis (Guo Y et al, Mediators Inflammation 2019). In the current study, they investigated the role of gut microbiota - Paneth cells interactions in a mouse acute pancreatitis (AP) model. Firstly they found that patients with AP had decreased Paneth cells and lower expression of AMP (antimicrobial peptides) genes including

lysosome, which was consistent with three mouse models of AP. They established a long-term (i.e., 15 days) reduction of Paneth cells in the L-arginine AP model, which showed increased pancreatic and ileal injuries, intestinal permeability, gut dysbiosis, and bacterial translocation. Moreover, they found that supplementation with lysozyme ameliorated those phenotypes induced by acute pancreatitis in mice and confirmed it in an enteroid model, suggesting that therapeutic interventions targeting Paneth cells provide new strategies for treatment of intestinal complications in AP. There have been enough experiments conducted to provide the conclusion, but there are some critical information missing in the manuscript. The following points should be clarified to prove.

Major concerns/questions:

(1) There are several important information missing in the manuscript. i) experimental methods for the other two AP mouse models (i.e., caerulin+LPS and Na-taurocholate), ii) experimental protocol for antibiotics-treated mice, and iii) clinical trial number. BioProject reference number was provided but it looks like sequencing files are not uploaded in PRJNA774193.

Thanks for the reviewer's kind reminding. We were very sorry for missing several critical information. We have complemented the missing contents according to your suggestions.

i) Mice in Cae+AP group were injected intraperitoneally with 100 μg/kg caerulein (MedChemExpress, CN) ten times with an hour interval between consecutive injections. After final injection, 5 mg/kg lipopolysaccharide (Sigma-Aldrich, USA) was intraperitoneally injected. In N+AP group, 2 % sodium taurocholate (Sigma-Aldrich, USA) solution at a volume of 50 μl/20 g bodyweight was infused into the biliopancreatic duct at the speed of 5 μl/min to induce AP. These experimental methods for the two AP mouse models have been added in **line 314-319** in revised manuscript,;

ii) Feces was collected from Con mice, Dith mice and Dith+Lyz mice. The processing of FMT suspension was done within 2 hours. 100 mg feces was resuspended in 1 mL saline and centrifuged for 5 min. The supernatant was used as FMT suspension. The mice receiving antibiotics were treated with vancomycin (0.5 mg/mL), neomycin (1 mg/mL), ampicillin (1 mg/mL), and metronidazole (1 mg/mL) (Sangon Biotech, CN) in their drinking water for 4 weeks. Mice were divided into four groups: Con group, ABX+Con group, ABX+Dith group and ABX+Dith+Lyz group. Con group received no treatment. ABX+Con group were gavaged with feces from Con mice for 1 weeks,

ABX+Dith group were gavaged with 200μL FMT suspension from Dith mice for 1 weeks, and ABX+Dith+Lyz group were gavaged with 200μL FMT suspension from Dith+Lyz mice for 1 weeks. AP was induced in mice from ABX+Con group, ABX+Dith group and ABX+Dith+Lyz group. In revised manuscript, the experimental protocol for antibiotics-treated mice has been added in **line 389-398** in revised manuscript;

iii) Clinical trial number (ChiCTR1800017214) had been marked in Study Approval section (**line 440-441**).

iiii) Thanks for careful review. The data of 16S rRNA sequencing couldn't be downloaded because of some operational errors. National Center for Biotechnology Information (NCBI) staff had assisted us in releasing the data. We have confirmed that the data could be downloaded. If necessary, the reviewer could check the data in BioProject PRJNA774193.

(2) QIIME was used to analyze 16s rRNA sequencing data, but it has not been updated anymore and replaced with QIIME2. Please reanalyze the data with QIIME2. And provide statistical methods used in the microbiome analysis.

Thanks for the reviewer's constructive suggestion. The original data bas been reanalyzed by QIIME2. **Principal coordinate analysis (PCoA)** of Con group, Dith group, AP group and Dith+AP group is in **line 119-122** (**Figure 5E)**, **Alpha diversity** in **line 122-126** **(Figure 5F-H)** and **linear discriminant analysis** (LDA) in **line 133-136** (**Figure 6A-B)**.

PCoA of Dith+AP group and Lyz+Dith+AP group is in **line 179-180** (**Figure 9F)**, **Alpha diversity** in **line 180-182** (**Figure 9G-I)** and **LDA** in **line 185-187 (Figure 9K).**

Statistical methods used in the microbiome analysis have also been updated in the part of Materials and Methods **(line 406-418**).

(3) Dithizone was used to deplete Paneth cells. What is the specificity of the drug? The reviewer thinks the reagent could directly affect the gut microbiome without changes of Paneth cells.

Thanks for the reviewer's question. Dithizone could combine with metal zinc ions to form chelates which had been proved to selectively induce the death of zinc-containing cells in vivo (18). Mitsutaka et al confirmed that intravenous dithizone specifically depleted Paneth cells in the

duodenum and ileum without affecting adjacent crypt base columnar cells (19). Since then, the method of using dithizone to deplete Paneth cells had been widely applied in the study of various diseases, such as necrotizing enterocolitis (NEC), alcoholic steatohepatitis, acute pancreatitis, liver ischemia-reperfusion injury and so on (20-23). By far, no study showed dithizone had a direct effect on intestinal microbiota. Leuschow et al used intraperitoneal injection of dithizone/diphtheria toxin to deplete Paneth cells and induced mouse NEC model respectively. The changes of gut microbiota in dithizone group exhibited a similar trend to that in diphtheria toxin group indicating that dithizone/diphtheria toxin affected the gut microbiota by depleting Paneth cells (24). In our further research, the uncertainty could be interpreted by using mice lacking Paneth cells.

(4) 200U/day of Lysozyme was supplemented to restore intestinal homeostasis. How did the authors determine the dosage? Any preliminary experiments performed?

Thanks for the reviewer's question. The dose of lysozyme is based on the literature published in *Gastroenterology* which confirmed that oral administration of 240 U/day could prevent *Escherichia coli* expansion and visceral hypersensitivity during maternal separation (25). Accordingly, 200 U/day lysozyme were gavaged to mice in Lyz+Dith+AP group for two weeks in our study. Significantly reversed severity of AP aggravated by long-term reduction of Paneth cells and restored dysbiosis of intestinal microbiota were found after lysozyme gavage. Therefore, preliminary experiments had not been arranged in our study with limitation of budget.

Reviewer #3 (Comments for the Author):

"Paneth cell protect against acute pancreatitis via modulating gut microbiota dysbiosis" by Fu et al, claims to describe a mechanism through which the Paneth cells regulate gut microbiota during acute pancreatitis (AP). Using in vivo model, they made an original observation that the reduction in Paneth cells leads to a high risk of AP, and 16S rRNA sequencing revealed an altered gut microbiota landscape with increased abundance of pathogenic bacteria such as Helicobacter with decreased number of beneficial bacteria, Blautia. An imbalance of gut microbiota was found due to poor support for increased intestinal permeability and bacterial translocation, and this phenotype was

reversed by supplementing one of the potent antimicrobial peptides secreted by Paneth cells, lysozyme that played a pivotal role in establishing gut microbiome homeostasis. Mechanistically, they identified signaling molecules such as Wnt, Lgr5, and TGFbeta, important for this reversed phenotype. Furthermore, this study utilizes the organoid in vitro technique to strengthen their research findings. Overall, their transient model system indeed provides an insight into how the Paneth cell's function could be modulated against PA.

What was the source of lysozyme in this study? Would it be good to use the Lysozyme knock-out model to validate the observed phenotype in this study?

Thanks for the reviewer's question. In our research, we used lysozyme from chicken egg white (L6876, Sigma, USA) (line 305) referred to the study published in *Gastroenterology*.

Lyz1$^{-/-}$ mice had been used to demonstrate lysozyme could generate ligands to modulate Nod2 activation and regulated the intestinal inflammatory response (12, 26). The application of knockout mice might further verify the critical role of lysozyme in AP. However, due to the limitations of budget and objective conditions, Lyz1$^{-/-}$ mice were not used in our study.

Supplementation of α-defensin5 effectively altered gut dysbiosis induced by Paneth cell dysfuncton in alcoholic hepatitis (21). Lysozyme supplementation prevented *Escherichia coli* expansion caused by Paneth cell defect during maternal isolation (25). Lysozyme could also ameliorate intestinal inflammation of colitis (17). Therefore, we speculated that supplementation of lysozyme could attenuate gut dysbiosis induced by long term reduction of Paneth cells and alleviate intestinal inflammation of AP. Our study ultimately showed lysozyme significantly reduced the severity of AP.

Is peritoneal administration of dithizone a more potent route of Paneth cells depletion than an intravenous method of choice?

Thanks for the reviewer's question. The mechanism that dithizone selectively depleted Paneth cells was that it could bind with zinc ions to form zinc chelate which leads to cell death (19). Intraperitoneal and intravenous injection of dithizone are both widely recognized and deplete Paneth cells effectively. In the latest ten articles depletion of Paneth cells by dithizone in mice with doses ranged from 33 mg/kg to 100 mg/kg, nine studies chose intraperitoneal injection (20, 21, 24, 27-33). Moreover, our previous study showed rats injected with dithizone (100 mg/kg body weight) via the

tail vein were associated with high mortality rate (34). Therefore, we ultimately chose intraperitoneal injection of dithizone in our study.

Corrections-

Title: "Paneth cell protect against acute pancreatitis via modulating gut microbiota dysbiosis" change into "Paneth cells"

Page 2, line 41: remove % from 20 (20-30%)

Page 3, line 61: change "health" into healthy

Page 4, line 79: rephrase "in our study" to this study

Change 16s to 16S in full texts

Figures labeling has to be clearer

Figure S3B: Def to Def5 wherever applicable in the text, and MMP7, Cyrptidin1 panels are missing from the legend.

Page 10, line 189, correct (Figure 10 D-F) to (Figure 10 D)

line 192: mRNA expression of AMPs (lysozyme, Defa5, Spla2, Ang4) were also increased greatly ($p < 0.05$) in Lyz group (Figure 10I, S4A-D): SPLA2 data is missing from the figure.

Figure 10F: Densitometric analysis may help to find the difference between Con to Lyz.

Figure 10J: What is the effect of Lyz on TNF-a, IL-6, or IL-1b expression?

Page 14, line 279: NS, "Mice" to mice

We thank the distinguished reviewer for careful review of our manuscript. We've corrected all mistakes or insufficiencies you mentioned above. Lysozyme treatment did not change the expression of inflammatory factors (TNF-α, IL-6, or IL-1β) in enteroids. We added the comparison of inflammatory factors between Con group and Lyz group in Figure 10J.

Reference

1. Li X, He C, Li N, Ding L, Chen H, Wan J, Yang X, Xia L, He W, Xiong H, Shu X, Zhu Y, Lu N. 2020. The interplay between the gut microbiota and NLRP3 activation affects the severity of acute pancreatitis in mice. Gut Microbes 11:1774-1789.

2. Wang L, Fouts DE, Stärkel P, Hartmann P, Chen P, Llorente C, DePew J, Moncera K, Ho SB, Brenner DA, Hooper LV, Schnabl B. 2016. Intestinal REG3 Lectins Protect against Alcoholic Steatohepatitis by Reducing Mucosa-Associated Microbiota and Preventing Bacterial Translocation. Cell Host Microbe 19:227-39.

3. Wen W, Zheng H, Jiang Y, Huang L, Li D, Zhang J, Zhang D. 2017. Effect of intestinal epithelial autophagy on bacterial translocation in severe acute pancreatitis. Clin Res Hepatol Gastroenterol 41:703-710.

4. Smet A, Menard A. 2020. Review: Other Helicobacter species. Helicobacter 25 Suppl 1:e12744.

5. Zhu L, Zhu C, Cao S, Zhang Q. 2021. Helicobacter hepaticus Induce Colitis in Male IL-10(-/-) Mice Dependent by Cytolethal Distending Toxin B and via the Activation of Jak/Stat Signaling Pathway. Front Cell Infect Microbiol 11:616218.

6. Lu R, Zhang YG, Xia Y, Zhang J, Kaser A, Blumberg R, Sun J. 2021. Paneth Cell Alertness to Pathogens Maintained by Vitamin D Receptors. Gastroenterology 160:1269-1283.

7. Zhang X, Jiang A, Yu H, Xiong Y, Zhou G, Qin M, Dou J, Wang J. 2016. Human Lysozyme Synergistically Enhances Bactericidal Dynamics and Lowers the Resistant Mutant Prevention

Concentration for Metronidazole to Helicobacter pylori by Increasing Cell Permeability. Molecules 21.

8. Callewaert L, Michiels CW. 2010. Lysozymes in the animal kingdom. J Biosci 35:127-60.

9. Ellison RT, 3rd, Giehl TJ. 1991. Killing of gram-negative bacteria by lactoferrin and lysozyme. J Clin Invest 88:1080-91.

10. Ragland SA, Criss AK. 2017. From bacterial killing to immune modulation: Recent insights into the functions of lysozyme. PLoS Pathog 13:e1006512.

11. Laible NJ, Germaine GR. 1985. Bactericidal activity of human lysozyme, muramidase-inactive lysozyme, and cationic polypeptides against Streptococcus sanguis and Streptococcus faecalis: inhibition by chitin oligosaccharides. Infect Immun 48:720-8.

12. Yu S, Balasubramanian I, Laubitz D, Tong K, Bandyopadhyay S, Lin X, Flores J, Singh R, Liu Y, Macazana C, Zhao Y, Béguet-Crespel F, Patil K, Midura-Kiela MT, Wang D, Yap GS, Ferraris RP, Wei Z, Bonder EM, Häggblom MM, Zhang L, Douard V, Verzi MP, Cadwell K, Kiela PR, Gao N. 2020. Paneth Cell-Derived Lysozyme Defines the Composition of Mucolytic Microbiota and the Inflammatory Tone of the Intestine. Immunity 53:398-416.e8.

13. Wolf AJ, Arruda A, Reyes CN, Kaplan AT, Shimada T, Shimada K, Arditi M, Liu G, Underhill DM. 2011. Phagosomal degradation increases TLR access to bacterial ligands and enhances macrophage sensitivity to bacteria. J Immunol 187:6002-10.

14. Caruso R, Warner N, Inohara N, Núñez G. 2014. NOD1 and NOD2: signaling, host defense, and inflammatory disease. Immunity 41:898-908.

15. Zhang Q, Pan Y, Yan R, Zeng B, Wang H, Zhang X, Li W, Wei H, Liu Z. 2015. Commensal bacteria direct selective cargo sorting to promote symbiosis. Nat Immunol 16:918-26.

16. Wang H, Zhang X, Zuo Z, Zhang Q, Pan Y, Zeng B, Li W, Wei H, Liu Z. 2017. Rip2 Is Required for

Nod2-Mediated Lysozyme Sorting in Paneth Cells. J Immunol 198:3729-3736.

17. Lee M, Kovacs-Nolan J, Yang C, Archbold T, Fan MZ, Mine Y. 2009. Hen egg lysozyme attenuates inflammation and modulates local gene expression in a porcine model of dextran sodium sulfate (DSS)-induced colitis. J Agric Food Chem 57:2233-40.

18. Okamoto K. 1955. Experimental pathology of diabetes mellitus. II. Tohoku J Exp Med 61:1-116.

19. Sawada M, Takahashi K, Sawada S, Midorikawa O. 1991. Selective killing of Paneth cells by intravenous administration of dithizone in rats. Int J Exp Pathol 72:407-21.

20. McElroy SJ, Castle SL, Bernard JK, Almohazey D, Hunter CJ, Bell BA, Al Alam D, Wang L, Ford HR, Frey MR. 2014. The ErbB4 ligand neuregulin-4 protects against experimental necrotizing enterocolitis. Am J Pathol 184:2768-78.

21. Zhong W, Wei X, Hao L, Lin TD, Yue R, Sun X, Guo W, Dong H, Li T, Ahmadi AR, Sun Z, Zhang Q, Zhao J, Zhou Z. 2020. Paneth Cell Dysfunction Mediates Alcohol-related Steatohepatitis Through Promoting Bacterial Translocation in Mice: Role of Zinc Deficiency. Hepatology 71:1575-1591.

22. Guo Y, Huang C, Liu L, Fu X, Lu Y, Zheng J, Mei Q, Huang Z, Fan J, Lu L, Zeng Y. 2019. Paneth Cell Ablation Aggravates Pancreatic and Intestinal Injuries in a Rat Model of Acute Necrotizing Pancreatitis after Normal and High-Fat Diet. Mediators Inflamm 2019:8474523.

23. Grootjans J, Hodin CM, de Haan JJ, Derikx JP, Rouschop KM, Verheyen FK, van Dam RM, Dejong CH, Buurman WA, Lenaerts K. 2011. Level of activation of the unfolded protein response correlates with Paneth cell apoptosis in human small intestine exposed to ischemia/reperfusion. Gastroenterology 140:529-539.e3.

24. Lueschow SR, Stumphy J, Gong H, Kern SL, Elgin TG, Underwood MA, Kalanetra KM, Mills DA, Wong MH, Meyerholz DK, Good M, McElroy SJ. 2018. Loss of murine Paneth cell function alters the

immature intestinal microbiome and mimics changes seen in neonatal necrotizing enterocolitis. PLoS One 13:e0204967.

25. Riba A, Olier M, Lacroix-Lamandé S, Lencina C, Bacquié V, Harkat C, Gillet M, Baron M, Sommer C, Mallet V, Salvador-Cartier C, Laurent F, Théodorou V, Ménard S. 2017. Paneth Cell Defects Induce Microbiota Dysbiosis in Mice and Promote Visceral Hypersensitivity. Gastroenterology 153:1594-1606.e2.

26. Wang H, Shen X, Zheng X, Pan Y, Zhang Q, Liu Z. 2021. Intestinal lysozyme releases Nod2 ligand(s) to promote the intestinal mucosal adjuvant activity of cholera toxin. Sci China Life Sci 64:1720-1731.

27. Zhang C, Sherman MP, Prince LS, Bader D, Weitkamp JH, Slaughter JC, McElroy SJ. 2012. Paneth cell ablation in the presence of Klebsiella pneumoniae induces necrotizing enterocolitis (NEC)-like injury in the small intestine of immature mice. Dis Model Mech 5:522-32.

28. Fernandez MI, Regnault B, Mulet C, Tanguy M, Jay P, Sansonetti PJ, Pédron T. 2008. Maturation of paneth cells induces the refractory state of newborn mice to Shigella infection. J Immunol 180:4924-30.

29. Berger JN, Gong H, Good M, McElroy SJ. 2019. Dithizone-induced Paneth cell disruption significantly decreases intestinal perfusion in the murine small intestine. J Pediatr Surg 54:2402-2407.

30. Lueschow SR, Kern SL, Gong H, Grobe JL, Segar JL, Carlson SJ, McElroy SJ. 2020. Feeding Formula Eliminates the Necessity of Bacterial Dysbiosis and Induces Inflammation and Injury in the Paneth Cell Disruption Murine NEC Model in an Osmolality-Dependent Manner. Nutrients 12.

31. White JR, Gong H, Pope B, Schlievert P, McElroy SJ. 2017. Paneth-cell-disruption-induced necrotizing enterocolitis in mice requires live bacteria and occurs independently of TLR4 signaling. Dis Model Mech 10:727-736.

32. Chaaban H, Burge K, Eckert J, Keshari RS, Silasi R, Lupu C, Warner B, Escobedo M, Caplan M, Lupu F. 2021. Neutrophil extracellular trap inhibition increases inflammation, bacteraemia and mortality in murine necrotizing enterocolitis. J Cell Mol Med 25:10814-10824.

33. Park SW, Kim M, Brown KM, D'Agati VD, Lee HT. 2011. Paneth cell-derived interleukin-17A causes multiorgan dysfunction after hepatic ischemia and reperfusion injury. Hepatology 53:1662-75.

34. Liu L, Guo Y, Zheng J, Lu Y, Shen Y, Huang C, Zeng Y, Wang X. 2019. Paneth cell ablation increases the small intestinal injury during acute necrotizing pancreatitis in rats. Mol Med Rep 20:473-484.

April 5, 2022

Dr. Yue Zeng
Shanghai General Hospital, Shanghai JiaoTong University School of Medicine
Shanghai
China

Re: mSystems01507-21R1 (Paneth cells protect against acute pancreatitis via modulating gut microbiota dysbiosis)

Dear Dr. Yue Zeng:

Thank you for submitting your manuscript to mSystems. We have completed our review and I am pleased to inform you that, in principle, we expect to accept it for publication in mSystems. However, acceptance will not be final until you have adequately addressed reviewer #3 comments.

Thank you for the privilege of reviewing your work. Below you will find instructions from the mSystems editorial office and comments generated during the review.

Preparing Revision Guidelines
To submit your modified manuscript, log onto the eJP submission site at https://msystems.msubmit.net/cgi-bin/main.plex. Go to Author Tasks and click the appropriate manuscript title to begin the revision process. The information that you entered when you first submitted the paper will be displayed. Please update the information as necessary. Here are a few examples of required updates that authors must address:

• Point-by-point responses to the issues raised by the reviewers in a file named "Response to Reviewers," NOT IN YOUR COVER LETTER.
• Upload a compare copy of the manuscript (without figures) as a "Marked-Up Manuscript" file.
• Each figure must be uploaded as a separate file, and any multipanel figures must be assembled into one file.
• Manuscript: A .DOC version of the revised manuscript
• Figures: Editable, high-resolution, individual figure files are required at revision, TIFF or EPS files are preferred

ASM policy requires that data be available to the public upon online posting of the article, so please verify all links to sequence records, if present, and make sure that each number retrieves the full record of the data. If a new accession number is not linked or a link is broken, provide production staff with the correct URL for the record. If the accession numbers for new data are not publicly accessible before the expected online posting of the article, publication of your article may be delayed; please contact the ASM production staff immediately with the expected release date.

For complete guidelines on revision requirements, please see the journal Submission and Review Process requirements at https://journals.asm.org/journal/mSystems/submission-review-process. **Submission of a paper that does not conform to mSystems guidelines will delay acceptance of your manuscript.**

Corresponding authors may join or renew ASM membership to obtain discounts on publication fees. Need to upgrade your membership level? Please contact Customer Service at Service@asmusa.org.

Thank you for submitting your paper to mSystems.

The ASM Journals program strives for constant improvement in our submission and publication process. Please tell us how we can improve your experience by taking this quick Author Survey.

Sincerely,

Chaysavanh Manichanh

Editor, mSystems

Journals Department
American Society for Microbiology
1752 N St., NW
Washington, DC 20036

E-mail: peerreview@asmusa.org
Phone: 1-202-942-9338

Reviewer comments:

Reviewer #1 (Comments for the Author):

The authors have sufficiently:
1. Applied correct analytical models to study statistical differences in cellular populations.
2) Added new novel data in relation to their study to look at Heliobacter sp. opening up new avenues of study.
3) Outlined limitations owing to technical and funding limitations, which is a part of ongoing science.

The authors have therefore sufficiently addressed my concerns. Any typographical errors including syntax, missing references, and grammatical deficiencies can be handled by the handling editor and typesetting editors of this journal.

Reviewer #2 (Comments for the Author):

The reviewer carefully read the responses to the review comment and the revised manuscript. All concerns have been addressed and it is acceptable for publication. Congratulations!

Reviewer #3 (Comments for the Author):

The authors have taken enough care to improve the manuscript by providing the required information. However, there are minor grammatic errors that need to be taken into consideration. The changes are highlighted in the manuscript (pdf version).

Paneth cells protect against acute pancreatitis via modulating gut microbiota dysbiosis

Yang Fu[1,2]*, Qixiang Mei[1,2]*, Nuoming Yin[1,2]*, Zehua Huang[1,2], Baiwen Li[2], Shengzheng Luo[2], Binqiang Xu[1,2], Junjie Fan[2], Chunlan Huang[1,2], Yue Zeng[1,2]

1. Shanghai Key Laboratory of Pancreatic Disease, Shanghai JiaoTong University School of Medicine, Shanghai 201600, China

2. Department of Gastroenterology, Shanghai General Hospital, Shanghai JiaoTong University School of Medicine, Shanghai 201600, China

*These authors contributed equally to this work.

Corresponding author: Yue Zeng, Chunlan Huang, Junjie Fan, Department of Gastroenterology, Shanghai General Hospital, Shanghai JiaoTong University School of Medicine, Shanghai 201600, China; E-mail: carrie_1004@sjtu.edu.cn, huangchl@hotmail.com and 956937215@qq.com

Abstract

Acute pancreatitis (AP) was usually accompanied by intestinal failure, but its mechanism was still unclear. In AP patients, functions of Paneth cells (lysozyme, HD5, Reg3γ and Wnt3a) decreased. Compared with AP mice, injuries and inflammations of pancreas and ileum were aggravated in Dithizone (Dith) +AP mice. Intestinal permeability and bacteria translocation were also increased. 16S rRNA sequencing showed that gut microbiota of Dith mice and Dith+AP mice exhibited a markedly increase in pathogenic bacteria *Helicobacter* and a significant~~ly~~ decrease in probiotics *Blautia*. Lysozyme gavage in Dith+AP mice effectively alleviated injuries of the pancreas and small intestine. The beneficial effect of lysozyme was associated with a significant~~ly~~ increase in probiotics *Blautia* and a virtual~~ly~~ absence of pathogenic bacteria *Helicobacter*. The severity of AP in antibiotics treated (ABX) mice was significantly aggravated when receiving feces from Dith mice and was markedly alleviated when receiving feces from lysozyme gavaged mice. In vitro, lysozyme increased the proliferation of enteroids by promoting activation of the Wnt pathway and Lgr5 expression of intestinal stem cells.

Importance

We demonstrated that AP patients and experimental AP mice exhibited dysfunction of Paneth cells. Our in vivo research showed that the severity of AP was exacerbated by long term dysfunction of Paneth cells which was associated with gut microbiota disorder. Restoring part of Paneth cell function through lysozyme supplementation alleviated the severity of AP and gut microbiota dysbiosis. This study provided a novel insight into a link of pancreas-gut interaction in the pathogenesis of AP, providing a new direction for clinical treatment of intestinal complications during AP.

Keywords

acute pancreatitis; Paneth cell; gut microbiota; lysozyme; intestinal enteroid

Introduction

Acute pancreatitis (AP) is one of the most common gastrointestinal diseases requiring urgent hospitalization (1). Approximately 20-30% of patients develop severe acute pancreatitis (SAP) with a substantial mortality rate of 20-40% (2). The translocation of intestinal bacteria and endotoxin after intestinal barrier injury is a key event leading to SAP (3). A growing number of studies revealed that intestinal microecology alteration is related to the development of AP, which includes microbiota dysbiosis, intestinal barrier damage, and immunological dysfunction (3-6). But mechanisms have not yet been well understood and require further elucidation.

Paneth cells are highly differentiated secretory cells in the intestinal epithelium (7). They are distributed in the intestinal crypts and play an important role in the intestinal barrier. These cells secrete antimicrobial peptides (AMPs) such as lysozyme and α-defensin to maintain the homeostasis of the intestinal environment (8, 9). Paneth cells also serve as guardians of intestinal stem cells via providing essential cytokines such as Wnt3a and TGFβ (10). Its abnormality is related to the progression of a variety of diseases (11-13). Our previous study proved that transient ablation of Paneth cell by dithizone (Dith) aggravated pancreatic and intestinal injuries in rat AP (14). An interaction exists between gut microbiota and Paneth cells. Mice lacking intestinal Sox9 protein presented an absence of Paneth cells accompanied by an increase of *Bacteroidetes* and *Enterococcus* and a decrease of *Bifidobacterium* (15). While gut microbiota regulates Paneth cell number and functions (16).

In this study, we explored the role of gut microbiota regulated by Paneth cells in AP and the potential therapeutic effects of lysozyme on AP by in vivo and in vitro experiments.

Results

Dysfunction of Paneth cells in AP patients and experimental AP mice. We collected duodenal mucosa specimens through endoscopy from 21 AP patients and 14 healthy controls. AP patients were divided into two groups based on the course of the disease to explore whether the changes of Paneth cells were associated with the course of AP. No demographic differences were found among the three groups (Table S1). Compared with healthy controls, Paneth cell counting and protein expression of lysozyme in duodenal were significantly decreased ($p < 0.05$) in AP patients in ~~early~~ early-stage (< 72 h) or with onset time < 1 week (Figure 1A-B). AP patients in ~~early~~ early-stage had lower ($p < 0.05$) mRNA expression of lysozyme, human defensin (HD) 5, HD6, and regenerating islet-derived (Reg) 3γ, Wnt3a, and Lgr5 than those in healthy controls (Figure 1C-H). Significantly reduced mRNA expression ($p < 0.05$) of lysozyme, HD5, HD6, Reg3γ, and Wnt3a were also found in AP patients with onset time < 1 week (Figure 1C-H). However, compared with healthy controls, the expression of angiogenin 4 (Ang4)~~–~~, secretory phospholipase A2 (sPLA2), and TGFβ did not change greatly no matter how long the disease lasted (Figure 1I-K). Therefore, dysfunctions of Paneth cells were detected in AP patients regardless of the course of the disease.

We applied three classical mouse models of AP, which were the L-arginine model (L-AP), caerulin+LPS model (Cae-AP) and Na-taurocholate model (N-AP) to further validate these findings. Compared with control (Con) mice, the number of Paneth cells in crypts and expression of lysozyme decreased significantly $p < 0.05$) in three AP models (Figure 1L-M). Lysozyme, α-defensin5 (Defa5), Reg3γ, Wnt3a, Lgr5, and TGFβ were reduced at the mRNA level (Figure S1A-B).

Long term reduction of Paneth cells aggravated AP-induced injuries, inflammation and

bacterial translocation. In our previous study, rats were treated intraperitoneally with 100 mg/kg dithzone to ablate Paneth cells for 48 h (14). In this study, by increasing the frequency of injection and adjusting the dose of dithizone, the number of Paneth cells in mice intestinal crypts and expression of lysozyme and Defa5 decreased approximately to one one-half of original levels for two weeks (Figure S2A-E). AP was induced by L-arginine on the basis of long long-term reduction of Paneth cells (Figure S2A). Pancreatic pathological injuries and inflammation, amylase level, and pancreatic wet to dry (W/D) weight ratio of Dith+AP mice reached the highest level at 3 day following AP induction and were more severe ($p < 0.05$) than those of AP mice (Figure 2A-D). Therefore, we chose 3 day as the optimal time for subsequent experiments.

Dith+AP mice also exhibited more severe ileal pathological injuries ($p < 0.05$) and more robust increases ($p < 0.05$) of serum and ileal proinflammatory cytokines (TNF-α, IL-6 and IL-1β) compared with AP mice (Figure 2E-K). Tunel staining showed that long term reduction of Paneth cells caused more ileal apoptosis ($p < 0.05$) in Dith+AP mice (Figure 2L). Compared with AP mice, the expression of tight junction proteins (TJPs) (claudin1, occludin and ZO-1) decreased in Dith+AP mice (Figure 3A-D). Serum diamine oxidase (DAO) and D-lactate levels were also higher in Dith+AP mice than those in AP mice ($p < 0.05$), suggesting increased intestinal permeability (Figure 3E).

It has been proved that Paneth cells constituted the niche for $Lgr5^+$ stem cells in intestinal crypts by secreting Wnt3a, TGFβ, etc (10). Compared with AP mice, the expression of Wnt3a, TGFβ and Lgr5 were significantly down-regulated ($p < 0.05$) in Dith+AP mice (Figure 3F-I). Proliferating cell nuclear antigen (PCNA) staining showed that the reduction of Paneth cells inhibited the proliferation of intestinal epithelial cells (IECs) in Dith+AP mice (Figure 3J).

The progression of AP involves an increase in bacterial translocation caused by the disrupted intestinal barriers. Dith+AP mice harbored a higher level of endotoxin ($p < 0.05$) than AP mice (Figure 4A). Compared with AP mice, no bacterial translocation was found in Dith mice, while Dith+AP mice presented increased bacterial translocation to intestinal mucosa and pancreas using fluorescence in situ hybridization (FISH) analysis (Figure 4B-E). The amount of anaerobic bacteria in liver and mesenteric lymph nodes ~~was~~ were counted through a brain heart infusion agar (BHIA) plate. Compared with AP mice, Dith+AP mice had more ~~colony~~ colony-forming units (CFUs) ($p < 0.05$), indicating that liver and mesenteric lymph nodes of Dith+AP mice had more severe bacterial translocation (Figure 4F-G).

The aggravation of AP in Dith mice was related to the disturbance of intestinal microbiota. To explore the role of the gut microbiota in exacerbated injuries and inflammation of Dith+AP mice, we transplanted the fecal microbiota of Dith mice and Con mice to antibiotics treated (ABX) mice followed by induction of AP. Compared with those of ABX mice receiving feces from Con mice, pathological damage and mRNA expression levels of TNF-α, IL-6 and IL-1β were increased significantly ($p < 0.05$) in both pancreatic and ileal tissues of ABX mice receiving feces from Dith mice (Figure 5A-D).

We then analyzed the cecal contents by 16S rRNA sequencing. Principal coordinate analysis (PCoA) showed that the intestinal microbiota of AP mice ~~were~~ was largely separated from that of Con mice. Simultaneously, different bacterial communities were revealed between Con mice and Dith mice. There was a partial overlap between bacterial communities in Dith and Dith+AP mice (Figure 5E). Compared with Con mice, alpha diversity of bacterial communities was greatly decreased in Dith mice reflected by decreased ~~shannon~~ Shannon index ($p < 0.05$) and increased ~~simpson~~ Simpson index ($p < 0.05$) (Figure 5F-H). Compared with AP mice, alpha diversity was also markedly decreased in Dith+AP mice evidenced by

decreased ~~shannon~~ Shannon index, chao1 index, and increased ~~simpson~~ Simpson index (Figure 5F-H).

Firmicutes and *Bacteroidetes* are two dominant bacteria at the phylum level (17). An increase of the relative abundance of *Firmicutes* and a decrease of relative abundance of *Bacteroidetes* resulted in an increase of *Firmicutes/Bacteroidetes* (F/B) ratio (p < 0.05) in Dith mice and Dith+AP mice (Figure 5I-J, S3A). The relative abundance of *Proteobacteria,* which includes *Escherichia-shigella, Helicobacter* and other pathogenic bacteria (18), also tended to increase in Dith mice and Dith+AP mice ($p < 0.05$) (Figure 5I-J).

Then we performed linear discrimination analysis coupled with effect size (LEfSe) on gut microbiota between Con mice and Dith mice with or without AP. At the genus level, the relative abundance of *Bacteroides* and *Helicobacter* increased significantly ($p < 0.05$), while the relative abundance of *Blautia* decreased markedly in Dith mice and Dith+AP mice ($p < 0.05$) (Figure 6A-B). Spearman's correlation analysis showed that the relative abundance of *Helicobacter* are positively correlated with levels of a pancreatic and ileal histopathological score, serum DAO and D-lactate, serum proinflammatory cytokines and pancreatic MPO. In contrast, the relative abundance of *Blautia* ~~were~~ was negatively associated with an ileal histopathological score, serum IL-6, pancreatic MPO, and endotoxin (Figure 6C).

Lysozyme ameliorated AP-induced injuries and inflammation in Dith mice. Functional recovery of Paneth cell has been reported to effectively correct intestinal dysbiosis (19). We next evaluated the therapeutic potential of AMPs of Paneth cells in AP mice. Compared with AP mice, the mRNA expression of lysozyme, Defa5, Reg3γ, Reg3β, Ang4, cryptdin1 in Dith+AP mice decreased significantly ($p < 0.05$), while the mRNA expression of MMP7 and sPLA2 were not significant changes (Figure S4A). The protein expression of lysozyme, Defa5, and Ang4 were down-regulated in Dith+AP mice (Figure S4B, S6A).

Spearman's correlation analysis revealed that the severity of AP was inversely associated with the levels of Paneth cell AMPs, where lysozyme harbored the highest correlation coefficient (Figure S4C). Therefore, we chose supplementation of lysozyme as the functional recovery of Paneth cells to restore the intestinal homeostasis.

We observed less severe pancreatic injuries and apoptosis as well as much lower amylase level and pancreatic W/D weight ratio in Lyz+Dith+AP mice than those in Dith+AP mice ($p < 0.05$) (Figure 7A-D). The pancreatic inflammation was attenuated in Lyz+Dith+AP mice ($p < 0.05$), as evidenced by the decline in proinflammatory cytokines (Figure 7E). Compared with Dith+AP mice, Lyz+Dith+AP mice also showed mild intestinal epithelial injuries and apoptosis along with decreased ileal and systematic inflammation ($p < 0.05$) (Figure 7F-H).

Lysozyme restored intestinal barrier integrity and protected against bacterial translocation. The expression of the TJPs (claudin1, occludin, ZO-1) was increased following lysozyme administration in Dith+AP mice (Figure S4D-G). The mRNA expression of Wnt3a, TGFβ, and Lgr5 and protein expression of Lgr5 were restored in Lyz+Dith+AP mice (Figure 8A-B). Moreover, lysozyme supplementation restored the proliferation of IECs, which was suppressed in Dith+AP mice, as determined by PCNA staining (Figure 8C).

Lyz+Dith+AP mice presented lower intestinal permeability than Dith+AP mice, based on measurements of serum levels of D-lactate, DAO and endotoxin ($p < 0.05$) (Figure 8D-F). The FISH analysis confirmed a decreased number of bacteria within the intestinal mucosa and the pancreas of Lyz+Dith+AP mice versus Dith+AP mice (Figure 8G-H). The number of anaerobes translocated to the liver and mesenteric lymph nodes also reduced significantly in Lyz+Dith+AP mice ($p < 0.05$) (Figure 8I-

J). These results suggested that pretreatment with lysozyme attenuated AP by reducing bacterial translocation and promoted mucosal repair by stimulating the proliferation of IECs.

Lysozyme regulated microbiota disorders induced by dysfunction of Paneth cells. We then evaluated the contribution of lysozyme-modulated microbiota in Dith+AP mice by fecal microbiota transplantation (FMT). ~~Fecal~~ The fecal microbiota of Lyz+Dith mice and Dith mice was colonized to ABX mice followed by induction of AP. ABX mice receiving feces from Lyz+Dith mice developed less severe pancreatic and ileal injuries ($p < 0.05$) compared with those receiving feces from Dith mice (Figure 9A-C). Alleviated pancreatic and ileal inflammation ~~were~~ was evidenced by reduced proinflammatory cytokines ($p < 0.05$) by real time PCR in ABX mice receiving FMT from Lyz+Dith mice compared with those receiving FMT from Dith mice (Figure 9D-E). Therefore, lysozyme markedly reduced the severity of AP exacerbated in Dith+AP mice via regulating gut microbiota.

PCoA analysis showed that lysozyme gavage greatly shifted microbiota structure in Dith+AP mice (Figure 9F). Compared with Dith+AP mice, increased Chao1 index and ~~shannon~~ Shannon index ($p < 0.05$) and decreased ~~simpson~~ Simpson index ($p < 0.05$) showed increased alpha diversity of bacterial communities in lysozyme (Lyz) +Dith+AP mice (Figure 9G-I). At the phylum level, compared with Dith+AP mice, Lyz+Dith+AP mice presented increased relative abundance of *Firmicutes* ($p < 0.05$) and decreased relative abundance of *Bacteroidetes* and *Proteobacteria* ($p < 0.05$) and the normalized ratio of F/B ($p < 0.05$) (Figure 9J, S3B). At the genus level, supplementation of lysozyme restored the relative abundance of *Helicobacter* and *Blautia* in Dith+AP mice ($p < 0.05$) (Figure 9K). Therefore, lysozyme could restructure microbiota composition disrupted in Dith+AP mice.

We then carried out real time PCR for the four prevalent species of *Helicobacter. spp* and the three

prevalent species of *Blautia. spp* (20, 21). (Figure S3C). Compared with Con group, level of *Helicobacter felis* (*H. felis*), *Helicobacter hepaticus*(*H. hepaticus*) and *Blautia coccoides* (*B. coccoides*) increased significantly and level of *Blautia obeum* (*B. obeum*) decreased greatly in Dith group, level of *Helicobacter bilis* (*H. bilis*), *H. hepaticus* and *Bautia wexlerae* (*B. wexlerae*) increased significantly and level of *B. obeum* decreased greatly in AP group. Compared with AP group, the level of *H. bilis*, *H. felis*, *B. coccoides*, *B. obeum* and *B. wexlerae* decreased greatly and *H. hepaticus* increased significantly in Dith+AP group. Compared with Dith+AP group, level of *H. felis, B. obeum* and *B. wexlerae* increased greatly and the level of *H. hepaticus* and *B. coccoides* significantly decreased in Lyz+Dith+AP group. Although the association of *Helicobacter plyori* (*H. pylori*) with prolonged hospital stay in AP patients has been reported (22), *H. pylori* cannot be detected by real time PCR in the contents of the cecum (data not shown) in our research. The possible significance of specific species is discussed in the Discussion.

Lysozyme promoted enteroid proliferation through regulating functions of Paneth cell. We cultured enteroids to investigate IECs-lysozyme interactions based on the well-established technology of 3D culture. Compared with Con group, we observed that after 72 h intervention of lysozyme, both surface area and number of crypt buds per enteroid increased significantly ($p < 0.05$) (Figure 10A-C) (23). Lysozyme administration also promoted the proliferation of enteroids, as reflected by an increase in expression of PCNA, TJPs (occluding and claudin1), and Lgr5 ($p < 0.05$) (Figure 10D-F, S6B). Compared with Con group, Wnt3a and TGFfβ, along with β-catenin and c-myc, two crucial molecules of Wnt signaling pathway (24, 25), were significantly up-regulated ($p < 0.05$) in Lyz group (Figure 10F-H, S6B)). In addition, mRNA expression of AMPs (lysozyme, Defa5, Reg3γ, Ang4) were was also increased greatly ($p < 0.05$) in the Lyz group (Figure 10I, S4A-D).

LPS was utilized to imitate the inflammatory microenvironment in enteroid system (26). In Lyz+LPS group, the release of proinflammatory factors was decreased (p < 0.05) (Figure 10J), while cell proliferation and the integrity of the intercellular TJPs which were disrupted in the LPS group were protected by lysozyme (Figure 10D-H, S6B). To note, lysozyme failed to reverse the reduction of AMPs of Paneth cells induced by LPS (Figure 10I, S5A-D).

Discussion

In this study, we first confirmed the dysfunction of Paneth cell in AP patients. Our in vivo experiments showed that long-term reduction of Paneth cells exacerbated injuries and inflammation in the pancreas and small intestine in AP mice. Dith+AP mice also presented with increased intestinal permeability, bacteria translocation, and intestinal microbiota disorder compared with AP mice. Such changes were significantly reversed by lysozyme treatment. Functional recovery of Paneth cells might be a novel target for the treatment of intestinal dysfunction during AP.

Paneth cells constitute part of innate immunity by secreting various antimicrobial peptides. ~~High~~ A high concentration of antimicrobial peptides in crypts constructs a relatively sterile environment and prevents pathogen invasion (27). The involvement of Paneth cell dysfunction in the pathogenesis of multiple ~~deseases~~ diseases has been widely reported, such as Crohn's disease (CD) (11), alcoholic steatohepatitis (28), graft-versus-host disease (GVHD) (29), irritable bowel syndrome (IBS) (15), etc. The dysfunction of Paneth cell in AP patients and AP mice, manifested by a marked reduction of number of Paneth cells and expression of AMPs, was in line with previous findings in rats (30). Our previous study demonstrated that acute ablation of Paneth cells using dithizone aggravated the severity of rat AP, but the mechanism of a protective role of Paneth cells in AP remains unclear (14). Therefore, we established a

model with a long-term reduction of Paneth cells. Inflammation and damage were significantly increased in the pancreas and small intestine of Dith+AP mice compared with that of AP mice.

AP is often accompanied by intestinal barrier dysfunction, and the translocation of bacteria derived from the small intestine exacerbates systemic inflammation (31). Intestinal dysfunction in AP is thought to be associated with ischemia-reperfusion damage, severe oxidative stress, and apoptosis in the intestinal mucosa (32, 33). In recent years, Paneth cells have been proved to regulate the proliferation of intestinal stem cells and maintain the dynamic balance of intestinal epithelial cells by secreting support factors (10). *Bifidobacterium longum* promotes cell proliferation and expression of Lgr5 and wnt3a in intestinal organoids by regulating functions of the Paneth cell (34). $VDR^{\Delta PC}$ mice exhibited abnormal Paneth cells and decreased expression of PCNA and β-catenin (35). Our study found that compared with AP mice, Dith+AP mice exhibited markedly decreased TJPs expression, increased intestinal permeability and bacteria translocation, Meanwhile, expression of Lgr5, Wnt3a, TGFβ and intestinal epithelial proliferation were also significantly reduced. These findings suggested that Paneth cell dysfunction resulting in its diminished support for intestinal stem cells was part of the reasons for increased intestinal permeability during AP.

Intestinal microbiota disorders are common in patients with moderate or severe AP and are significantly related to the severity of inflammation, indicating that intestinal microbiota is involved in the progression of AP (5). Administration of *Escherichia coli* MG1655 in AP rats aggravated injuries in the pancreas and small intestine and activated TLR4/MyD88/MAPK and endoplasmic reticulum stress in intestinal epithelial cells, while *Parabacteroides* produces acetate to alleviate heparanase-exacerbated AP through reducing neutrophil infiltration(36, 37). The inflammation was significantly reduced in ABX mice

with AP further proved the role of gut microbiota in AP (36, 38). Paneth cells protect the host from intestinal pathogens and shape the composition of the colonized microbiota. FMT proved that gut microbiota disturbance caused by long-term reduction of Paneth cells played an important role in AP aggravation. 16S rRNA sequencing of cecal content revealed that long-term reduction of Paneth cells altered gut microbiota structure, decreased richness and diversity, increased the relative abundance of deleterious bacteria *Helicobacter* and decreased the relative abundance of beneficial bacteria *Blautia*.

Supplementing products of Paneth cell as functional recovery is a commonly used method in studies related to Paneth cell dysfunction. HD5 supplementation effectively altered gut microbiota in alcoholic hepatitis and reversed alcohol-induced damage (28). Lysozyme supplementation prevented *Escherichia coli* expansion and visceral hypersensitivity during maternal isolation (15). ANG1 treatment prevented dysbiosis in mice and alleviated DSS-induced colitis (39). Overexpression of Reg3γ protected mice from alcoholic hepatitis and reduced bacterial translocation (40). Supplement of lysozyme was the most appropriate as a functional recovery of Paneth cells in our study. Pretreatment of lysozyme restored gut microbiota disturbance, reduced relative abundance of *Helicobacter*, restored relative abundance of *Blautia, and* reversed aggravated ileal and pancreatic injuries in Dith+AP mice. FMT further confirmed that Paneth cells played a protective role in AP by stabilizing the intestinal microbiota.

Lysozyme is a cornerstone of innate immunity. Previous in vitro studies showed that lysozyme is a non-specific antimicrobial peptide, while different bacteria possessed distinct sensitivity to lysozyme (41-44). Although in our research *H. pylori* cannot be detected by real time PCR, studies have shown that *H. pylori* positive patients exhibited a higher relative abundance of *Proteobacteria* (45). The changes ~~of~~ in *H. hepaticus* and *B. obeum* are of concern and warrant~~s~~ further research (Figure S3C). Studies

demonstrated that cytolethal distending toxin subunit B (CdtB) produced by *H. hepaticus* exacerbated the severity of colitis via inducing inflammatory response and activating the Jak-Stat signaling pathway (46, 47). *B. obeum* generating bile salt hydrolases (BSH) could inhibit~~ing~~ the growth and colonization of *Vibrio cholerae* and *Clostridioides difficile* (48, 49). These researches suggested that changes of *H. hepaticus* and *B. obeum* might be involved in progression of AP. The role of species altered by Paneth cells depletion or lysozyme supplement in AP required further investigation.

In addition to the antimicrobial effect, lysozyme modulated innate immunity. The sensing of lysozyme-mediated production of pathogen-associated molecular patterns (PAMP) by pattern recognition receptor (PRR) stimulated downstream proinflammatory signaling and the production of proinflammatory cytokines (50, 51). Lysozyme could also limit intestinal inflammation. Zhang et al showed that intestinal inflammation was associated with the failure of secretion of Paneth cell lysozyme in mouse model of Crohn's disease (52, 53). Furthermore, lysozyme supplements could ameliorate intestinal inflammation of porcine colitis (54). Mechanisms of lysozyme limiting intestinal inflammation ~~was~~ were still unclear with speculation of limited bacterial invasion and activated protective intestinal immune response.

Organoid techniques have become a powerful tool for studying intestinal epithelium in vitro (55, 56). Lysozyme intervention promoted the growth of organoids, activated the Wnt pathway, and promoted epithelial proliferation. Therefore, lysozyme secreted by Paneth cells not only maintains microbiota homeostasis~~,~~ but also promotes proliferation of the intestinal organoids. Studies designed to explore possible mechanisms should be further performed.

We first used the method of multiple intraperitoneal injections of dithizone to maintain Paneth cells at a low level, but this method still had limitations. The protective effect of functional recovery of Paneth

cells in AP mice provides new strategies for clinical treatment of intestinal complications during AP.

Materials and Methods

Human intestinal biopsies. After obtaining written informed consent, human intestinal biopsies from the descending part of the duodenum of 21 patients with AP and 14 healthy controls were obtained upon endoscopy from the department of gastroenterology of Shanghai General Hospital, excluding individuals with diseases affecting Paneth cells, including irritable bowel syndrome, inflammatory bowel disease, alcoholic liver disease, etc. There was no statistical difference in baseline demographic and clinical characteristics between AP patients and healthy controls (See Table S1). Biopsies were stored in 4% Paraformaldehyde or liquid nitrogen.

Animals. Male C57BL/6 mice (6-8 weeks, 20-25 g) were obtained from Shanghai SLAC Laboratory Animal Co. Mice were housed under specific pathogen-free (SPF) conditions with a room temperature of 24±2°C and a 12 h light/dark cycle.

Experimental design. Mice were randomly divided into 5 groups (n = 6) : control (Con) group, dithizone (Dith) group, AP group, Dith+AP group and lysozyme (Lyz) treated (Lyz+Dith+AP) group. Mice in Dith group, Dith+AP group and Lyz+Dith+AP group were intraperitoneally injected with 40 mg/kg dithizone (Sigma-Aldrich, USA) every three days for two weeks. The mice in Lyz+Dith+AP group received oral gavage of 200 U/day lysozyme (Sigma-Aldrich, USA) for two weeks. Mice in the Con group and AP group were intraperitoneally injected with normal saline (NS). After treatment of dithizone or NS, mice in AP group, Dith+AP group and Lyz+Dith+AP group were injected intraperitoneally twice with 4.5 g/kg L-arginine (Sigma-Aldrich, USA). Mice in Con group and Dith group were intraperitoneally injected with normal saline (NS). Mice in Cae+AP group were injected intraperitoneally with 100 μg/kg caerulein

(MedChemExpress, CN) ten times with an hour interval between consecutive injections. After the final injection, 5 mg/kg Lipopolysaccharide (Sigma-Aldrich, USA) was intraperitoneally injected. Mice in N+AP group were induced AP as previously described (57). 2 % sodium taurocholate (Sigma-Aldrich, USA) solution at a volume of 50 μl /20 g bodyweight was infused into the biliopancreatic duct at the speed of 5 μl/min. Mice were anesthetized with Zoletil50 were used to anesthetize and then sacrificed at 72 h after the first injection of L-arginine. Blood samples, distal ileum, pancreas, liver, and mesenteric lymph nodes were collected, and stored at -80 °C or 4% paraformaldehyde. Fresh contents in ileocecum were also collected for analysis of gut microbiota.

Histological analysis. Fresh pancreas and distal ileum were soaked in 4% paraformaldehyde and dehydrated. Tissues were then embedded in paraffin and cut into sections of 4 μm. Sections were stained with hematoxylin and eosin (H&E, Servicebio, China) as previously described (58). Histopathological ~~injury~~ injuries were examined by a light microscope (Leica, Germany). ~~Pancreatic~~ The pancreatic injury was assessed according to scoring criteria reported by Schmidt et al (59), while distal ileal injury was evaluated as described by Chiu et al (60). Paneth cells were counted as previously reported (61).

Real time PCR. Tissue total RNA was extracted using TRIzol (Invitrogen, USA) and Tissus RNA Purification Kit Plus (EZBioscience, USA). Complementary DNA (cDNA) synthesis was performed using HyperScript III RT SuperMix (EnzyArtisan, China) for qPCR with gDNA Remover. Bacterial DNA was extracted from fecal samples with E.Z.N.A. Stool DNA Kit (Omega, USA) . The concentration of RNA or DNA was detected by NanoDrop2000 (Thermo Scientific, USA). 2x S6 Universal SYBR qPCR Mix (EnzyArtisan, China) was used to perform real time PCR with QuantStudio 6 Flex Realtime PCR Systems (Thermo Scientific, USA) following this protocol: predenaturation (95 °C, 30 s), 40 amplification cycles

of denaturation (95 °C, 10 s) and annealing and extension (60 °C, 30 s). Gene expression was measured by $2^{-\Delta\Delta Ct}$ method. Primers used for detection were provided in Table S2.

Pancreas wet weight to dry weight (W/D) ratio and serum amylase assays. The pancreatic tissue was weighed and then incubated at 80 °C for 48 h to obtain a constant weight as the dry weight. The ratio of the wet pancreas weight to the dry pancreas weight was calculated to evaluate tissue edema. The level of serum amylase was detected by Amylase Reagents using ADVIA 2400 Chemistry System (SIEMENS, German) according to technicians' instructions.

Immunofluorescence. Distal ileal sections were heated at 60 °C for 1 h. Then, sections were soaked into differents jars (xylene 40 min, 100% ethanol 10 min, 95% ethanol 10 min, 80% ethanol 5 min, 70% ethanol 5 min, doubly-distilled water 3 min) to deparaffinized and rehydrate. Antigens were retrieved with citrate antigen retrieval solution (Sangon Biotech, CN). After repeatedly washing in phosphate-buffered saline (PBS), super pap pen (Sangon Biotech, CN) was used to draw a circle around tissue. Slides were blocked with immunostaining blocking buffer (Sangon Biotech, CN) at room temperature for 1 h, and incubated with primary antibody against PCNA (A0264, Abclonal, CN), occludin (A2601, Abclonal, CN), claudin-1 (ab211737, Abcam, USA), ZO-1 (13663, Cell Signaling Technology, USA) diluted by primary antibody dilution buffer (Sangon Biotech, CN) at 4 °C overnight. Slides were washed with PBS and incubated with Alexa Fluor 488 AffiniPure Donkey anti-Rabbit IgG (Yeason, CN) for 1 h at room temperature. Then, the slides were washed with PBS and stained with dihydrochloride (Yeason, CN) for 10 min. Images were captured with a fluorescence microscope (Leica, USA).

Western blot. Distal ileum tissues were lysed in RIPA lysis buffer (Epizyme Biotech, CN) with 1% protease inhibitor (Epizyme Biotech, CN) and fully ground using a high-throughput tissue grinder (Onebio.

Biotech, CN). The suspension was left to settle on ice for 1 h and centrifuged at 10000 g for 10 min at 4 °C. After taking the supernatant and mixing with the SDS-loading buffer (Yeason, CN), the mixed solution was heated at 100°C for 10 min. 10 μl solution was loaded into a 10% SDS-PAGE gel produced by PAGE Gel Fast Preparation Kit (Epizyme Biotech, CN) for electrophoresis. Then proteins in the gel were transferred to 0.2 μm PVDF membrane (Millipore, USA). The membrane was blocked with 3% bovine serum albumin (BSA) for 1 h and incubated with primary antibodies diluted by primary antibody dilution buffer (Epizyme Biotech, CN) against Lgr5 (A10545, Abclonal, CN), lysozyme (A0099, Dako, Danmark), Reg3γ (sc-377038, Santa Cruz Biotechnology, USA), Defa5 (A18208, Abclonal, CN), Ang4 (sc-377497, Santa Cruz Biotechnology, USA), sPLA2 (sc-58363, Santa Cruz Biotechnology, USA) overnight at 4 °C. ~~The~~ On the second day, the membrane was washed 3 times with Tris-buffered saline with Tween-20 (TBST) buffer and incubated with Peroxidase-Conjugated Goat Anti-Rabbit IgG (H+L) (Yeason, CN) for 60 min at room temperature. ~~Membrane~~ The membrane was washed 3 times with TBST again. Bands were visualized with HRP Substrate Peroxide Solution (Millipore, USA) by Amersham Imager 600 (General Electric, USA).

Enzyme-linked immunosorbent assay (ELISA). The levels of IL-1β, TNF-α, and IL-6 in the serum, pancreas and ileum were detected by Luminex Mouse Discovery Assay Kit (R&D Systems, USA)according to the instructions. The levels of pancreatic MPO, serum endotoxin, DAO and D-Lactate were measured using MPO mice ELISA kit, endotoxin mice ELISA kit, DAO mice ELISA kit and D-Lactate mice ELISA kit (MultiSciences Biotech, CN) according to provided protocols.

TUNEL and FISH assay. Apoptosi~~s~~s was evaluated by Tunel assay using Fluorescein Tunel Cell Apoptosis Detection Kit (Servicebio, CN) according to the instructions. Pancreatic and ileal Tunel positive

cell counting ~~were~~ was performed at ×200 magnification. Fluorescence in situ hybridization (FISH) was used to detect bacterial translocation as previously described. In short, sections of the distal ileum and pancreas were heated 60 min and dewaxed (2×10 min with 100% xylene, 5 min with 100% ethanol). Next, sections were incubated with specific probes (EUB338: 5'-Cy3-GCTGCCTCCCGTAGGAGT-3') in a wet box at 52 °C for 18 h. Then, sections were washed and stained with DAPI. Images were captured with a fluorescence microscope (Leica, USA).

Bacterial cultures and plate counting. Mesenteric lymph nodes and liver tissues were collected in sterile PBS, fully ground using a high-throughput tissue grinder (Onebio. Biotech, CN), and plated onto brain heart infusion agar plates for a culture of anaerobic bacteria. The plates were incubated for 48 h at 37 °C using Oxoid AnaeroGen 2.5 L and Oxoid Resazurin Anaerobic Indicator (Thermo Scientific, USA). The plates producing 25 to 250 colony-forming units (CFUs) were counted.

Fecal microbiota transplantation (FMT). Feces ~~was~~ were collected from Con mice, Dith mice, and Dith+Lyz mice. The processing of FMT suspension was done within 2 hours. 100 mg feces was resuspended in 1 mL saline and centrifuged for 5 min. The supernatant was used as FMT suspension. The mice receiving antibiotics were treated with vancomycin (0.5 mg/mL), neomycin (1 mg/mL), ampicillin (1 mg/mL), and metronidazole (1 mg/mL) (Sangon Biotech, CN) in their drinking water for 4 weeks. Mice were divided into four groups: Con group, ABX+Con group, ABX+Dith group and ABX+Dith+Lyz group. Con group received no treatment. ABX+Con group ~~were~~ was gavaged with feces from control mouse for 1 week~~s~~, ABX+Dith group ~~were~~ was gavaged with 200μL FMT suspension from Dith mice for 1 week~~s~~, and ABX+Dith+Lyz group was gavaged with 200μL FMT suspension from Dith+Lyz mice for 1 week~~s~~. AP was induced in mice from ABX+Con group, ABX+Dith group and ABX+Dith+Lyz group.

16S rRNA sequencing. Genomic DNA was extracted from the contents of the ileocecum using E.Z.N.A. Stool DNA Kit (Omega, USA) according to the manufacturer's instructions and amplified using forward (5'- TACGGRAGGCAGCAG -3') and reverse (5'- AGGGTATCTAATCCT -3'). 16S rDNA high-throughput sequencing was performed on an Illumina HiSeq platform (Illumina, USA) according to the standard protocols by the Majorbio Bio-Pharm Technology. The raw sequencing reads of this study are openly available in BioProject at https://www.ncbi.nlm.nih.gov/bioproject/PRJNA774193, reference number PRJNA774193.

Microbiome analysis. The sequences were filtered with fastp (0.19.6) and merged with FLASH (v1.2.11). Then the high-quality sequences were denoised using DADA2 plugin in the QIIME2 (version 2020.2) pipeline with recommended parameters, which are called amplicon sequence variants (ASVs). Taxonomic assignment of ASVs was performed using the Naive bayes consensus taxonomy classifier implemented in QIIME2 and the SILVA 16S rRNA database (v138). Analysis of the gut microbiota was carried out using the Majorbio Cloud platform (https://cloud.majorbio.com). Alpha diversity indices including Chao1 richness, Shannon index and Simpson index were calculated with Mothur v1.30.1. Principal coordinate analysis (PCoA) based on Bray-curtis dissimilarity using Vegan v2.5-3 package was performed to analyse the microbial communities in different samples. The linear discriminant analysis (LDA) effect size (LEfSe) was performed to identify the significantly abundant genera of bacteria among the different groups (LDA score > 2, $P < 0.05$). Correlations between histopathological score, serum D-lactate and DAO, serum inflammation factors, endotoxin and the relative abundance of diffferent genera were calculated using Spearman's analysis.

Enteroids establishment and co-culture with lysozyme. Enteroids were obtained from C57BL/6 mice.

The distal 10 cm of the small intestine was collected and flushed gently with ice-cold PBS for 5 min. Then the intestine were was cut open along their longitudinal axis and cut to 2 mm segments. Crypts were isolated from the segments by incubating in 2 mM EDTA for 30 min, and then in 5 mM EDTA for 30 min. After 5 min resting, the supernatant was removed. Crypts were resuspended in 15 ml DMEM F12 (Wisent, China) with repeatedly blowing. Then the suspension was filtered through a 70 μm filter mesh (BD Biosciences, USA) and centrifuged at 300 g for 5 min. After the supernatant was discarded, the pellet was mixed with Matrigel (Corning, USA) and DMEM F12 in 1:1 ratio. Then 50 μL suspension was planted into each well of the pre-warmed 24 well plates and 700 μL of IntestiCult™ Organoid Growth Medium (Mouse) (Stemcell Technologies, Canada) was added per well. In the following culture, the half medium was replaced every 3 day, and passaged every 9 days. 200 U lysozyme was added into enteroids per well and incubated at 37 °C and 5% CO2 for 72 h. 1 mg/mL LPS with 200 U lysozyme was added into enteroids per well and incubated for 24 h. Total RNA was extracted using EZ-press RNA Purification Kit (EZBioscience, USA). The methods of real time PCR, western blot and immunofluorescence of enteroids are the same as above.

Statistical analysis. Data was exhibited in the form of mean ± standard deviation (SD). Comparisons between two groups with a normal distribution were performed by t-test. Spearman's rank correlation coefficient was used to detect correlations between bacterial genus and indicators. One-way ANOVA was performed for three or more groups . Differences in the male/female ratio and BMI rates between groups were tested by the Chi-square test. All the statistical analyses were carried out in IBM SPSS Statistics 25. $P < 0.05$ suggested a statistically significant difference.

Study approval. All studies involving human samples were approved by the Ethics Committee of

Shanghai General Hospital (2021035) and registered in Chinese Clinical Trial Registry (ChiCTR1800017214). All the animal experiments were approved by Institutional Animal Care and Use Committee (IACUC) (2020AW095) and conducted according to the instructions of IACUC.

Author contributions

YF and YZ designed the studies; YF, QM and NY performed experiments and acquired data; BL and SL obtained human intestinal biopsies. ZH and BX analyzed and interpreted the data; YF drafted the manuscript; JF, CH and YZ revised the manuscript critically with important intellectual contents. All authors approved the final version of the manuscript

Acknowledgements

The authors have declared that no conflict of interest exists.. This work was financially supported by the Clinical Research Plan of SHDC (SHDC2020CR2014A) and the National Natural Science Foundation of China (81970555).

References

1. Peery AF, Crockett SD, Murphy CC, Lund JL, Dellon ES, Williams JL, Jensen ET, Shaheen NJ, Barritt AS, Lieber SR, Kochar B, Barnes EL, Fan YC, Pate V, Galanko J, Baron TH, Sandler RS. 2019. Burden and Cost of Gastrointestinal, Liver, and Pancreatic Diseases in the United States: Update 2018. Gastroenterology 156:254-272.e11.
2. Boxhoorn L, Voermans RP, Bouwense SA, Bruno MJ, Verdonk RC, Boermeester MA, van Santvoort HC, Besselink MG. 2020. Acute pancreatitis. Lancet 396:726-734.
3. Fritz S, Hackert T, Hartwig W, Rossmanith F, Strobel O, Schneider L, Will-Schweiger K, Kommerell M, Büchler MW, Werner J. 2010. Bacterial translocation and infected pancreatic necrosis in acute necrotizing pancreatitis derives from small bowel rather than from colon. Am J Surg 200:111-7.
4. Van Felius ID, Akkermans LM, Bosscha K, Verheem A, Harmsen W, Visser MR, Gooszen HG. 2003. Interdigestive small bowel motility and duodenal bacterial overgrowth in experimental acute pancreatitis. Neurogastroenterol Motil 15:267-76.
5. Tan C, Ling Z, Huang Y, Cao Y, Liu Q, Cai T, Yuan H, Liu C, Li Y, Xu K. 2015. Dysbiosis of Intestinal Microbiota Associated With Inflammation Involved in the Progression of Acute Pancreatitis. Pancreas 44:868-75.

6. Liu Z, Shen Y, Cui N, Yang J. 2011. Clinical observation of immunity for severe acute pancreatitis. Inflammation 34:426-31.

7. Porter EM, Bevins CL, Ghosh D, Ganz T. 2002. The multifaceted Paneth cell. Cell Mol Life Sci 59:156-70.

8. Salzman NH, Hung K, Haribhai D, Chu H, Karlsson-Sjöberg J, Amir E, Teggatz P, Barman M, Hayward M, Eastwood D, Stoel M, Zhou Y, Sodergren E, Weinstock GM, Bevins CL, Williams CB, Bos NA. 2010. Enteric defensins are essential regulators of intestinal microbial ecology. Nat Immunol 11:76-83.

9. Bevins CL, Salzman NH. 2011. Paneth cells, antimicrobial peptides and maintenance of intestinal homeostasis. Nat Rev Microbiol 9:356-68.

10. Sato T, van Es JH, Snippert HJ, Stange DE, Vries RG, van den Born M, Barker N, Shroyer NF, van de Wetering M, Clevers H. 2011. Paneth cells constitute the niche for Lgr5 stem cells in intestinal crypts. Nature 469:415-8.

11. Cadwell K, Liu JY, Brown SL, Miyoshi H, Loh J, Lennerz JK, Kishi C, Kc W, Carrero JA, Hunt S, Stone CD, Brunt EM, Xavier RJ, Sleckman BP, Li E, Mizushima N, Stappenbeck TS, Virgin HWt. 2008. A key role for autophagy and the autophagy gene Atg16l1 in mouse and human intestinal Paneth cells. Nature 456:259-63.

12. Coutinho HB, da Mota HC, Coutinho VB, Robalinho TI, Furtado AF, Walker E, King G, Mahida YR, Sewell HF, Wakelin D. 1998. Absence of lysozyme (muramidase) in the intestinal Paneth cells of newborn infants with necrotising enterocolitis. J Clin Pathol 51:512-4.

13. Estienne M, Claustre J, Clain-Gardechaux G, Paquet A, Taché Y, Fioramonti J, Plaisancié P. 2010. Maternal deprivation alters epithelial secretory cell lineages in rat duodenum: role of CRF-related peptides. Gut 59:744-51.

14. Guo Y, Huang C, Liu L, Fu X, Lu Y, Zheng J, Mei Q, Huang Z, Fan J, Lu L, Zeng Y. 2019. Paneth Cell Ablation Aggravates Pancreatic and Intestinal Injuries in a Rat Model of Acute Necrotizing Pancreatitis after Normal and High-Fat Diet. Mediators Inflamm 2019:8474523.

15. Riba A, Olier M, Lacroix-Lamandé S, Lencina C, Bacquié V, Harkat C, Gillet M, Baron M, Sommer C, Mallet V, Salvador-Cartier C, Laurent F, Théodorou V, Ménard S. 2017. Paneth Cell Defects Induce Microbiota Dysbiosis in Mice and Promote Visceral Hypersensitivity. Gastroenterology 153:1594-1606.e2.

16. Schoenborn AA, von Furstenberg RJ, Valsaraj S, Hussain FS, Stein M, Shanahan MT, Henning SJ, Gulati AS. 2019. The enteric microbiota regulates jejunal Paneth cell number and function without impacting intestinal stem cells. Gut Microbes 10:45-58.

17. Lucas López R, Grande Burgos MJ, Gálvez A, Pérez Pulido R. 2017. The human gastrointestinal tract and oral microbiota in inflammatory bowel disease: a state of the science review. Apmis 125:3-10.

18. Shin NR, Whon TW, Bae JW. 2015. Proteobacteria: microbial signature of dysbiosis in gut microbiota. Trends Biotechnol 33:496-503.

19. Hayase E, Hashimoto D, Nakamura K, Noizat C, Ogasawara R, Takahashi S, Ohigashi H, Yokoi Y, Sugimoto R, Matsuoka S, Ara T, Yokoyama E, Yamakawa T, Ebata K, Kondo T, Hiramine R, Aizawa T, Ogura Y, Hayashi T, Mori H, Kurokawa K, Tomizuka K, Ayabe T, Teshima T. 2017. R-Spondin1 expands Paneth cells and prevents dysbiosis induced by graft-versus-host disease. J Exp Med 214:3507-3518.

20. Smet A, Menard A. 2020. Review: Other Helicobacter species. Helicobacter 25 Suppl 1:e12744.

21. Liu X, Mao B, Gu J, Wu J, Cui S, Wang G, Zhao J, Zhang H, Chen W. 2021. Blautia-a new functional genus with potential probiotic properties? Gut Microbes 13:1-21.

22. Khan J, Pelli H, Lappalainen-Lehto R, Järvinen S, Sand J, Nordback I. 2009. Helicobacter pylori in alcohol induced acute pancreatitis. Scand J Surg 98:221-4.

23. Fuller MK, Faulk DM, Sundaram N, Shroyer NF, Henning SJ, Helmrath MA. 2012. Intestinal crypts reproducibly expand

in culture. J Surg Res 178:48-54.
24. Katoh M, Katoh M. 2007. WNT signaling pathway and stem cell signaling network. Clin Cancer Res 13:4042-5.
25. Dang CV. 2012. MYC on the path to cancer. Cell 149:22-35.
26. Günther C, Buchen B, He GW, Hornef M, Torow N, Neumann H, Wittkopf N, Martini E, Basic M, Bleich A, Watson AJ, Neurath MF, Becker C. 2015. Caspase-8 controls the gut response to microbial challenges by Tnf-α-dependent and independent pathways. Gut 64:601-10.
27. Salzman NH, Bevins CL. 2013. Dysbiosis--a consequence of Paneth cell dysfunction. Semin Immunol 25:334-41.
28. Zhong W, Wei X, Hao L, Lin TD, Yue R, Sun X, Guo W, Dong H, Li T, Ahmadi AR, Sun Z, Zhang Q, Zhao J, Zhou Z. 2020. Paneth Cell Dysfunction Mediates Alcohol-related Steatohepatitis Through Promoting Bacterial Translocation in Mice: Role of Zinc Deficiency. Hepatology 71:1575-1591.
29. Eriguchi Y, Takashima S, Oka H, Shimoji S, Nakamura K, Uryu H, Shimoda S, Iwasaki H, Shimono N, Ayabe T, Akashi K, Teshima T. 2012. Graft-versus-host disease disrupts intestinal microbial ecology by inhibiting Paneth cell production of α-defensins. Blood 120:223-31.
30. Chen J, Huang C, Wang J, Zhou H, Lu Y, Lou L, Zheng J, Tian L, Wang X, Cao Z, Zeng Y. 2017. Dysbiosis of intestinal microbiota and decrease in paneth cell antimicrobial peptide level during acute necrotizing pancreatitis in rats. PLoS One 12:e0176583.
31. Liu J, Huang L, Luo M, Xia X. 2019. Bacterial translocation in acute pancreatitis. Crit Rev Microbiol 45:539-547.
32. Camargo EA, Santana DG, Silva CI, Teixeira SA, Toyama MH, Cotrim C, Landucci EC, Antunes E, Muscara MN, Costa SK. 2014. Inhibition of inducible nitric oxide synthase-derived nitric oxide as a therapeutical target for acute pancreatitis induced by secretory phospholipase A2. Eur J Pain 18:691-700.
33. Tian R, Tan JT, Wang RL, Xie H, Qian YB, Yu KL. 2013. The role of intestinal mucosa oxidative stress in gut barrier dysfunction of severe acute pancreatitis. Eur Rev Med Pharmacol Sci 17:349-55.
34. Zhou C, Fang X, Xu J, Gao J, Zhang L, Zhao J, Meng Y, Zhou W, Han X, Bai Y, Li Z, Zou D. 2020. Bifidobacterium longum alleviates irritable bowel syndrome-related visceral hypersensitivity and microbiota dysbiosis via Paneth cell regulation. Gut Microbes 12:1782156.
35. Lu R, Zhang YG, Xia Y, Zhang J, Kaser A, Blumberg R, Sun J. 2021. Paneth Cell Alertness to Pathogens Maintained by Vitamin D Receptors. Gastroenterology 160:1269-1283.
36. Zheng J, Lou L, Fan J, Huang C, Mei Q, Wu J, Guo Y, Lu Y, Wang X, Zeng Y. 2019. Commensal Escherichia coli Aggravates Acute Necrotizing Pancreatitis through Targeting of Intestinal Epithelial Cells. Appl Environ Microbiol 85.
37. Lei Y, Tang L, Liu S, Hu S, Wu L, Liu Y, Yang M, Huang S, Tang X, Tang T, Zhao X, Vlodavsky I, Zeng S, Tang B, Yang S. 2021. Parabacteroides produces acetate to alleviate heparanase-exacerbated acute pancreatitis through reducing neutrophil infiltration. Microbiome 9:115.
38. Li X, He C, Li N, Ding L, Chen H, Wan J, Yang X, Xia L, He W, Xiong H, Shu X, Zhu Y, Lu N. 2020. The interplay between the gut microbiota and NLRP3 activation affects the severity of acute pancreatitis in mice. Gut Microbes 11:1774-1789.
39. Sun D, Bai R, Zhou W, Yao Z, Liu Y, Tang S, Ge X, Luo L, Luo C, Hu GF, Sheng J, Xu Z. 2021. Angiogenin maintains gut microbe homeostasis by balancing α-Proteobacteria and Lachnospiraceae. Gut 70:666-676.
40. Wang L, Fouts DE, Stärkel P, Hartmann P, Chen P, Llorente C, DePew J, Moncera K, Ho SB, Brenner DA, Hooper LV, Schnabl B. 2016. Intestinal REG3 Lectins Protect against Alcoholic Steatohepatitis by Reducing Mucosa-Associated Microbiota and Preventing Bacterial Translocation. Cell Host Microbe 19:227-39.
41. Laible NJ, Germaine GR. 1985. Bactericidal activity of human lysozyme, muramidase-inactive lysozyme, and cationic

polypeptides against Streptococcus sanguis and Streptococcus faecalis: inhibition by chitin oligosaccharides. Infect Immun 48:720-8.

42. Ellison RT, 3rd, Giehl TJ. 1991. Killing of gram-negative bacteria by lactoferrin and lysozyme. J Clin Invest 88:1080-91.

43. Ragland SA, Criss AK. 2017. From bacterial killing to immune modulation: Recent insights into the functions of lysozyme. PLoS Pathog 13:e1006512.

44. Yu S, Balasubramanian I, Laubitz D, Tong K, Bandyopadhyay S, Lin X, Flores J, Singh R, Liu Y, Macazana C, Zhao Y, Béguet-Crespel F, Patil K, Midura-Kiela MT, Wang D, Yap GS, Ferraris RP, Wei Z, Bonder EM, Häggblom MM, Zhang L, Douard V, Verzi MP, Cadwell K, Kiela PR, Gao N. 2020. Paneth Cell-Derived Lysozyme Defines the Composition of Mucolytic Microbiota and the Inflammatory Tone of the Intestine. Immunity 53:398-416.e8.

45. Gao JJ, Zhang Y, Gerhard M, Mejias-Luque R, Zhang L, Vieth M, Ma JL, Bajbouj M, Suchanek S, Liu WD, Ulm K, Quante M, Li ZX, Zhou T, Schmid R, Classen M, Li WQ, You WC, Pan KF. 2018. Association Between Gut Microbiota and Helicobacter pylori-Related Gastric Lesions in a High-Risk Population of Gastric Cancer. Front Cell Infect Microbiol 8:202.

46. Corbin AL, Gomez-Vazquez M, Berthold DL, Attar M, Arnold IC, Powrie FM, Sansom SN, Udalova IA. 2020. IRF5 guides monocytes toward an inflammatory CD11c(+) macrophage phenotype and promotes intestinal inflammation. Sci Immunol 5.

47. Zhu L, Zhu C, Cao S, Zhang Q. 2021. Helicobacter hepaticus Induce Colitis in Male IL-10(-/-) Mice Dependent by Cytolethal Distending Toxin B and via the Activation of Jak/Stat Signaling Pathway. Front Cell Infect Microbiol 11:616218.

48. Alavi S, Mitchell JD, Cho JY, Liu R, Macbeth JC, Hsiao A. 2020. Interpersonal Gut Microbiome Variation Drives Susceptibility and Resistance to Cholera Infection. Cell 181:1533-1546.e13.

49. Mullish BH, McDonald JAK, Pechlivanis A, Allegretti JR, Kao D, Barker GF, Kapila D, Petrof EO, Joyce SA, Gahan CGM, Glegola-Madejska I, Williams HRT, Holmes E, Clarke TB, Thursz MR, Marchesi JR. 2019. Microbial bile salt hydrolases mediate the efficacy of faecal microbiota transplant in the treatment of recurrent Clostridioides difficile infection. Gut 68:1791-1800.

50. Wolf AJ, Arruda A, Reyes CN, Kaplan AT, Shimada T, Shimada K, Arditi M, Liu G, Underhill DM. 2011. Phagosomal degradation increases TLR access to bacterial ligands and enhances macrophage sensitivity to bacteria. J Immunol 187:6002-10.

51. Caruso R, Warner N, Inohara N, Núñez G. 2014. NOD1 and NOD2: signaling, host defense, and inflammatory disease. Immunity 41:898-908.

52. Zhang Q, Pan Y, Yan R, Zeng B, Wang H, Zhang X, Li W, Wei H, Liu Z. 2015. Commensal bacteria direct selective cargo sorting to promote symbiosis. Nat Immunol 16:918-26.

53. Wang H, Zhang X, Zuo Z, Zhang Q, Pan Y, Zeng B, Li W, Wei H, Liu Z. 2017. Rip2 Is Required for Nod2-Mediated Lysozyme Sorting in Paneth Cells. J Immunol 198:3729-3736.

54. Lee M, Kovacs-Nolan J, Yang C, Archbold T, Fan MZ, Mine Y. 2009. Hen egg lysozyme attenuates inflammation and modulates local gene expression in a porcine model of dextran sodium sulfate (DSS)-induced colitis. J Agric Food Chem 57:2233-40.

55. Tan SH, Phuah P, Tan LT, Yada S, Goh J, Tomaz LB, Chua M, Wong E, Lee B, Barker N. 2021. A constant pool of Lgr5(+) intestinal stem cells is required for intestinal homeostasis. Cell Rep 34:108633.

56. Wu H, Xie S, Miao J, Li Y, Wang Z, Wang M, Yu Q. 2020. Lactobacillus reuteri maintains intestinal epithelial regeneration and repairs damaged intestinal mucosa. Gut Microbes 11:997-1014.

57. Laukkarinen JM, Van Acker GJ, Weiss ER, Steer ML, Perides G. 2007. A mouse model of acute biliary pancreatitis induced by retrograde pancreatic duct infusion of Na-taurocholate. Gut 56:1590-8.

58. Mei QX, Hu JH, Huang ZH, Fan JJ, Huang CL, Lu YY, Wang XP, Zeng Y. 2021. Pretreatment with chitosan oligosaccharides attenuate experimental severe acute pancreatitis via inhibiting oxidative stress and modulating intestinal homeostasis. Acta Pharmacol Sin 42:942-953.

59. Shimizu T, Shiratori K, Sawada T, Kobayashi M, Hayashi N, Saotome H, Keith JC. 2000. Recombinant human interleukin-11 decreases severity of acute necrotizing pancreatitis in mice. Pancreas 21:134-40.

60. Chiu CJ, McArdle AH, Brown R, Scott HJ, Gurd FN. 1970. Intestinal mucosal lesion in low-flow states. I. A morphological, hemodynamic, and metabolic reappraisal. Arch Surg 101:478-83.

61. Liu L, Guo Y, Zheng J, Lu Y, Shen Y, Huang C, Zeng Y, Wang X. 2019. Paneth cell ablation increases the small intestinal injury during acute necrotizing pancreatitis in rats. Mol Med Rep 20:473-484.

Figure 1. AP patients and experimental AP mice presented Paneth cells defect. (A) Histopathological changes and mean number of Paneth cells per crypt of duodenal mucosa specimens were assessed by HE staining. Original magnification, 200× (n=7-14 individuals per group). (B) Lysozyme expression (green) was assessed in Paneth cells of duodenal mucosa specimens by immunofluorescence (200× magnification) and lysozyme+/DAPI+ quantification. The mRNA expression of (C) lysozyme, (D) HD6, (E) HD5, (F) Reg3γ, (G) Ang4, (H) sPLA2, (I) TGFβ, (J) Wnt3a and (K) Lgr5 were assessed. (L) Lysozyme expression (green) (200× magnification) and lysozyme+/DAPI+ quantification of three AP models. (M) Mean number of Paneth cells per crypt in AP models. The data are presented as the means ± SD; ns, no significant difference; * $p \leq 0.05$.

Figure 2. Long term reduction of Paneth cell aggravated AP-induced pancreatic and ileal injuries and

inflammation. (A) Pancreatic histopathological changes of AP mice with or without dithizone treatment. Original magnification, 100× (the upper figures) or 200× (the lower figures) (n=6 mice per group). (B) The mRNA expression of TNF-α, IL-6 and IL-1β. (C) Level of serum amylase. (D) Level of pancreatic edema. (E) Ileal histopathological changes. Original magnification, 200× (the upper figures) or 400× (the lower figures) (n=6 mice per group). (F-K) Ileal and serum levels of TNF-α, IL-6 and IL-1β. (L) Tunel staining of small intestines (200× magnification) and Tunel+/DAPI+ quantification. The data are presented as the means ± SD; ns, no significant difference; * $p \leq 0.05$.

Figure 3. Long term reduction of Paneth cell increased intestinal permeability. Images of ileal (A) claudin1, (B) ZO-1, (C) occludin immunofluorescence (200× magnification) and (D) corresponding cellular quantification (n=6 mice per group). Levels of (E) serum DAO and D-lactate. The mRNA expression of (F) Wnt3a, (G) TGFβ and (H) Lgr5. (I) Expression and quantification of intestinal Lgr5. (J) Immunofluorescence staining and quantification of PCNA (200× magnification). The data are presented as the means ± SD; ns, no significant difference; * $p \leq 0.05$.

Figure 4. Long term reduction of Paneth cell aggravated AP-induced bacterial translocation. (A) Levels of serum endotoxin. (B-E) Representative fluorescent photographs of pancreas (100× magnification) and ileum (200× magnification) and EUB338+/DAPI+ quantification. (n=6 mice per group). Colony-forming units (CFUs) were counted on anaerobic culture plates of (F) liver and (G) mesenteric lymph nodes (MLN). The data are presented as the means ± SD; ns, no significant difference; * $p \leq 0.05$.

Figure 5. The disturbance of intestinal microbiota was related to the exacerbation of AP. Representative images of histopathological changes in (A) pancreas, 100× (the upper figures) or 200× (the lower figures) and (B) ileum, 200× (the upper figures) or 400× (the lower figures) of ABX mice receiving FMT (n=6

mice per group). The mRNA expression of TNF-α, IL-6 and IL-1β in (C) pancreas and (D) ileum. (E) Principal coordinate analysis (PCoA) (n=4-6 mice per group) of the microbial communities. Alpha diversity, as revealed by (F) chao1 index, (G) ~~shannon~~ Shannon index and (H) ~~simpson~~ Simpson index was analyzed in Con and Dith mice with or without AP. (I, J) Relative abundance of top five phyla in Con and Dith mice with or without AP. The data are presented as the means ± SD; ns, no significant difference; * $p \leq 0.05$.

Figure 6. Changes of gut microbiota in genus level. (A) Linear discriminant analysis (LDA) scores at the genus level between Con and Dith mice. (B) LDA scores at the genus level between AP and Dith+AP mice. (C) Heatmap showed a correlation between intestinal barrier dysfunction, pathological changes, inflammation cytokines and gut microbiota. Blue means negative correlation, red means positive correlation. The data are presented as the means ± SD; ns, no significant difference; * $p \leq 0.05$.

Figure 7. Lysozyme ameliorated the severity of AP and prevented bacterial translocation. (A) Representative pancreatic sections after H&E staining. Original magnification, 100× (the upper figures) or 200× (the lower figures) (n=6 mice per group). (B) Level of serum amylase. (C) Tunel staining and Tunel+/DAPI+ quantification of apoptosis in the pancreas (100× magnification). (D) Level of pancreatic edema. (E) The level of TNF-α, IL-6 and IL-1β in the pancreas. (F) Representative ileal sections after H&E staining. Original magnification, 200× (the upper figures) or 400× (the lower figures). (G) Tunel staining and Tunel+/DAPI+ quantification of apoptosis in small intestines (200× magnification). (H) Ileal and serum levels of TNF-α, IL-6 and IL-1β. The data are presented as the means ± SD; ns, no significant difference; * $p \leq 0.05$.

Figure 8. Lysozyme prevented bacterial translocation. (A) The mRNA expression of TGFβ and Wnt3a. (B)

The mRNA and protein expression of Lgr5. (C) Immunofluorescence staining and quantification of PCNA (200× magnification). Levels of serum (D) endotoxin, (E) DAO and (F) D-lactate. FISH test of (G) pancreas (100× magnification) and (H) intestinal epithelium (200× magnification) using EUB338 probe. EUB338+/DAPI+ was quantified. CFUs were counted on anaerobic culture plates of (I) liver and (J) MLN. The data are presented as the means ± SD; ns, no significant difference; * $p \leq 0.05$.

Figure 9. Lysozyme restored microbiota disorders induced by dysfunction of Paneth cells. (A) Representative photographs of HE staining in pancreas of ABX mice receiving FMT (n=6 mice per group). (B) Level of amylase. (C) Representative photographs of HE staining in the ileum of ABX mice receiving FMT (n=6 mice per group). The mRNA expression of TNF-α, IL-6 and IL-1β in (D) pancreas and (E) ileum. (F) Principal coordinate analysis (PCoA) of bacterial ~~beta~~ beta-diversity (n=4-6 mice per group). (G-I) Alpha diversity analysis using chao1 index, ~~shannon~~ Shannon index and ~~simpson~~ Simpson index. (J) Relative abundance of top five phyla in Dith+AP mice and Lyz+Dith+AP mice. (K) LDA scores at the genus level between Dith+AP mice and Lyz+Dith+AP mice. The data are presented as the means ± SD; ns, no significant difference; * $p \leq 0.05$

Figure 10. Lysozyme promoted enteroid proliferation.(A) Light microscope photographs showed normal morphology and sizes of enteroids in Lyz group compared to that in Con group. (B) Mean surface area of enteroids was estimated at 1 day, 2 day, 3 day after incubating with lysozyme. (C) Crypt domains per enteroid were counted. (D) Representative images of fluorescence staining of PCNA (greeen) of enteroids after incubating with lysozyme and LPS. ~~Expression~~ The expression of PCNA was quantified. (E) The mRNA expression of Lgr5. (F) Image of western blot and protein quantification of Lgr5, occludin, claudin1, β-catenin, Wnt3a and c-myc. The mRNA expression of (G) Wnt3a, (H) TGFβ, β-catenin and c-

myc. (I) Representative images of fluorescence staining of lysozyme (greeen) of enteroids. Expression of lysozyme was quantified. (J) The mRNA expression of TNF-α, IL-6 and IL-1β of enteroids after incubating with lysozyme and LPS. The data are presented as the means ± SD; ns, no significant difference; * $p \leq$ 0.05.

Figure S1. Dysfunction of Paneth cells was revealed by the mRNA expression of (A) Defa5, lysozyme, sPLA2, Reg3γ, (B) Lgr5, Wnt3a and TGFβ in three different models of AP. The data are presented as the means ± SD; ns, no significant difference; * $p \leq 0.05$.

Figure S2. The establishment of long term reduction of Paneth cells. (A) Experimental design. Black arrows represent dithizone treatments, red arrows represented induction of AP. (B) Representative ileal immunofluorescence photographs of lysozyme (green) at different time points (200× magnification). Lysozyme+/DAPI+ was also measured at different time points. (C) Mean number of Paneth cells per crypt at different time points. The mRNA expression of (D) Defa5 and (F) lysozyme at different time points. The data are presented as the means ± SD; * $p \leq 0.05$.

Figure S3. (A) *Firmicutes/Bacteroidetes* (F/B) ratio in AP mice and Dith mice with or without AP. (B) F/B ratio in Dith+AP mice and Lyz+Dith+AP mice. (C) Levels of common species in *Helicobacter.spp* and *Blautia. spp*. The data are presented as the means ± SD; ns, no significant difference; * $p \leq 0.05$.

Figure S4. In Dith mice with or without AP, (A) the mRNA expression of ileal lysozyme, Defa5, Reg3γ, MMP7, Reg3β, Ang4, sPLA2 and cryptdin1 were assessed by real time PCR and (B) proteins expression of ileal lysozyme, sPLA2, Reg3γ, Ang4 and Defa5 were assessed by western blot. (C) Heatmap showed a correlation between relative expression of AMPs and levels of proinflammatory factors. Blue means negative correlation, red means positive correlation. In Dith+AP mice with or without AP,

immunofluorescence staining of (D) claudin1, (E) ZO-1 and (F) occludin (green) (200× magnification) and (G) quantification of fluorescence were shown. The data are presented as the means ± SD; ns, no significant difference; * $p \leq 0.05$.

Figure S5. The mRNA expression of (A) lysozyme, (B) Defa5, (C) Reg3γ and (D) Ang4 were carried out to evaluate intestinal permeability. The data are presented as the means ± SD; ns, no significant difference; * $p \leq 0.05$.

Figure S6. The protein quantification of (A) lysozyme, Reg3γ, sPLA2, Ang4, Defa5, (B) Lgr5, Wnt3a, β-catenin, c-myc, occludin and claudin1 were carried out to evaluate protein expression. The data are presented as the means ± SD; ns, no significant difference; * $p \leq 0.05$.

Figure S7. Original blot images of all quantification of western blot. (A) Lgr5, (B) Lgr5, (C) Lgr5, occludin and claudin1, (D) β-catenin and Wnt3a, (E) c-myc, (F) Ang4 and Defa5, (G) lysozyme, (H) Reg3γ, (I) sPLA2.

Table S1. Clinical and demographic characteristics of AP patients.

Table S2. The sequences of the primers used in this study.

Reviewer #1 (Comments for the Author):

The authors have sufficiently:
1. Applied correct analytical models to study statistical differences in cellular populations.
2) Added new novel data in relation to their study to look at Heliobacter sp. opening up new avenues of study.
3) Outlined limitations owing to technical and funding limitations, which is a part of ongoing science.
The authors have therefore sufficiently addressed my concerns. Any typographical errors including syntax, missing references, and grammatical deficiencies can be handled by the handling editor and typesetting editors of this journal.

Reviewer #2 (Comments for the Author):

The reviewer carefully read the responses to the review comment and the revised manuscript. All concerns have been addressed and it is acceptable for publication. Congratulations!

We gratefully thank the reviewers for the time and effort that they have put into reviewing the previous version of the manuscript.

Reviewer #3 (Comments for the Author):

The authors have taken enough care to improve the manuscript by providing the required information. However, there are minor grammatic errors that need to be taken into consideration. The changes are highlighted in the manuscript (pdf version).

We thank the distinguished reviewer for careful review of our revision. In revised manuscript, we have corrected all grammatic errors you mentioned. All changes are highlighted by yellow.

April 8, 2022

Dr. Yue Zeng
Shanghai General Hospital, Shanghai JiaoTong University School of Medicine
Shanghai
China

Re: mSystems01507-21R2 (Paneth cells protect against acute pancreatitis via modulating gut microbiota dysbiosis)

Dear Dr. Yue Zeng:
Thank you for submitting your manuscript to mSystems.

I am pleased to inform you that your manuscript has been accepted, and I am forwarding it to the ASM Journals Department for publication. For your reference, ASM Journals' address is given below. Before it can be scheduled for publication, your manuscript will be checked by the mSystems production staff to make sure that all elements meet the technical requirements for publication. They will contact you if anything needs to be revised before copyediting and production can begin. Otherwise, you will be notified when your proofs are ready to be viewed.

ASM policy requires that data be available to the public upon online posting of the article, so please verify all links to sequence records, if present, and make sure that each number retrieves the full record of the data. If a new accession number is not linked or a link is broken, provide production staff with the correct URL for the record. If the accession numbers for new data are not publicly accessible before the expected online posting of the article, publication of your article may be delayed; please contact the ASM production staff immediately with the expected release date.

As an open-access publication, mSystems receives no financial support from paid subscriptions and depends on authors' prompt payment of publication fees as soon as their articles are accepted.

Publication Fees:
You will be contacted separately about payment when the proofs are issued; please follow the instructions in that e-mail. Arrangements for payment must be made before your article is published. For a complete list of **Publication Fees**, including supplemental material costs, please visit our website.

Corresponding authors may join or renew ASM membership to obtain discounts on publication fees. Need to upgrade your membership level? Please contact Customer Service at Service@asmusa.org.

For mSystems research articles, you are welcome to submit a short author video for your recently accepted paper. Videos are normally 1 minute long and are a great opportunity for junior authors to get greater exposure. Importantly, this video will not hold up the publication of your paper, and you can submit it at any time.

Details of the video are:

· Minimum resolution of 1280 x 720
· .mov or .mp4. video format
· Provide video in the highest quality possible, but do not exceed 1080p
· Provide a still/profile picture that is 640 (w) x 720 (h) max
· Provide the script that was used

We recognize that the video files can become quite large, and so to avoid quality loss ASM suggests sending the video file via https://www.wetransfer.com/. When you have a final version of the video and the still ready to share, please send it to mSystems staff at msystems@asmusa.org.

For mSystems research articles, if you would like to submit an image for consideration as the Featured Image for an issue, please contact mSystems staff at msystems@asmusa.org.

Thank you for submitting your paper to mSystems.

Sincerely,

Chaysavanh Manichanh
Editor, mSystems

Journals Department
American Society for Microbiology
1752 N St., NW
Washington, DC 20036
E-mail: peerreview@asmusa.org
Phone: (202) 737-3600

Fig. S7: Accept
Fig. S6: Accept
Fig. S3: Accept
Fig. S4: Accept
Fig. S1: Accept
Table S1: Accept
Table S2: Accept
Fig. S5: Accept
Fig. S2: Accept
